# Genome-wide meta-analysis and omics integration identifies novel genes associated with diabetic kidney disease

**DOI:** 10.1007/s00125-022-05735-0

**Published:** 2022-06-28

**Authors:** Niina Sandholm, Joanne B. Cole, Viji Nair, Xin Sheng, Hongbo Liu, Emma Ahlqvist, Natalie van Zuydam, Emma H. Dahlström, Damian Fermin, Laura J. Smyth, Rany M. Salem, Carol Forsblom, Erkka Valo, Valma Harjutsalo, Eoin P. Brennan, Gareth J. McKay, Darrell Andrews, Ross Doyle, Helen C. Looker, Robert G. Nelson, Colin Palmer, Amy Jayne McKnight, Catherine Godson, Alexander P. Maxwell, Leif Groop, Mark I. McCarthy, Matthias Kretzler, Katalin Susztak, Joel N. Hirschhorn, Jose C. Florez, Per-Henrik Groop, for the GENIE Consortium

**Affiliations:** 1 Folkhälsan Institute of Genetics, Folkhälsan Research Center, Helsinki, Finland; 2 Department of Nephrology, University of Helsinki and Helsinki University Hospital, Helsinki, Finland; 3 Research Program for Clinical and Molecular Metabolism, Faculty of Medicine, University of Helsinki, Helsinki, Finland; 4 Programs in Metabolism and Medical & Population Genetics, Broad Institute of MIT and Harvard, Cambridge, MA USA; 5 Division of Endocrinology, Boston Children’s Hospital, Boston, MA USA; 6 Diabetes Unit and Center for Genomic Medicine, Massachusetts General Hospital, Boston, MA USA; 7 Michigan Medicine, Ann Arbor, MI USA; 8 Renal, Electrolyte, and Hypertension Division, Department of Medicine, University of Pennsylvania, Perelman School of Medicine, Philadelphia, PA USA; 9 Institute for Diabetes, Obesity, and Metabolism, University of Pennsylvania, Perelman School of Medicine, Philadelphia, PA USA; 10 Department of Genetics, University of Pennsylvania, Perelman School of Medicine, Philadelphia, PA USA; 11 Department of Clinical Sciences, Lund University Diabetes Centre, Lund University and Skåne University Hospital, Malmö, Sweden; 12 Pat Macpherson Centre for Pharmacogenetics & Pharmacogenomics, Cardiovascular & Diabetes Medicine, School of Medicine, University of Dundee, Dundee, UK; 13 Oxford Centre for Diabetes, Endocrinology & Metabolism, Radcliffe Department of Medicine, University of Oxford, Oxford, UK; 14 Wellcome Centre for Human Genetics, Nuffield Department of Medicine, University of Oxford, Oxford, UK; 15 Molecular Epidemiology Research Group, Centre for Public Health, Queen’s University Belfast, Belfast, UK; 16 Herbert Wertheim School of Public Health and Human Longevity Science, University of California San Diego, La Jolla, CA USA; 17 Finnish Institute for Health and Welfare, Helsinki, Finland; 18 Diabetes Complications Research Centre, Conway Institute, School of Medicine, University College Dublin, Dublin, Ireland; 19 Chronic Kidney Disease Section, National Institute of Diabetes and Digestive and Kidney Diseases, Phoenix, AZ USA; 20 Regional Nephrology Unit, Belfast City Hospital, Belfast, Northern Ireland UK; 21 Institute for Molecular Medicine Finland FIMM, University of Helsinki, Helsinki, Finland; 22 Departments of Pediatrics and Genetics, Harvard Medical School, Boston, MA USA; 23 Department of Medicine, Harvard Medical School, Boston, MA USA; 24 Department of Diabetes, Central Clinical School, Monash University, Melbourne, Victoria Australia

**Keywords:** Diabetes complications, Diabetic kidney disease, Genetics, Genome-wide association study; Meta-analysis; Transcriptomics

## Abstract

**Aims/hypothesis:**

Diabetic kidney disease (DKD) is the leading cause of kidney failure and has a substantial genetic component. Our aim was to identify novel genetic factors and genes contributing to DKD by performing meta-analysis of previous genome-wide association studies (GWAS) on DKD and by integrating the results with renal transcriptomics datasets.

**Methods:**

We performed GWAS meta-analyses using ten phenotypic definitions of DKD, including nearly 27,000 individuals with diabetes. Meta-analysis results were integrated with estimated quantitative trait locus data from human glomerular (*N*=119) and tubular (*N*=121) samples to perform transcriptome-wide association study. We also performed gene aggregate tests to jointly test all available common genetic markers within a gene, and combined the results with various kidney omics datasets.

**Results:**

The meta-analysis identified a novel intronic variant (rs72831309) in the *TENM2* gene associated with a lower risk of the combined chronic kidney disease (eGFR<60 ml/min per 1.73 m^2^) and DKD (microalbuminuria or worse) phenotype (*p*=9.8×10^−9^; although not withstanding correction for multiple testing, *p*>9.3×10^−9^). Gene-level analysis identified ten genes associated with DKD (*COL20A1*, *DCLK1*, *EIF4E*, *PTPRN–RESP18*, *GPR158*, *INIP–SNX30*, *LSM14A* and *MFF*; *p*<2.7×10^−6^). Integration of GWAS with human glomerular and tubular expression data demonstrated higher tubular *AKIRIN2* gene expression in individuals with vs without DKD (*p*=1.1×10^−6^). The lead SNPs within six loci significantly altered DNA methylation of a nearby CpG site in kidneys (*p*<1.5×10^−11^). Expression of lead genes in kidney tubules or glomeruli correlated with relevant pathological phenotypes (e.g. *TENM2* expression correlated positively with eGFR [*p*=1.6×10^−8^] and negatively with tubulointerstitial fibrosis [*p*=2.0×10^−9^], tubular *DCLK1* expression correlated positively with fibrosis [*p*=7.4×10^−16^], and *SNX30* expression correlated positively with eGFR [*p*=5.8×10^−14^] and negatively with fibrosis [*p*<2.0×10^−16^]).

**Conclusions/interpretation:**

Altogether, the results point to novel genes contributing to the pathogenesis of DKD.

**Data availability:**

The GWAS meta-analysis results can be accessed via the type 1 and type 2 diabetes (T1D and T2D, respectively) and Common Metabolic Diseases (CMD) Knowledge Portals, and downloaded on their respective download pages (https://t1d.hugeamp.org/downloads.html; https://t2d.hugeamp.org/downloads.html; https://hugeamp.org/downloads.html).

**Graphical abstract:**

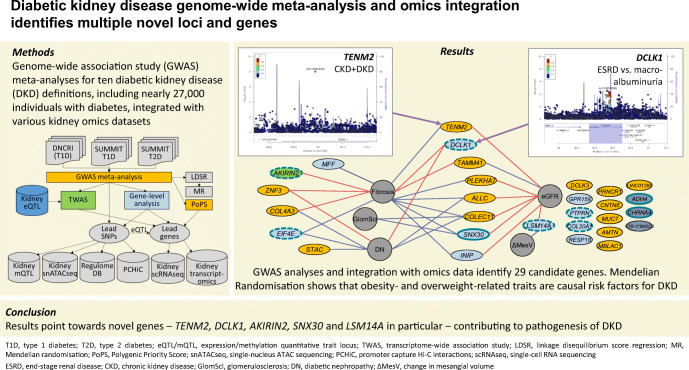

**Supplementary Information:**

The online version contains peer-reviewed but unedited supplementary material available at 10.1007/s00125-022-05735-0.

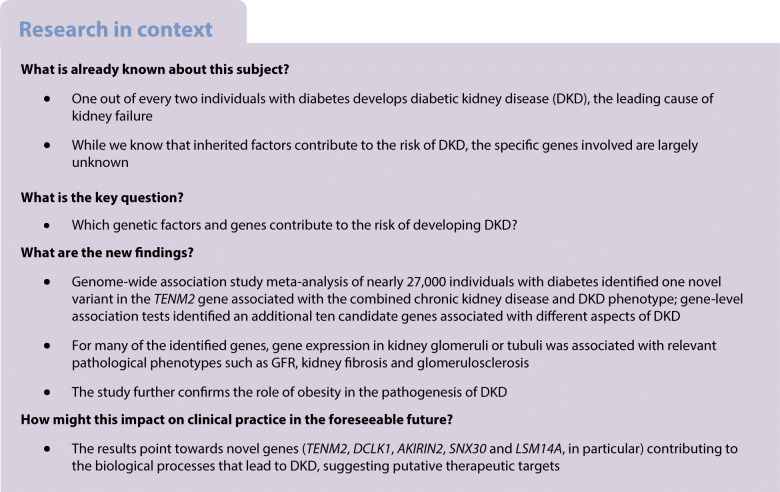



## Introduction

**Fig. 1:**
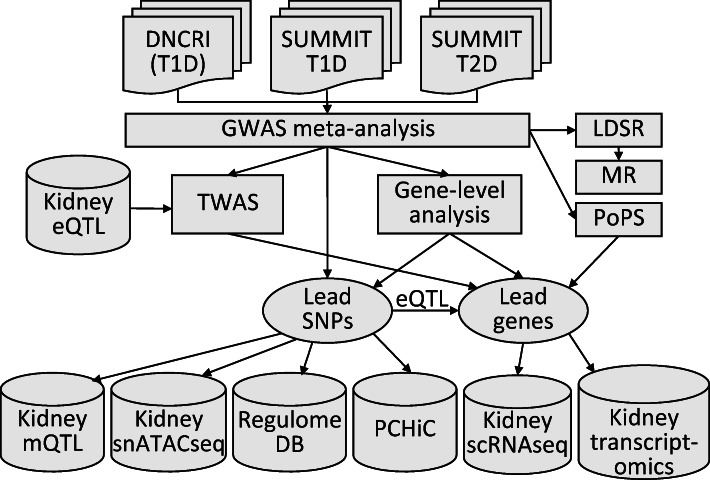
Schematic illustration of the study design, from GWAS meta-analysis to integration with various omics data sets. GWAS meta-analysis for ten different phenotypic definitions of DKD included up to 26,785 individuals with either type 1 or type 2 diabetes from the previous DNCRI and SUMMIT GWAS meta-analyses. The TWAS integrated the GWAS meta-analysis results with kidney eQTL data for tubular and glomerular compartments, identifying genes with differential expression in DKD. The mQTL data identified SNPs associated with DNA methylation at CpG sites. Single nucleus Assay for Transposase-Accessible Chromatin using sequencing (snATACseq) was informative of chromatin openness in various kidney cell types. The RegulomeDB is a database with extensive epigenetic annotation for SNPs. The promoter capture HiC (PCHiC) sequencing data identified sequence interaction with gene promoters, proposing target genes. Kidney transcriptomics provided data on gene expression in glomerular and tubular tissue in nephrectomy samples, or in Pima Indian biopsies, correlated with various renal variables. scRNAseq, single-cell RNA sequencing; T1D, type 1 diabetes; T2D, type 2 diabetes

Diabetes is the leading cause of kidney disease. Diabetic kidney disease (DKD) is associated with high cardiovascular risk [1] and mortality [2] and, consequently, both diabetes and kidney disease are leading causes of death worldwide [3]. Both environmental and genetic factors have a major impact on the risk of developing DKD [4, 5]. Although more than 300 genetic loci have been associated with chronic kidney disease (CKD) in the general population, these loci show limited effect in DKD, especially in individuals with type 1 diabetes [6]. Genome-wide association studies (GWAS) have previously identified a handful of genetic loci for DKD at the genome-wide significance level (*p*<5×10^−8^) [7–11]. Recently, a meta-analysis of GWAS, including up to 19,406 individuals with type 1 diabetes from the Diabetic Nephropathy Collaborative Research Initiative (DNCRI), identified 16 loci. The strongest association was a common missense variant in the *COL4A3* gene, which also showed evidence of association in individuals with type 2 diabetes [6]. A GWAS meta-analysis from The SUrrogate markers for Micro- and Macrovascular hard endpoints for Innovative diabetes Tools (SUMMIT) consortium, including 6000 individuals with type 2 diabetes from five different studies, identified three loci for DKD, including *UMOD* and *PRKAG2* previously identified in the general population [12]. However, meta-analysis with SUMMIT type 1 diabetes studies (SUMMIT-1, *N*=5156) did not yield any genome-wide significant findings. To increase the power to detect novel genetic risk factors for DKD shared among diabetes subtypes, we aggregated all available data for DKD in individuals of European ancestry with type 1 or type 2 diabetes (*N*~27,000). Specifically, we performed GWAS meta-analyses on ten different DKD case–control definitions, meta-analysing summary statistics from DNCRI [6], SUMMIT-1 [4] and SUMMIT type 2 diabetes studies (SUMMIT-2) [12], followed by integration with diverse biological data to improve our understanding of the underlying biological mechanisms and clinical correlations (Fig. 1).

## Methods

For detailed methods, please refer to the electronic supplementary material (ESM) Methods.

### Participating studies and phenotype definitions

A total of ten case–control definitions for DKD were included in DNCRI [6], based on either urinary AER (divided into controls with normal AER, and cases with microalbuminuria, macroalbuminuria or end-stage renal disease [ESRD]), eGFR, or both, and harmonised to match and include all seven phenotypic definitions assessed in SUMMIT-1 [4] and SUMMIT-2 [12] analyses (ESM Table 1). All individuals (both cases and controls) had diabetes (either type 1 or type 2 diabetes). For three phenotypic comparisons not initially part of the SUMMIT analysis, GWAS and meta-analysis were performed with three SUMMIT-2 studies and the Scania Diabetes Registry type 1 diabetes cohort. Individuals from the Finnish Diabetic Nephropathy Study (FinnDiane) were included in both the original DNCRI (*N*=6019) and SUMMIT-1 analyses (*N*=3415) and thus were excluded here from the SUMMIT-1 data (ESM Table 2). All contributing studies were performed in accordance with the Declaration of Helsinki and Declaration of Istanbul.

### Genome-wide association study and meta-analysis

Genotyping and statistical analysis of the DNCRI [6] and SUMMIT [4, 12] cohorts have been previously described. Analysis plans were similar in the cohorts (ESM Table 3). Imputation was performed using 1000Genomes Phase 3 reference panel in DNCRI, and the older 1000Genomes Phase I panel in the SUMMIT cohorts. Analyses were performed in unrelated individuals using the SNPtest additive score test, adjusting for age, sex, diabetes duration, the genetic principal components, and study-specific covariates (e.g. site or genotyping batch). Variants were filtered for INFO imputation quality score ≥0.3 (DNCRI) or ≥0.4 (SUMMIT) and minor allele count ≥10 in both cases and controls. In SUMMIT, variants were further filtered to those with minor allele frequency (MAF) ≥0.01. Meta-analyses of DNCRI, SUMMIT-1 and SUMMIT-2 summary statistics were performed with inverse variance fixed effect methods based on the effect sizes. Variants were limited to those found in at least two studies.

Power calculations indicated 80% power to detect associations with *p*<5×10^−8^ and an OR of 1.20 for the combined ‘all vs ctrl’ phenotype for common variants with MAF≥10%, or with an OR of 1.28 and 1.73 for low-frequency (MAF 5%) and rare (MAF 1%) variants, respectively (ESM Fig. 1).

Correction for multiple testing was estimated with spectral decomposition of the ten DKD traits, suggesting 5.36 effective tests, leading to a corrected significance threshold of *p*<9.3×10^−9^.

### Gene prioritisation analysis

Gene prioritisation at the top loci was performed using two complementary similarity-based gene prioritisation approaches (Polygenic Priority Score [PoPS] v0.1 [13] and MAGMA v1.06b [14]), which integrate GWAS with gene set enrichment based on a variety of biological annotation datasets.

### Gene-level analysis

SNPs from the GWAS meta-analysis summary statistics were aggregated by gene-level regression analysis using two related programs, MAGMA v1.06b [14] and PASCAL v2016 [15], using default parameters. Gene-level significance thresholds were determined by a Bonferroni multiple testing correction based on the number of genes tested for each of the ten phenotypes (from *p*<2.7×10^−6^ to *p*<2.3×10^−6^).

### Transcriptome-wide association study

In the transcriptome-wide association study (TWAS), MetaXcan [16] was applied with default parameters to integrate GWAS meta-analysis results with kidney expression quantitative trait locus (eQTL) datasets for micro-dissected human glomerular (*N*=119) and tubular (*N*=121) samples [17]. Significance threshold of *p*<4.1×10^−6^ was determined by Bonferroni correction for two tissues and 6050 genes found in either tubular or glomerular eQTL data.

### Kidney eQTL, methylation quantitative trait loci, and colocalisation analysis

Kidney-specific eQTL associations were queried for glomeruli [17], tubules [17], and a meta-analysis of four eQTL studies with 451 kidney samples [17–20]. Kidney methylation quantitative trait locus (mQTL) associations were sought in 188 healthy kidney samples profiled by the Infinium MethylationEPIC Kit and BeadChips (Illumina, USA), with *p*<1.5×10^−11^ considered significant. For the significant CpG sites, we tested association with DKD in our epigenome-wide association study (EWAS) of 1304 All Ireland-Warren 3-Genetics of Kidneys in Diabetes (GoKinD) United Kingdom (UK-ROI) collection and FinnDiane participants, analysed using the Infinium MethylationEPIC Kit and BeadChips, as previously described [21]. To estimate posterior probability that the GWAS association colocalised with the kidney eQTL and mQTL signals, we performed Bayesian multiple-trait-colocalisation analysis, with posterior probability >0.8 considered evidence of colocalisation.

### Human kidney gene expression

For the 29 lead genes, we studied gene expression in kidneys in human transcriptomics data from nephrectomy samples (433 tubule and 335 glomerulus samples) [22] and kidney biopsies from the Pima Indian cohort (67 glomerular and 47 tubulointerstitial tissues) [23], and tested for correlation with relevant pathological phenotypes. The micro-dissected nephrectomy samples were from individuals with varying degree of diabetic and hypertensive kidney disease, and gene expression was defined with RNA sequencing. The study was approved by the institutional review board of the University of Pennsylvania.

In the Pima Indian cohort, gene expression profiling in the first biopsy was performed with Affymetrix gene chip arrays [23], and with Illumina RNA sequencing for the second biopsy [6]. Available phenotypes included progression to ESRD, measured GFR (mGFR), albumin/creatinine ratio (ACR), HbA_1c_ and six kidney morphological variables for both biopsies, and change in the phenotypes between the first and the second study biopsies (27 phenotypes in total [24]). The study was approved by the Institutional Review Board of the National Institute of Diabetes and Digestive and Kidney Diseases.

### Linkage disequilibrium score regression and Mendelian randomisation

Linkage disequilibrium (LD) score regression (LDSR) [25] was performed at LDhub (http://ldsc.broadinstitute.org/, accessed 22 August 2019) between our 10 DKD GWAS and 78 glycaemic, autoimmune, anthropometric, bone, smoking behaviour, lipid, kidney, uric acid, cardiometabolic and ageing-related traits (ESM Table 4). LDSR associations with Bonferroni-adjusted *p*<6.4×10^−4^ were considered significant. To identify causal relationships for significant traits in the LDSR against DKD, we performed summary-based two-sample Mendelian randomisation (MR) with inverse variance-weighted regression implemented in TwoSampleMR v0.5.6 R package [26]. Causality was further assessed using methods less sensitive to pleiotropy/heterogeneity [27].

## Results

### GWAS meta-analysis

The GWAS meta-analysis of the DNCRI (type 1 diabetes), and SUMMIT-1 and SUMMIT-2 meta-analyses included up to 26,785 individuals with either type 1 or type 2 diabetes from 25 studies; 11,380 individuals had any DKD (micro- or macroalbuminuria or ESRD) and 15,405 individuals had normal AER (ESM Table 2). QQ plots, λ genomic control inflation factor (λ_GC_) and LDSR intercepts of the meta-analysis indicated no marked inflation or population stratification bias of the results (ESM Fig. 2).

**Fig. 2:**
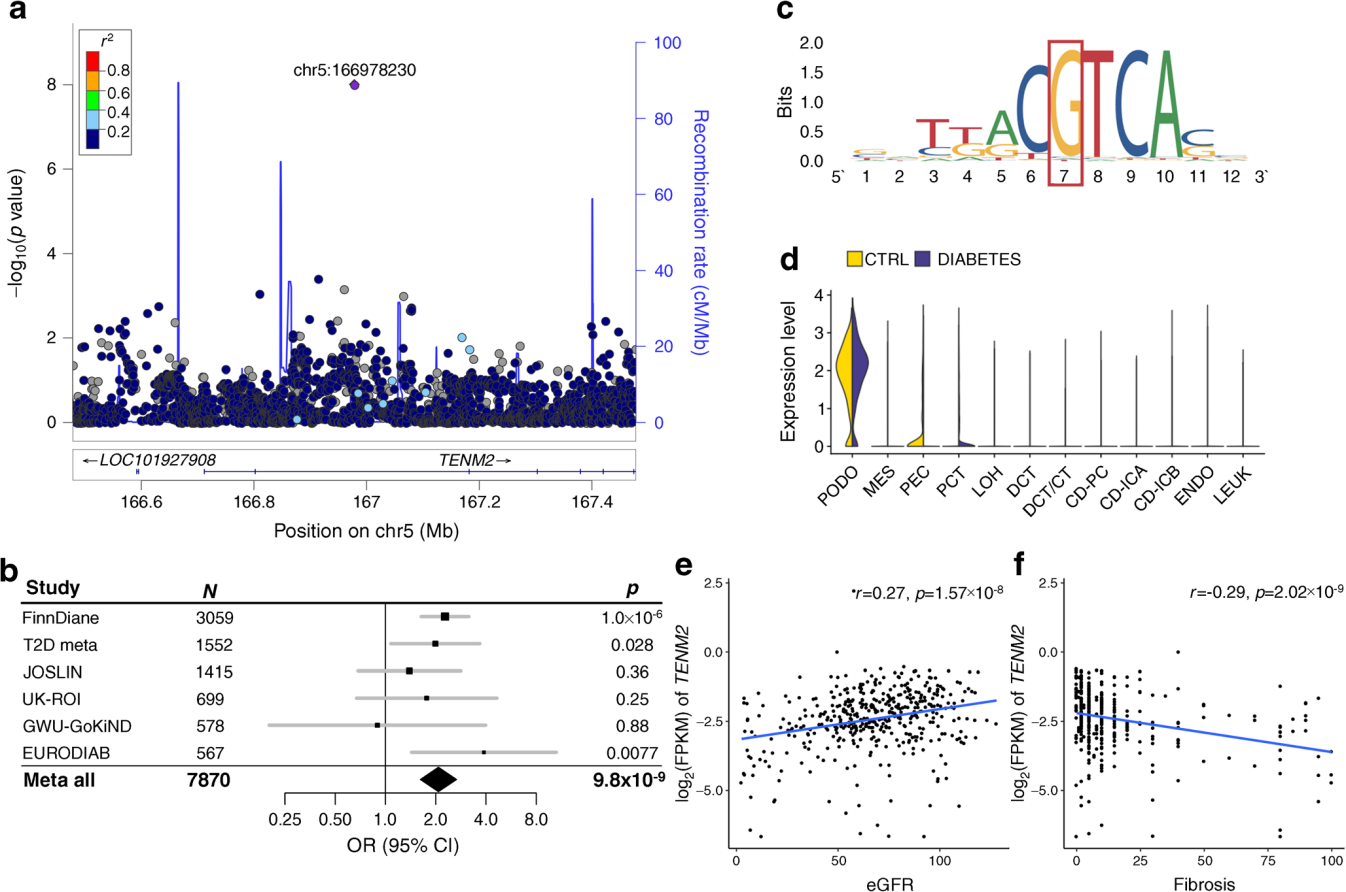
*TENM2* gene rs72831309 is associated with CKD–DKD. (**a**) Regional association plot of the meta-analysis results. (**b**) Forest plot of association across the contributing cohorts from DNCRI (FinnDiane, JOSLIN, UK-ROI, GWU_GoKinD) [6], SUMMIT-T1D (EURODIAB) [4] and SUMMIT-T2D studies [12]. (**c**) SNP rs72831309 overlaps a predicted CREB1 binding motif sequence; data from RegulomeDB.org (v.2.0.3). (**d**) Human kidney single-cell RNA expression of *TENM2*, showing strongest expression in podocytes, parietal epithelial cells and proximal convoluted tubules. (**e**, **f**) Tubular *TENM2* expression is correlated with higher eGFR (**e**) and less fibrosis (**f**). CD, collecting duct; CT, connecting tubule; CTRL, control; DCT, distal convoluted tubule; ENDO, endothelium; FPKM, fragments per kilo base of transcript per million mapped fragments; GWU_GoKinD, George Washington University Genetics of Kidney in Diabetes; IC, intercalated cell (A/B); JOSLIN, Joslin Diabetes Center participants; LEUK, leucocyte; LOH, loop of Henle; MES, mesangial cells; PC, principal cell; PCT, proximal convoluted tubule; PEC, parietal epithelial cells; PODO, podocytes; T2D meta, meta-analysis of type 2 diabetes cohorts

The meta-analysis identified a novel association between the combined CKD–DKD phenotype (cases with eGFR <45 ml/min per 1.73 m^2^ and microalbuminuria or worse, vs controls with normal AER and eGFR ≥60 ml/min per 1.73 m^2^) and rs72831309 (MAF=4%; OR 2.08, *p*=9.8×10^−9^; Fig. 2a, Table 1 and ESM Table 5). Of note, the association was barely above the threshold after correction for multiple testing due to multiple phenotypes (*p*>9.3×10^−9^). We observed no heterogeneity between individuals with type 1 or type 2 diabetes (*p*_HET_=0.88). The variant is located in an intron of the *TENM2* gene encoding the teneurin transmembrane protein 2. The variant was imputed with moderate imputation quality across cohorts (INFO score 0.38–0.66). In the FinnDiane cohort with the strongest statistical significance, the association remained (though slightly attenuated) after re-imputation with a population-specific panel (INFO=0.92, *p*=2.0×10^−4^, OR 1.70 [95% CI 1.28, 2.24] vs INFO=0.66, *p*=1.0×10^−6^, OR 2.27 [95% CI 1.64, 3.16]).

**Table 1:** GWAS meta-analysis result summary for loci with *p*<5×10^−8^

Phenotype	CHR:POS	SNP	EA	NEA	EAF	OR (95% CI)	*p* value	Dir	*N* (studies)	Genes
Novel locus										
CKD+DKD	5:166978230	rs72831309	A	G	0.039	2.08 (1.62, 2.67)	9.8×10^−9^	+++	8570 (7)	*TENM2*^a,b^
Previous loci										
CKD	2:3745215	rs12615970	A	G	0.867	1.31 (1.20, 1.44)	9.4×10−^9^	+??	18,488 (13)	*ALLC*^b^, *COLEC11*
All vs Ctrl	2:228121101	rs55703767	T	G	0.207	0.86 (0.82, 0.90)	1.9×10^−9c^	−+−	26,898 (24)	*COL4A3*^a,b^
CKD+DKD	2:228121101	rs55703767	T	G	0.210	0.81 (0.75, 0.88)	4.7×10^−8^	−+−	17,611 (17)	*COL4A3*^a,b^
Severe DKD	2:228121101	rs55703767	T	G	0.208	0.82 (0.77, 0.87)	3.6×10^−11c^	−−−	21,898 (23)	*COL4A3*^a,b^
ESRD	3:926345	rs115061173	A	T	0.014	9.40 (4.22, 20.93)	4.1×10^−8^	+??	4827 (3)	*LINC01266*^b^, *CNTN6*^a^
Micro	3:11910635	rs142823282	A	G	0.983	0.15 (0.08, 0.27)	8.3×10^−10c^	−??	6076 (2)	*TAMM41*
ESRD vs all	3:36566312	rs116216059	A	C	0.016	8.73 (4.13, 18.45)	1.4×10^−8^	+??	3667 (2)	*STAC*^b^, *DCLK3*
Severe DKD	4:71358776	rs191449639	A	T	0.005	32.42 (9.77, 107.59)	1.3×10^−8^	+??	7768 (2)	*MUC7*, *AMTN*
Micro	7:99728546	rs77273076	T	C	0.008	9.16 (4.29, 19.56)	1.1×10^−8^	+??	7500 (2)	*MBLAC1*, *ZNF3*
ESRD vs macro	8:128100029	rs551191707	CA	C	0.122	1.69 (1.40, 2.04)	4.4×10^−8^	+??	3634 (7)	*PRNCR1*^b^
Micro	11:16937846	rs183937294	T	G	0.993	0.06 (0.02, 0.16)	1.7×10^−8^	−??	6076 (2)	*PLEKHA7*^a,b^
CKD	18:1811108	rs185299109	T	C	0.007	20.75 (7.30, 59.00)	1.3×10^−8^	+??	7223 (2)	*LINC00470*

^a^Gene prioritised by PoPS

^b^Genes underlying the lead SNP

^c^*p*<9.3×10^−9^ (i.e. corrected for 5.36 effective tests [phenotypes])

CHR:POS, variant chromosome and basepair position; Ctrl, control; Dir, direction of association in DNCRI (type 1 diabetes), SUMMIT-2 (type 2 diabetes) and in SUMMIT-1 (type 1 diabetes), respectively; EA, effect allele; EAF, effect allele frequency; Genes, closest gene(s); Micro, microalbuminuria vs normal AER; NEA, non-effect allele; *N* (studies), number of contributing individuals and studies

At the previously identified *COL4A3* locus, we identified a secondary association peak (rs6436688, *p*=1.8×10^−7^ for severe DKD; ESM Fig. 3) in only partial LD (*D*′=0.51, *r*^2^=0.08) with the lead variant rs55703767. The association at rs6436688 remained nominally significant after conditional analysis for rs55703767 (*p*=0.002).

In addition to the *COL4A3* locus, nine other previously identified, mostly low-frequency or rare variants were associated with various kidney phenotypes (Table 1 and ESM Fig. 4). None of these variants were found in the SUMMIT meta-analyses (filtered to MAF≥1%), and thus, these associations represent the originally reported associations from the DNCRI [6]. One common (chr14q12), and four of our previously identified rare DNCRI loci (*TAMM41*, *HAND2–AS1*, *DDR1–VARS2*, *BMP7*; MAF~1%) associated with microalbuminuria demonstrated attenuated association when combined with the SUMMIT meta-analyses (with the rare variants only found in SUMMIT-2). The lack of replication across diabetes subtypes suggest either false positives or a lack of power to detect an association for rare variants in individuals with type 2 diabetes DKD (a more heterogeneous disease, particularly for the early stages of DKD [i.e. microalbuminuria]). Alternatively, this could simply represent a lack of shared biology across diabetes subtypes, again possibly due to the different underlying causes of kidney damage in individuals with type 1 vs type 2 diabetes.

Two variants, chr3:141792314:I and rs186434345, were associated with ESRD (*N*=940, *p*=4.6×10^−10^), and with the CKD–DKD phenotype (*N*=2571, *p*=4.0×10^−8^) in the SUMMIT-2 and SUMMIT-1 cohorts, but were absent in DNCRI. When the original SUMMIT–FinnDiane GWAS was included in the analysis, both associations were non-significant (chr3:141792314:I, *p*=0.056, *N*=3207; rs186434345, *p*=0.002, *N*=4782) and thus excluded from further consideration.

### Gene prioritisation

To identify the underlying causal genes within each of our top loci, we used the PoPS [13] method that leverages genome-wide enrichment of biological annotations in combination with GWAS summary statistics to prioritise candidate genes. To increase precision, we intersected the results with both the simple nearest-gene approach and MAGMA gene prioritisation. Four genes (*COL4A3*, *PLEKHA7*, *CNTN6* and *TENM2*) were both the PoPS prioritised gene and the nearest protein coding gene to the lead SNP (Table 1). Of note, the *CNTN6* locus contained only two protein coding genes and the *TENM2* locus only one. When taking the intersect between PoPS genes and genes that were within MAGMA’s top 10% of prioritised genes genome-wide, *COL4A3* was the only prioritised gene (ESM Fig. 5). The gene set that prioritised *COL4A3* for severe DKD was the fibulin 2 protein–protein interaction network (‘FBLN2 PPI subnetwork’), which together with 26 correlated reconstituted gene-sets makes up the ‘basement membrane’ meta-gene set derived in Marouli et al [28] (ESM Table 6).

### Gene-level analysis

To improve power and jointly test all available common genetic markers within a gene, SNPs from the GWAS meta-analysis summary statistics were aggregated by gene and tested jointly for association using two similar programs, MAGMA and PASCAL. In addition to *COL20A1* and *SNX30* identified previously [6], we identified eight novel gene associations (*p*<2.7×10^−6^; Table 2 and ESM Fig. 6). The lead variants in these loci indicated no significant heterogeneity between type 1 and type 2 diabetes apart from the *GPR158* locus (*p*_HET_=0.005; ESM Table 7). MAGMA’s gene-level analysis type 1 error was well controlled, with all but one λ_GC_ inflation factor under 1.05 (MAGMA’s ESRD vs all λ_GC_=1.07). The genome-wide gene-level results from PASCAL showed slightly more inflation (λ_GC_ up to 1.15 for ESRD vs all).

**Table 2:** Significant gene-level DKD association results from MAGMA and PASCAL

Phenotype	Gene	Chr	bp start	bp end	*N* SNPs	*p* value	Method	Genes
CKD	*PTPRN*	2	220149345	220179295	72	2.13×10^−6^	MAGMA	18,461
CKD	*RESP18*	2	220187131	220202899	62	2.27×10^−6^	MAGMA	18,461
Severe DKD	*MFF*	2	228189866	228222552	439	2.07×10^−6^	PASCAL	21,790
ESRD vs macro	*EIF4E*	4	99794607	99856786	150	5.79×10^−7^	MAGMA	18,442
ESRD vs macro	*EIF4E*	4	99799606	99851786	269	9.28×10^−7^	PASCAL	21,762
All vs Ctrl	*INIP*	9	115443786	115485387	111	4.89×10^−7^	MAGMA	18,475
All vs Ctrl	*INIP*	9	115448790	115480387	248	1.87×10^−6^	PASCAL	21,784
All vs Ctrl	*SNX30*	9	115506911	115642267	505	1.30×10^−6^	MAGMA	18,475
Severe DKD	*GPR158*	10	25459290	25896158	1875	1.63×10^−6^	MAGMA	18,467
ESRD vs macro	*DCLK1*	13	36337789	36710514	1162	1.39×10^−6^	MAGMA	18,442
Severe DKD	*LSM14A*	19	34658352	34725420	180	1.90×10^−6^	MAGMA	18,467
CKD extremes	*COL20A1*	20	61919538	61967285	146	1.94×10^−7^	MAGMA	18,440
ESRD vs all	*COL20A1*	20	61919538	61967285	145	5.26×10^−7^	MAGMA	18,439

bp start/end, bp position of the start and the end of the gene region; Ctrl, control; Genes, number of genes tested; macro, macroalbuminuria; *N* SNPs, no. of SNPs in the gene region

### Integration of GWAS with kidney eQTL data

**Fig. 3:**
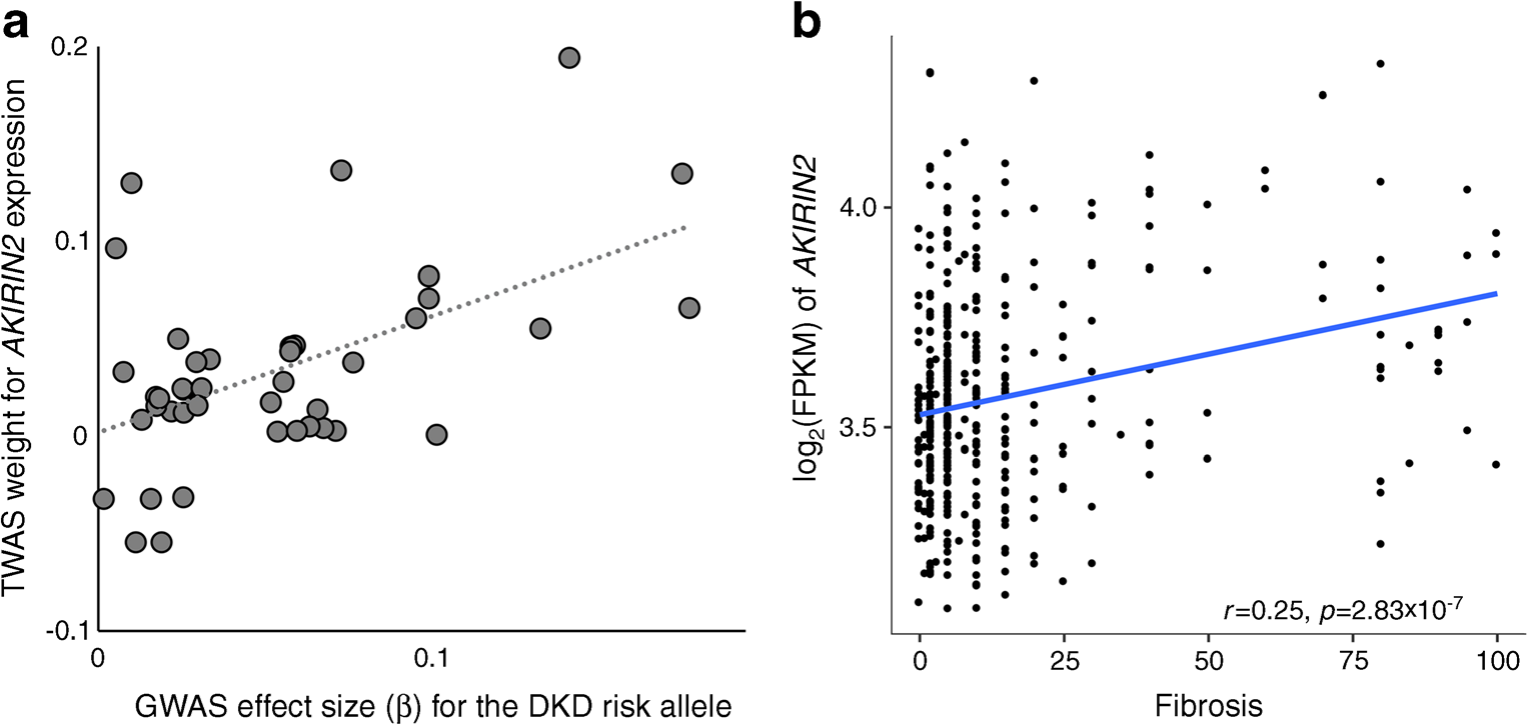
TWAS indicates increased *AKIRIN2* expression in severe DKD. (**a**) The GWAS SNP effect sizes for association with severe DKD (normal AER vs macroalbuminuria or ESRD) are correlated with TWAS eQTL weights to predict *AKIRIN2* expression, suggesting that elevated *AKIRIN2* levels in tubules are associated with severe DKD (*p*=1.1×10^−6^). The eQTL data for 39 SNPs explained 5% of the variance in tubular *AKIRIN2* expression (*p*=0.01). (**b**) *AKIRIN2* expression is correlated with renal fibrosis. FPKM, fragments per kilo base of transcript per million mapped fragments

We performed TWAS for each of the ten DKD meta-analyses to predict differential gene expression between cases and controls based on eQTL data in glomerular and tubulointerstitial samples from histologically normal kidneys [17]. The type 1 error was well controlled (λ_GC_ 0.968–1.097; ESM Fig. 7). Expression levels of *AKIRIN2* were predicted to be higher in the tubular tissue of cases with severe DKD (or macroalbuminuria alone), as compared with controls with normal AER (*p* values 1.1×10^−6^ and 1.7×10^−6^, respectively; Fig. 3a and ESM Tables 8, 9).

### Kidney eQTL and mQTL associations

Kidney eQTL and mQTL data were queried for the top three variants at each lead locus from the GWAS meta-analyses and gene-level analyses. Kidney eQTL data suggested *SNX30* as the target gene in the *INIP–SNX30* region (rs786959 eQTL *p*=4.6×10^−7^), and the GWAS association colocalised with the eQTL signal with moderate evidence (posterior probability 0.70; ESM Table 10). Altogether, 17 variants were significantly associated with kidney DNA methylation levels at six CpG sites (*p*<1.5×10^−11^; ESM Table 11), of which the mQTL colocalised with the GWAS association in *LSM14A*, *DCLK1* and *COL20A1* (posterior probability for colocalisation >0.80). SNPs in the *LSM14A* gene were associated with severe DKD and cg14143166 methylation levels (*p*=1.9×10^−28^). Interestingly, cg14143166 methylation in blood was nominally associated with DKD status in our EWAS in the UK-ROI and FinnDiane cohorts (*p*=0.03), suggesting that the DKD association at *LSM14A* is mediated through methylation changes. Similarly, blood methylation levels at significant kidney mQTL CpG sites (rs7664964–cg25974308 *p*=1.1×10^−11^) in *EIF4E* were nominally associated with eGFR slope in diabetes (*p*=0.04) [29].

### Gene expression and pathological phenotypes

**Fig. 4:**
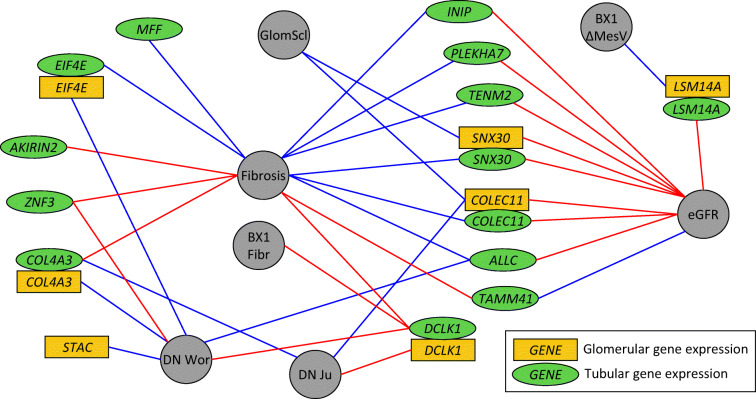
Tubular and glomerular gene expression of the lead genes correlates with multiple morphological and pathological renal variables and with DKD. Golden rectangles indicate glomerular gene expression, green ellipses tubular gene expression, and grey circles the morphological phenotypes. Blue lines indicate negative correlation and red lines indicate positive correlation. Correlation with fibrosis, glomerulosclerosis (GlomScl) and eGFR were measured in the nephrectomy samples [22]; correlations with *p*<2.2×10^-4^ (corrected for 29 genes, two tissues and four tests) are shown. For the biopsy data in Pima Indians, suggestive correlations with *p*<8.6×10^−4^ are shown (corrected only for 29 genes and two tissues), including fibrosis at first biopsy and change in the mesangial volume between the first and the second biopsies. Association with DKD (diabetic nephropathy) was queried in two data sets (Woroniecka et al [36] and Ju et al [35]), with *p*<4.3×10^−4^ or *p*<0.05 and fold change>1.5. BX1 Fibr, fibrosis at first biopsy; BX1 ΔMesV, change in the mesangial volume between the first and the second biopsies; DN Wor, diabetic nephropathy in Woroniecka et al [36]; DN Ju, diabetic nephropathy in Ju et al [35]; GlomScl, glomerulosclerosis

Altogether, we identified 29 lead genes or transcripts from GWAS, gene prioritisation, gene-level analyses, kidney eQTL data or TWAS. Among these, the expression levels of 14 genes significantly correlated with eGFR, glomerulosclerosis or fibrosis in transcriptomics data obtained from 433 tubular and 335 glomerular nephrectomy samples with varying degree of diabetic and hypertensive kidney disease (*p*<2.2×10^−4^; Fig. 4 and ESM Table 12) [22]. For example, tubular *TENM2* expression correlated positively with eGFR (*p*=1.6×10^−8^; Fig. 2e) and negatively with tubulointerstitial fibrosis (*p*=2.0×10^−9^; Fig. 2f), tubular *DCLK1* expression correlated positively with fibrosis (*p*=7.4×10^−16^; Fig. 7c), and tubular *SNX30* expression correlated positively with eGFR (*p*=5.8×10^−14^) and negatively with fibrosis (*p*<2.0×10^−16^). In the Pima Indian kidney biopsy data, tubular *DCLK1* expression was suggestively correlated (*p*<8.6×10^-4^, corrected for 29 genes and two tissues) with higher level of fibrosis, and *LSM14A* negatively correlated with the change in mesangial volume between the two study biopsies (non-significant after further conservative correction for 27 tested phenotypes). Multiple genes were nominally (*p*<0.05) correlated with these renal variables (ESM Fig. 8, ESM Table 13).

### Genetic correlation of DKD between type 1 and type 2 diabetes and general population kidney traits

**Fig. 5:**
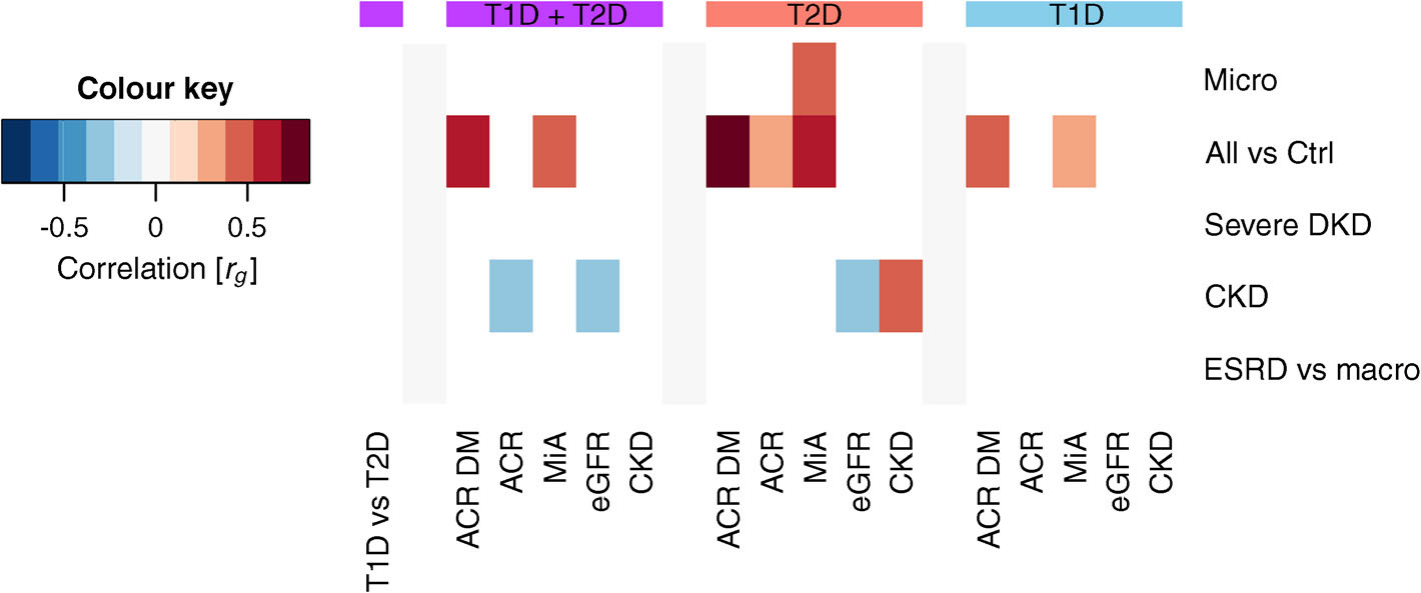
Genetic correlation between DKD phenotypes (*y*-axis) and kidney phenotypes in the general population (*x*-axis). Correlations were calculated with LD score regression for the whole meta-analysis (any diabetes, purple), type 2 diabetes only (red), and type 1 diabetes only (blue). The first column (purple) indicates genetic correlation for the DKD phenotypes between individuals with type 1 or type 2 diabetes (none significant). Only significant correlations (*p*<0.01) are shown. General population GWAS results were taken from CKDgen consortium: ACR [30]; ACR in diabetes [30]; microalbuminuria [30]; eGFR [32]; and CKD [31]. ACR DM, ACR in diabetes; Ctrl, control; ESRD vs macro, ESRD vs macroalbuminuria comparison; MiA, microalbuminuria; Micro, microalbuminuria (in current study); T1D, type 1 diabetes; T2D, type 2 diabetes

We performed LDSR to study the genetic correlation of DKD traits between individuals with type 1 and type 2 diabetes but, likely due to limited sample size, no significant correlations were observed. When compared with the kidney traits from the CKDgen consortium, the ‘All vs Ctrl’ phenotype was correlated with microalbuminuria in the general population [30] and ACR in diabetes [30], both in the main meta-analysis and for type 1 and type 2 diabetes separately (*p*<0.01). In addition, microalbuminuria in type 2 diabetes was correlated with microalbuminuria in the general population [30], and CKD in type 2 diabetes was positively correlated with CKD in the general population [31] and negatively with eGFR in the general population [32]; these were not significantly correlated in individuals with type 1 diabetes despite a larger number of samples (Fig. 5).

### Genetic correlation and MR with related traits

**Fig. 6:**
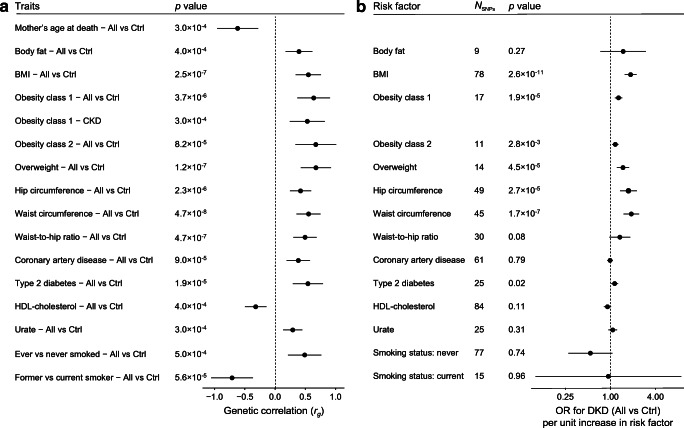
Genetic correlation between DKD phenotypes and various traits based on LDSR, and estimates of causal associations based on MR. (**a**) For LDSR only significant trait combinations are shown (*p*<0.05/78=6.4×10^−4^). (**b**) MR results for DKD (All vs Ctrl comparison) with inverse variance-weighted method for the traits significant in LDSR (‘mother’s age at death’ had fewer than than five genome-wide significant SNPs and thus, was not included in MR). Horizontal bars represent 95% CI. Ctrl, control

LDSR of related metabolic traits revealed significant genetic correlation (*p*<6.4×10^−4^) between DKD and 15 traits including multiple obesity-related traits, mother’s age at death, type 2 diabetes, coronary artery disease, HDL-cholesterol, urate, and two smoking-related traits (Fig. 6 and ESM Fig. 9). MR of these traits suggested that being overweight or obese was a causal risk factor for DKD (Fig. 6b and ESM Table 14). The causal effects were directionally consistent across methods, with no evidence of heterogeneity (*I*^2^=0–42.9%, *p*>0.05; ESM Table 14) or unbalanced horizontal pleiotropy (ESM Table 15). The MR Egger method, more robust for pleiotropic effects, further supported a causal role for higher BMI, waist circumference and hip circumference in DKD risk (*p*<0.05; ESM Table 14, ESM Fig. 10).

## Discussion

We have performed the largest GWAS meta-analysis to date on kidney complications in diabetes, including ten different phenotypic definitions in up to 26,785 individuals with either type 1 or type 2 diabetes, and integrated the results with emerging kidney omics data (Fig. 1). In the single-variant analysis with the combined CKD–DKD phenotype, we identified one novel locus, rs72831309, intronic in *TENM2*. *TENM2* encodes the teneurin transmembrane protein 2 involved in cell–cell adhesion. The variant rs72831309 alters a predicted *CREB1* transcription factor binding site (Fig. 2c), and is nominally associated with expression of a *TENM2* antisense transcript *TENM2-AS1* in kidneys (*p*=0.007; ESM Table 10). Furthermore, chromatin conformation data in the GM12878 cell line indicated that the rs72831309 region interacts with the *TENM2* transcription start site, as well as with three antisense transcripts (*CTB-180C19.1* [Ensembl ENSG00000254365], *CTB–105L4.2* [Ensembl ENSG00000253527] and *CTB–78F1.1* [Ensembl ENSG00000254187]) within the *TENM2* gene [33]. Whereby kidney single-cell RNA sequencing indicated *TENM2* expression particularly in podocytes (Fig. 2d) [34], lower tubular *TENM2* expression was associated with renal fibrosis (*p*=2.0×10^−9^) and lower eGFR (*p*=1.6×10^−8^) in the nephrectomy samples. Despite multiple supporting lines of evidence, the locus still needs further validation as the imputation quality of rs72831309 was on the low end across our cohorts (0.38–0.66), and the association did not remain significant after correction for multiple testing (*p*>9.3×10^–9^).

**Fig. 7:**
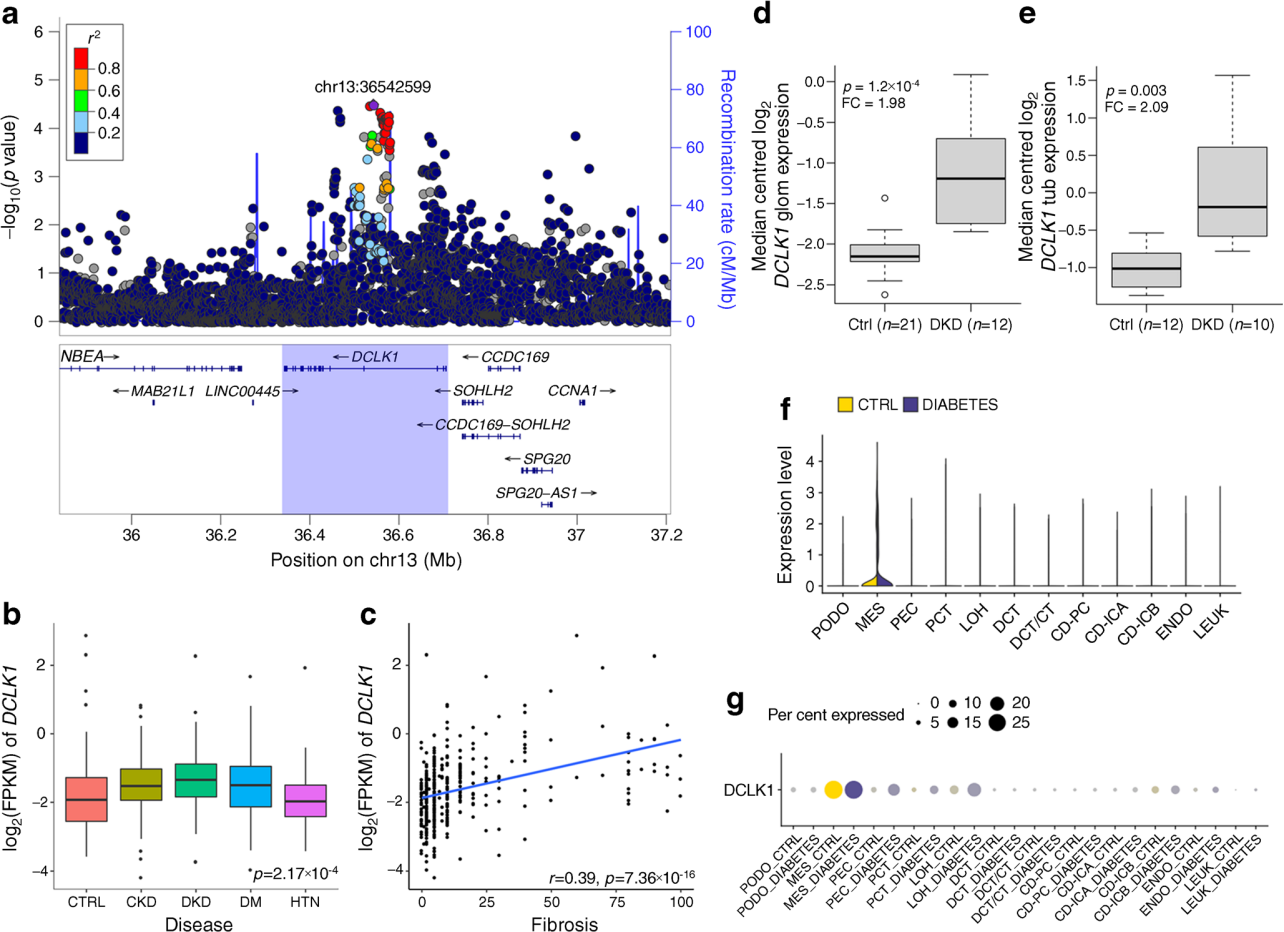
*DCLK1* is associated with ESRD. (**a**) The *DCLK1* gene region was associated with ESRD vs macroalbuminuria in the MAGMA gene-level analysis (*p*=1.39×10^−6^). (**b**, **c**) Tubular *DCLK1* expression is highest in DKD (*p*=2.17×10^−4^) (**b**) and correlated with the level of fibrosis (**c**) in the nephrectomy samples. (**d**) Glomerular *DCLK1* expression is higher in DKD than in healthy controls (Ju et al [35]: fold change 1.98, *p*=1.2×10^−4^). (**e**) Tubular *DCLK1* expression is higher in DKD than in healthy controls (Woroniecka et al [36]: fold change 2.09, *p*=0.003). (**f**, **g**) Kidney *DCLK1* expression is strongest in mesangial cells in human single-cell RNA sequencing data from individuals with diabetes and healthy controls [34]**.** In boxplots (**b**, **d**, **e**) the centrelines show the medians; box limits indicate the 25th and 75th percentiles; whiskers extend from the hinge to the most extreme value no further than 1.5 × the IQR (i.e. the distance between the first and third quartiles). CD, collecting duct; CT, connecting tubule; CTRL, control; DCT, distal convoluted tubule; DM, diabetes mellitus; ENDO, endothelium; FC, fold change; FPKM, fragments per kilobase of transcript per million mapped fragments; glom, glomerular; HTN, hypertension; IC, intercalated cell (A/B); LEUK, leucocyte; LOH, loop of Henle; MES, mesangial cells; PC, principal cell; PCT, proximal convoluted tubule; PEC, parietal epithelial cells; PODO, podocytes; tub, tubular

Gene-level analysis identified ten genes associated with DKD. The *DCLK1* gene encodes a doublecortin-like kinase. The histone modification-based ChromHMM 15-state model for fetal kidney indicated strong transcription overlapping one of the three lead SNPs in the *DCLK1* locus (rs61948262), and ChIP-seq data supported *ZSCAN4* binding to the locus in the HEK293 kidney epithelial cell line. In addition, the lead SNPs were kidney mQTLs for *DCLK1* CpG sites (*p*=6.8×10^–22^). Furthermore, multiple lines of evidence highlight the importance of *DCLK1* in DKD. The correlation between tubular *DCLK1* expression and fibrosis was among the strongest correlations both in the nephrectomy samples (*p*=7.4×10^−16^; Fig. 7) and in the Pima Indian biopsies (*p*=3.0×10^−4^), and glomerular *DCLK1* expression was nominally associated with glomerular width, mesangial volume and podocyte foot process width in the Pima Indian biopsies (*p*<0.05; ESM Table 13). Furthermore, both glomerular and tubular *DCLK1* expression were elevated in DKD in two additional datasets (fold change 1.98, *p*=1.2×10^−4^ for glomeruli [35]; fold change 2.09, *p*=0.003 for tubules [36]; Fig. 7). Finally, we previously identified a subset of transcripts, including *DCLK1*, targeted by the early growth response-1 transcription factor in a murine model of DKD. In that study, *Dclk1* expression was upregulated in diabetic vs non-diabetic *Apoe*^−/−^ mouse kidneys [37]. Taken together, these expression data in human and experimental DKD identify *DCLK1* as a novel target.

Kidney eQTL data for the top SNPs in the *INIP–SNX30* locus pointed towards *SNX30*, encoding the sorting nexin family member 30, with the DKD risk-associated rs786959 A allele associated with higher *SNX30* expression (*p*=4.6×10^−7^). On the contrary, in our transcriptomics data higher tubular *SNX30* was correlated with higher eGFR (*p*=5.8×10^−14^) and lower level of fibrosis (*p*<2.0×10^−16^); glomerular expression was correlated with less glomerulosclerosis (*p*=8.0×10^−5^). Finally, kidney *SNX30* expression was associated with higher eGFR in the general population using TWAS based on kidney tubular eQTL [17] and GWAS on eGFR [31] (*p*=0.046; ESM Table 8).

The TWAS analysis based on our GWAS results, integrated with micro-dissected tubular and glomerular eQTL data, predicted that *AKIRIN2* gene expression is elevated in tubules in individuals with severe DKD compared with individuals with normal AER (*p*=1.1×10^-6^). *AKIRIN2* gene expression was highly correlated with the level of fibrosis (*p*=2.8×10^−7^). *AKIRIN2* encodes a conserved nuclear factor that is a downstream effector of the toll-like receptor, TNF and IL-1β signalling pathways, involved in stimulating proinflammatory pathways [38]. This factor binds to nuclear NF-κB complexes and is required for the transcription of a subset of NF-κB-dependent genes such as *IL6*, *CXCL10* and *CCL5* [39]; NF-κB activation drives inflammatory responses and is activated in DKD [40].

The strongest regulatory evidence in RegulomeDB was obtained for rs1260634 intronic in the *LSM14A* gene: rs1260634 exerts strong transcription in 125 tissues including fetal kidney chromatin state model, is located in a *ZNF362* binding sequence in HEK293 cell line, and affects a predicted transcription factor binding motif for Kruppel-like factors 4 and 12 (KLF4 and KLF12) and Sp8 transcription factor (ESM Fig. 11). Furthermore, in our kidney mQTL data, rs1260634 showed strong association (*p*=2.1×10^−28^) with cg14143166, where methylation in blood was associated with DKD in our EWAS data (*p*=0.03). Tubular *LSM14A* expression correlated with higher eGFR (*p*=2.9×10^−6^), and glomerular expression with the decrease in mesangial volume (*p*=6.5×10^−4^; significant after correction for 29 tested genes and two tissues, but not for 27 phenotypes). *LSM14A* encodes an Sm-like protein, thought to participate in pre-mRNA splicing, and implicated in innate antiviral responses [41].

Other noteworthy novel genes include *EIF4E* and *PTPRN*. *EIF4E* encodes a common mRNA translation initiation factor; its activation and/or suppression are influenced by mTOR signalling cascades involved in DKD [42] as well as high glucose and high insulin environments in renal epithelial cells [43]. *PTPRN* encodes islet antigen 2 (IA-2), a major type 1 diabetes autoantigen involved in glucose-stimulated insulin secretion [44]. In mice, IA-2 is required to maintain normal levels of renin expression in kidneys [45]. Finally, the *MFF* gene identified in our gene-level analysis has been previously related to DKD [46]. However, the association may be driven by the neighbouring *COL4A3* association, as suggested previously [6].

Indeed, one limitation of our gene-level analysis is the inability to confidently assign genes to a given set of correlated SNPs within a region. While it is reasonable to prioritise the gene in which the SNPs lie, it remains possible that extended LD patterns are tagging other nearby genes. Similarly, assigning a causal gene for the lead SNPs is not straightforward. We have utilised the eQTL and other data when available but also used a simple nearest-gene approach to name the associated region.

In the transcriptomics analyses, as expected, all 13 significant correlations with the level of fibrosis were observed for tubular gene expression, and the two observed correlations for glomerulosclerosis were for glomerular expression of *SNX30* and *COLEC11* (Fig. 4). Interestingly, eight out of ten correlations with eGFR were obtained for tubular rather than glomerular gene expression, supporting the importance of tubular damage in the loss of renal function.

While there is a strong epidemiological link between DKD and coronary artery disease in diabetes [1], our LDSR is the first study to report a genetic correlation between these major diabetic complications. Among the lipid traits, significant correlation with DKD was found only for lower HDL-cholesterol, despite previous MR of kidney disease in the general population implicating HDL as a marker of dyslipidaemia rather than a causal factor [47]. Indeed, our subsequent MR found no evidence of causality between HDL and DKD; in concordance with our previous MR on BMI [48], only obesity-related traits were causal risk factors for DKD. However, we cannot exclude that the associations detected in our studies might partly reflect collider bias. Of note, our current MR was in line with our previous MR in type 1 diabetes suggesting that serum urate levels are not a causal risk factor for DKD [49]; similar negative results were also reported for non-diabetic CKD [50].

Most kidney disease in individuals with type 1 diabetes is considered to occur due to diabetic nephropathy, histologically characterised by thickening of the glomerular basement membrane and mesangial expansion, as well as renal tubular, interstitial and arteriolar lesions. In individuals with type 2 diabetes, only a proportion of DKD is purely due to diabetic nephropathy, whereas ageing, obesity and hypertension also contribute to kidney decline. These differences were also seen in our genetic correlation analysis, with CKD in type 2 diabetes genetically resembling CKD and eGFR in the general population, and no significant correlation observed in individuals with type 1 diabetes. Thus, including individuals with type 2 diabetes in the meta-analysis increases the heterogeneity of the underlying disease. However, as type 2 diabetes represents 95% of all diabetes cases, including those individuals increases statistical power for our current work and future GWAS meta-analyses integrating multiple subtypes of diabetes to identify shared genetic risk factors for DKD.

## Supplementary information

ESM 1 (PDF 8564 kb)

## Data Availability

The GWAS meta-analysis results can be accessed via the type 1 and type 2 diabetes (T1D and T2D, respectively) and Common Metabolic Diseases (CMD) Knowledge Portals, and downloaded on their respective download pages (https://t1d.hugeamp.org/downloads.html; https://t2d.hugeamp.org/downloads.html; https://hugeamp.org/downloads.html).

## Abbreviations

ACR: Albumin/creatinine ratio
CKD: Chronic kidney disease
DKD: Diabetic kidney disease
DNCRI: Diabetic Nephropathy Collaborative Research Initiative
eQTL: Expression quantitative trait locus
ESRD: End-stage renal disease
EWAS: Epigenome-wide association study
FinnDiane: Finnish Diabetic Nephropathy Study
GWAS: Genome-wide association studies
LD: Linkage disequilibrium
LDSR: Linkage disequilibrium score regression
MAF: Minor allele frequency
mGFR: Measured GFR
mQTL: Methylation quantitative trait locus
MR: Mendelian randomisation
PoPS: Polygenic Priority Score
SUMMIT: SUrrogate markers for Micro- and Macrovascular hard endpoints for Innovative diabetes Tools
SUMMIT-1: SUMMIT type 1 diabetes studies
SUMMIT-2: SUMMIT type 2 diabetes studies
TWAS: Transcriptome-wide association study
UK-ROI: All Ireland-Warren 3-Genetics of Kidneys in Diabetes (GoKinD) United Kingdom
λ_GC_: λ genomic control inflation factor
