# Supplementary material for: Genome-wide meta-analysis and omics integration identifies novel genes associated with diabetic kidney disease
Source: Diabetologia. 2022 Jun 28;65(9):1495–509. doi: 10.1007/s00125-022-05735-0 (PMC9345823; doi:10.1007/s00125-022-05735-0)

Genome-wide meta-analysis and omics integration identifies novel genes associated with diabetic kidney disease

Niina Sandholm*, Joanne B Cole*, Viji Nair, Xin Sheng, Hongbo Liu, Emma Ahlqvist, Natalie van Zuydam, Emma H Dahlström, Damian Fermin, Laura J Smyth, Rany M Salem, Carol Forsblom, Erkka Valo, Valma Harjutsalo, Eoin P Brennan, Gareth J McKay, Darrell Andrews, Ross Doyle, Helen C Looker, Robert G Nelson, Colin Palmer, Amy Jayne McKnight, Catherine Godson, Alexander P Maxwell, Leif Groop, Mark I McCarthy, Matthias Kretzler, Katalin Susztak, Joel N Hirschhorn, Jose C Florez, Per-Henrik Groop, for the GENIE Consortium.

Electronic Supplemental Material (ESM)

Contents

ESM METHODS 3

ESM References 11

ESM Table 1: A total of ten case – control definitions for diabetic kidney disease (DKD; used as a general term to describe renal complications in diabetes) 17

ESM Table 2: Number of individuals in each cohort for each phenotypic comparison 18

ESM Table 3: Key characteristics of the genotyping and statistical analyses in DNCRI and SUMMIT cohorts. 19

ESM Table 4: List of related traits studied with LD score regression. 20

ESM Table 5: Association details for the novel genome-wide significant locus rs72831309 in *TENM2* ... 22

ESM Table 6: Highly correlated reconstituted gene-sets that make-up the “basement membrane” meta-gene set derived in Marouli *et al* 23

ESM Table 7: Association results for the lead SNPs at the MAGMA/PASCAL genes. 24

ESM Table 8: TWAS results with $p<1\times10^{-4}$. 25

ESM Table 9: eQTL and association data for SNPs that were selected by elastic net regression model in TWAS contributing to *AKIRIN2* TWAS association 26

ESM Table 10: Kidney eQTL associations with $p<1\times10^{-4}$ in tubular or glomerular eQTL data, or in the kidney eQTL meta-analysis for the lead SNPs. 27

ESM Table 11: Significant kidney mQTL associations ($p<1.46\times10^{-11}$ Bonferroni-adjusted genome-wide significance; 1×10^{-7} suggestive significance) for lead loci. 28

ESM Table 12: Correlation between glomerular and tubular gene expression and glomerulosclerosis, fibrosis, and eGFR in nephrectomy samples. 29

ESM Table 13: Gene centric summary of the lead genes 30

ESM Table 14: Mendelian Randomization (MR) results for DKD (All vs Ctrl) 32

ESM Table 15. Egger intercepts for Mendelian Randomization analyses on DKD (all vs. Ctrl phenotype). 33

ESM Figure 1: Odds ratios required to detect association with ≥ 80% statistical power, in function of the variant minor allele frequency [MAF]. 34

ESM Figure 2: Manhattan and QQ-plots of the ten DKD GWAS meta-analysis results. 35

ESM Figure 3: Regional association plot for the *COL4A3* gene region associated with Severe DKD, indicating a secondary association peak at chr2:228259302 (rs6436688, effect allele (A) frequency 56%, OR = 1.13 (95% confidence interval 1.08 – 1.19), p-value 1.79×10^{-7}). 38

ESM Figure 4: Regional association plots for the GWAS meta-analysis lead loci (A-K). 39

A: CKD+DKD chr5:166978230 (rs72831309)....................39
B: CKD chr2:3745215 (rs12615970)....................39
C: Severe DKD chr2:228121101 (rs55703767)....................40
D: ESRD chr3:926345 (rs115061173)....................40
F: ESRD vs. All chr3:36566312 (rs116216059)....................42
G: Severe DKD chr4:71358776 (rs191449639)....................43
H: Micro chr7:99728546 (rs77273076)....................43
I: ESRD vs. macro chr8:128100029 (rs551191707)....................44
J: Micro chr11:16937846 (rs183937294)....................44
K: CKD chr18:1811108 (rs185299109)....................45

ESM Figure 5: Gene prioritization for the COL4A3 gene at lead SNP rs5570367 associated with Severe DKD using multiple intersecting gene prioritization approaches (PoPS, nearest gene, and MAGMA).....46

ESM Figure 6: Regional association plots for the gene-level analysis results from MAGMA and PASCAL analysis....................47

A: CKD, *PTPRN*....................47
B: CKD, *RESP18*....................47
C: Severe DKD, *MFF*....................48
D: ESRD vs. macro, *EIF4E*....................48
E: DKD, *INIP*....................49
F: DKD, *SNX30*....................49
G: Severe DKD, *GPR158*....................50
H: ESRD vs. macro, *DCLK1*....................50
I: Severe DKD, *LSM14A*....................51
J: CKD extremes, COL20A1....................51

ESM Figure 7: TWAS QQ-plots and genomic control λGC inflation factors for A) tubular and B) glomerular expression....................52

ESM Figure 8: Tubular and glomerular gene expression of the lead genes correlate with multiple morphological and pathological renal parameters....................54

ESM Figure 9: Genetic correlation between DKD and related traits based on LD score regression.........55

ESM Figure 10: Mendelian Randomization scatter plots for SNP effects for the metabolic traits vs. DKD (All vs. Ctrl)....................56

ESM Figure 11: rs1260634 intronic in the *LSM14A* gene affects the predicted binding motifs for KLF12, KLF4, and SP8 (top to bottom)....................57

ESM METHODS

Participating studies and phenotype definitions: A total of ten case – control definitions for diabetic kidney disease (DKD) were included in DNCRI [1], based on either urinary albumin excretion rate (AER; divided into controls with normal AER, and cases with microalbuminuria, macroalbuminuria, or ESRD), estimated glomerular filtration rate (eGFR), or both, and harmonized to match and include all seven phenotypic definitions assessed in The SUrrogate markers for Micro- and Macrovascular hard endpoints for Innovative diabetes Tools consortium (SUMMIT) type 1 diabetes (SUMMIT-1) analyses [2] and SUMMIT type 2 diabetes (SUMMIT-2) analyses [3] (**ESM Table 1**). All individuals (both cases and controls) had diabetes (either type 1 or type 2 diabetes) and cases had some form of kidney disease. The phenotypic comparisons are as follows: controls with normal AER vs. DKD cases with microalbuminuria or worse ("All vs. Ctrl"), macroalbuminuria or worse ("Severe DKD"), microalbuminuria alone ("Micro"), or "ESRD"; ESRD cases vs. everyone else ("ESRD vs. All"); controls with normal eGFR defined as eGFR $\geq$ 60 ml/min/1.73 m^2 vs. CKD defined as eGFR < 60 ml/min/1.73 m^2 ("CKD"); and "CKD-DKD" based on both AER and eGFR, with controls with normal AER and eGFR vs. cases with microalbuminuria or worse and eGFR < 45 ml/min/1.73 m^2. For the three phenotypic comparisons not initially part of the SUMMIT analysis (normal AER vs. macroalbuminuria ["Macro"], ESRD vs. macroalbuminuria ["ESRD vs. macro"], and controls with eGFR $\geq$ 60 ml/min/1.73m^2 vs. CKD cases with eGFR < 15 ml/min/1.73m^2 or ESRD ["CKD extremes"]), GWAS and meta-analysis were performed with three SUMMIT type 2 diabetes studies (Genetics of Diabetes Audit and Research, Tayside and Scotland [GoDARTS] 1 and 2, and Scania Diabetes Registry [SDR] type 2 diabetes cohort) and the SDR type 1 diabetes cohort. Individuals from the Finnish Diabetic Nephropathy Study (FinnDiane) were included in both the original DNCRI (N=6,019) and SUMMIT-1 analyses (N=3,415), but for the purpose of this meta-analysis, FinnDiane was only included in the DNCRI meta-analysis and therefore excluded from the SUMMIT-1 data (**ESM Table 2**). All contributing studies were performed in accordance with the Declaration of Helsinki and Declaration of Istanbul.

Genome-wide association study (GWAS) and meta-analysis: Genotyping and statistical analysis of the DNCRI [1] and SUMMIT [2, 3] cohorts have been previously described, and the statistical analyses were limited to the previously published results (apart from the three additional phenotypes for a subset of SUMMIT

cohorts mentioned above). Analysis plans were similar in both consortia, and the main characteristics are described in **ESM Table 3**. Imputation was performed using 1000Genomes Phase 3 reference panel in DNCRI, and the older 1000Genomes phase 1 panel in the SUMMIT cohorts. In both consortia, analyses were performed in unrelated individuals using the SNPtest additive score test, adjusting for age, sex, diabetes duration, the genetic principal components, and study-specific covariates (e.g., site or genotyping batch). Variants were filtered for INFO imputation quality score ≥ 0.3 (DNCRI) or ≥ 0.4 (SUMMIT) and minor allele count ≥ 10 in both cases and in controls. In SUMMIT, variants were further filtered to those with minor allele frequency (MAF) ≥ 0.01. Within-consortium meta-analyses were performed with inverse-variance fixed effects meta-analysis based on the effect size estimates. Meta-analyses between DNCRI, SUMMIT-1, and SUMMIT-2 were performed with inverse-variance fixed effect methods based on the effect size estimates from the summary statistics for each of the three datasets with METAL software (released 2011-03-25) [4]. Finally, variants were limited to those found in at least two studies and reported in the 1000Genomes phase 3 reference panel. In addition, we performed meta-analyses for each DKD trait separately for individuals with type 1 diabetes (DNCRI and SUMMIT-1) and type 2 diabetes (SUMMIT-2), and calculated heterogeneity between type 1 and type 2 diabetes studies with METAL software [4]. Study-wise summary statistics were available for DNCRI and SUMMIT-1 studies. Regional association plots were plotted with LocusZoom [5].

We estimated the significance level for multiple testing correction using a method suggested previously [6]:correction for multiple testing was estimated with spectral decomposition of the GWAS Z-scores of the non-missing variants across the ten DKD traits, which suggested 5.36 effective tests, leading to a corrected significance threshold of $p<9.3\times10^{-9}$.

The FinnDiane GWAS data, originally imputed together with the other DNCRI GWAS data sets using 1000Genomes Phase 3 reference panel, was re-imputed with a Finnish population-specific SISu v3 imputation reference panel. Samples were pre-phased with Eagle 2.3.5 [7], and genotype imputation was performed with Beagle 4.1 (version 08Jun17.d8b) [8].

We performed conditional analysis of the *COL4A3* locus with apparent secondary association peak using GCTA v1.93β [9] and FinnDiane GWAS data as the reference panel.

Gene-prioritization analysis: Gene prioritization at each of our top loci was performed using two complementary similarity-based gene prioritization approaches (PoPS v0.1 [10] and MAGMA v1.06b [11]), which integrate GWAS summary statistics with gene set enrichment analysis based on a variety of biological annotation datasets including gene expression, curated pathways, protein-protein interactions, and mouse gene knock-out studies.

For PoPS gene prioritization, MAGMA is first used to calculate gene-level association statistics for 18 383 protein-coding genes in the genome, which is used to assess feature enrichment. PoPS then calculates polygenic priority (PoP) scores for each gene based on its membership to enriched features. For each of our top loci, we annotated the PoPS prioritized gene as the one with the highest PoP score within a 500kb flanking window of each of our lead SNPs. Of note, the *PRNCR1* gene annotated as the nearest gene to SNP rs185299109 was not included in the PoPS protein-coding gene dataset and the CKD-associated SNP rs185299109 located in an intergenic region was also excluded from this analysis.

MAGMA gene prioritization was conducted using a recently developed extension to the method as described and implemented in Benchmarker software [12], enabling the explicit derivation of gene prioritization results from gene set enrichment analysis. Like the Benchmarker approach, we classified genes as members of each gene set using the top 50, 100, and 200 ranked genes, and obtained similar results from all three. To identify the PoPS features that contributed to the prioritization of *COL4A3*, we limited it to the selected marginal gene features (PoPS step 1), multiplied the *COL4A3* beta hats (PoPS step 2) by the *COL4A3* feature's scores, and ranked the features by the highest overall score.

Gene-level analysis: SNP summary statistics from the GWAS meta-analysis were aggregated by gene-level regression analysis using two related software programs designed for gene-level analysis, MAGMA v1.06b [11] and PASCAL v2016 [13], using default parameters. Both methods take into account pairwise SNP correlation within a defined gene region (MAGMA 5kb flanking; PASCAL 50kb flanking) to calculate gene-level scores while accounting for linkage disquilibirium. Gene-level significance thresholds were determined by a Bonferroni multiple-testing correction based on the number of genes tested for each of the ten phenotypes

within each software program (number of genes ranged from 18,439-21,790; significance thresholds ranged from 2.7×10^{-6} to 2.3×10^{-6}).

Transcriptome-wide association study (TWAS): MetaXcan [14] was applied to integrate kidney expression quantitative trait locus (eQTL) datasets with the GWAS meta-analysis results and to map disease-associated genes. The *cis*-eQTL data for microdissected human glomerular (N=119) and tubular (N=121) samples were obtained from Susztaklab Kidney Biobank (https://susztaklab.com/eQTLci/download.php) [15], and were analyzed jointly to infer differential gene expression in cases vs. controls using MetaXcan software with default parameters. The GTEx Elastic-Net Model pipeline (https://github.com/hakyimlab/PredictDBPipeline) was applied to prepare the model used for MetaXcan. The linkage disequilibrium (LD) references were estimated based on genotypes of European individuals from the 1000 Genome Project. Using FDR < 0.05, the method identified 5,990 coding genes with significant models for glomerular eQTL, and 5,371 coding genes for tubular eQTL. Significant association was defined as $p<0.05/2/6{,}050=4.1\times10^{-6}$, i.e., corrected for two tissues and 6,050 genes found in either tubular or glomerular eQTL data.

Expression quantitative trait loci (eQTL): eQTL associations were sought from the eQTLGen database for eQTL in whole blood from >30 000 participants (http://www.eqtlgen.org/) [16]. Kidney specific eQTL associations were queried from eQTL datasets for glomeruli, tubules [15], and a meta-analysis of four eQTL studies with 451 kidney samples. The meta-analysis of four eQTLs datasets obtained from the Susztak lab, The Cancer Genome Atlas (TCGA), the Genotype-Tissue Expression (GTEx v8), and the Nephrotic Syndrome Study Network (NephQTL) [15, 17–19], was performed using METAL with fixed effects inverse-variance meta-analysis[4].

Methylation quantitative trait loci (mQTL): mQTL associations were sought for the lead SNPs in 188 healthy kidney samples (eGFR > 60 and fibrosis < 10%), with Bonferroni threshold ($p<1.5\times10^{-11}$) considered genome-wide significant. DNA methylation of CpG sites were profiled in 188 healthy kidney samples by the Infinium MethylationEPIC Kit and BeadChips (Illumina, USA) and were transformed by an inverse-normal

transformation after quality control using SeSAMe [20]. Genotypes for these samples were profiled by from Axiom Tx and Axiom Biobank arrays and imputed using the multiethnic panel reference from 1000Genomes Phase 3 (NCBI build 37, released in October 2014). The association between CpG site and the SNPs within 1Mb were estimated by linear regression model using MatrixQTL [21]. with covariates including collection site, age, sex, top five genotype principal components (PCs), degree of bisulfite conversion, sample plate, and sentrix position and PEER factors. For the significant CpG sites, we then sought for evidence of association between blood methylation levels and eGFR, or eGFR decline, in 500 individuals with diabetes [22]; we furthermore tested association with DKD in our epigenome-wide association study in 1,304 UK-ROI and FinnDiane participants, analyzed using the Infinium MethylationEPIC Kit and BeadChips (Illumina, USA), following the QC and analysis procedures described earlier for UK-ROI[23]. Meta-analysis of the two data sets was performed with METAL software [4], based on *p*-values and direction of effect.

Multiple trait co-localization (moloc): To estimate posterior probability that the GWAS lead variant is associated with kidney eQTL (kidney eQTL meta-analysis of N=686 samples, https://susztaklab.com/Kidney_eQTL/eQTLmeta.php) and mQTL (kidney samples described above, N=188) signals, we performed Bayesian multiple-trait-colocalization (moloc). Lead variants were determined as the variants with p-value < 1×10^{-4} within the lead loci identified from the GWAS meta-analysis or MAGMA/PASCAL gene aggregate tests, and available variants within 100kb search window were extracted for moloc analysis. R package moloc (v0.1.0) [24] was used to perform moloc analysis with default parameters prior_var = c(0.01, 0.1, 0.5) and priors = c(1×10^{-4}, 1×10^{-6}, 1×10^{-7}). In moloc results, Coloc_ppas_abc > 0.8 was considered evidence of colocalization among all three traits. Coloc_ppas_ab > 0.8 was considered evidence of colocalization between GWAS and mQTL. Coloc_ppas_ac > 0.8 was considered evidence of colocalization between GWAS and eQTL.

Human kidney gene expression: For the 29 lead genes or transcripts underlying or located near the lead SNPs, or based on gene-level analyses, TWAS, PoPS, or kidney eQTL data, we studied gene expression in kidneys in human transcriptomics data from nephrectomy samples (433 tubule and 335 glomerulus samples)

[25] and kidney biopsies from the Pima Indian cohort (67 glomerular and 47 tubulointerstitial tissues) [26], and tested for correlation with relevant pathological phenotypes. The microdissected nephrectomy samples were from individuals with varying degree of diabetic and hypertensive kidney disease, and gene expression was defined with RNA sequencing. Pearson correlation *p*-values below 2.2×10^{-4} were considered significant, corrected for multiple testing for 29 genes, two tissue compartments, and four phenotypes (eGFR, fibrosis, glomerulosclerosis and group comparison). The study was approved by the institutional review board of the University of Pennsylvania.

In the Pima Indian cohort, gene expression profiling in the first biopsy was performed with Affymetrix gene chip arrays [26], and with Illumina RNA-sequencing for the second biopsy, as described earlier [1]. Available phenotypes included progression to ESRD, measured GFR (mGFR), albumin-to-creatinine ratio (ACR), glycated hemoglobin (HbA1c) and six kidney morphological parameters for both biopsies, and change in the phenotypes between the first and the second study biopsies (27 phenotypes in total) [27]. Pearson correlation *p*-values below 3.2×10^{-5}were considered significant, corrected for 29 genes, 2 tissues, and 27 phenotypes; *p*-values below 8.6×10^{-4} (i.e. without correction for 27 phenotypes) were considered suggestive. The study was approved by the Institutional Review Board of the National Institute of Diabetes and Digestive and Kidney Diseases.

Further annotation of the lead variants: Chromatin 3D conformation interactions with gene transcription start sites (TSS) were queried for the most significant SNPs from the promoter capture Hi-C (PCHiC) data from the www.chicp.org web interface, including data for GM12878 lymphoblastoid cell line and CD34 cells [28], hESC derived cardiomyocytes [29], 16 primary blood cells [30], and pancreatic islets [31]; no data was available for kidney tissue. Interactions with score ≥ 5 were considered significant. We queried chromatin accessibility in kidney single-nucleus ATAC-sequencing (snATACseq) data available at https://susztaklab.com/human_kidney/igv/ (accessed 24 June 2021) [32]. Detailed gene expression in kidney single cell RNA sequencing (scRNAseq) data was queried in the Human Diabetic Kidney data set (23,980 nuclei) by Wilson *et al*. [33], accessed through http://humphreyslab.com/SingleCell. Further epigenetic annotation was sought from the regulomeDB [34], and differential renal gene expression in DKD versus healthy controls from the Ju CKD [35] and Woroniecka [36] data sets in the NephroSeq portal

(www.nephroseq.org). Of note, samples in the queried Woroniecka [36] data are a subset (N=22) of the more recent RNA sequencing based nephrectomy samples mentioned above (N=433) [25].

Genetic correlation of DKD between type 1 and type 2 diabetes and general population kidney traits: We calculated the genetic correlation between the DKD traits in type 1 diabetes studies versus type 2 diabetes studies using LD score regression (LDSR) [37] with LDSC v1.0.1 software (https://github.com/bulik/ldsc) using the European 1000 genomes LD structure and limited to the HapMap3 SNPs. Valid (non-significant) estimates were obtained only for the two phenotypes with the largest number of participants, DKD (N=26,989) and CKD (N=26,626). We further calculated genetic correlation for the full meta-analysis (type 1 or type 2 diabetes), and separately for the type 1 diabetes and type 2 diabetes meta-analysis results with the following general population kidney traits obtained from the CKDgen consortium: general population microalbuminuria (trans-ethnic, N=348,954 (51,861 cases, 297,093 controls) [38]; albumin-to-creatinine-ratio [ACR] (European-American, N=547361) [38]; ACR in diabetes (trans-ethnic, N=51,541) [38]; CKD (European Americans: N=480,698 (41,395 cases and 439,303 controls) [39]; and eGFR (CKDGen, N=765,348, trans-ethnic, and UK Biobank, N=436,581, Europeans) [40]. Estimates of genetic correlation were obtained for 5 out of the 10 studied DKD phenotypes. Correlation plots were plotted with heatmap.2 function from the R gplots package (v.3.1.1, https://cran.r-project.org/web/packages/gplots/index.html).

LDSR and Mendelian Randomization (MR) of cardiometabolic and other related traits: LDSR [37] was performed at LDhub (http://ldsc.broadinstitute.org/) for 78 glycemic, autoimmune, anthropometric, bone, smoking behavior, lipid, kidney, uric acid, cardiometabolic, and aging related traits (listed in **ESM Table 15**), based on the GWAS summary statistics of the ten DKD phenotypes explored. Variants were filtered to those with MAF ≥1%. LDSR associations with $p<6.4\times10^{-4}$ were defined significant after Bonferroni correction for 78 traits. To identify causal relationships for significant traits in the LDSR against DKD, we performed summary-based two-sample MR implemented in the R package TwoSampleMR v0.5.6 [41]. For the SNP-trait associations, we selected genetic variants as instrumental variables (IV) that were independently associated with the selected traits ($p<5\times10^{-8}$; $r^2 < 0.001$ based on the 1000Genomes EUR panel; LD window=10,000 kb) from published GWAS. Palindromic SNPs with intermediate allele frequency (MAF close to 50%) were removed. Traits with less than five IVs were excluded from the MR analysis. Primarily,

we used inverse variance-weighted (IVW) regression, but causality was further assessed using methods less sensitive to pleiotropy/heterogeneity (weighted median and MR-Egger regression) [42]. Heterogeneity of SNP estimates in MR was assessed with Cochran's Q statistic *p*-value and the I^2 statistic. The MR–Egger intercept test was used to detect unbalanced horizontal pleiotropy. As all but one significant LDSR associations were for the "All vs. DKD" phenotype with the largest number of included participants, only those results are shown in Figure 6; also association between "Obesity Class I" vs. CKD was tested and significant.

ESM References

1. Salem RM, Todd JN, Sandholm N, et al (2019) Genome-Wide Association Study of Diabetic Kidney Disease Highlights Biology Involved in Glomerular Basement Membrane Collagen. J Am Soc Nephrol 30(10):2000–2016. https://doi.org/10.1681/ASN.2019030218

2. Sandholm N, Van Zuydam N, Ahlqvist E, et al (2017) The Genetic Landscape of Renal Complications in Type 1 Diabetes. J Am Soc Nephrol 28(2):557–574. https://doi.org/10.1681/ASN.2016020231

3. van Zuydam NR, Ahlqvist E, Sandholm N, et al (2018) A Genome-Wide Association Study of Diabetic Kidney Disease in Subjects With Type 2 Diabetes. Diabetes 67(7):1414–1427. https://doi.org/10.2337/db17-0914

4. Willer CJ, Li Y, Abecasis GR (2010) METAL: fast and efficient meta-analysis of genomewide association scans. Bioinformatics 26(17):2190–2191. https://doi.org/10.1093/bioinformatics/btq340

5. Pruim RJ, Welch RP, Sanna S, et al (2010) LocusZoom: regional visualization of genome-wide association scan results. Bioinformatics 26(18):2336–2337. https://doi.org/10.1093/bioinformatics/btq419;

6. Li J, Ji L (2005) Adjusting multiple testing in multilocus analyses using the eigenvalues of a correlation matrix. Heredity (Edinb) 95(3):221–227. https://doi.org/10.1038/sj.hdy.6800717

7. Loh P-R, Palamara PF, Price AL (2016) Fast and accurate long-range phasing in a UK Biobank cohort. Nat Genet 48(7):811–816. https://doi.org/10.1038/ng.3571

8. Browning BL, Browning SR (2016) Genotype Imputation with Millions of Reference Samples. Am J Hum Genet 98(1):116–126. https://doi.org/10.1016/j.ajhg.2015.11.020

9. Yang J, Ferreira T, Morris AP, et al (2012) Conditional and joint multiple-SNP analysis of GWAS summary statistics identifies additional variants influencing complex traits. Nat Genet 44(4):369–375, S1-3. https://doi.org/10.1038/ng.2213

10. Weeks EM, Ulirsch JC, Cheng NY, et al (2020) Leveraging polygenic enrichments of gene features to predict genes underlying complex traits and diseases. medRxiv 2020.09.08.20190561. https://doi.org/10.1101/2020.09.08.20190561

11. de Leeuw CA, Mooij JM, Heskes T, Posthuma D (2015) MAGMA: generalized gene-set analysis of GWAS data. PLoS ComputBiol 11(4):e1004219. https://doi.org/10.1371/journal.pcbi.1004219

12. Fine RS, Pers TH, Amariuta T, Raychaudhuri S, Hirschhorn JN (2019) Benchmarker: An Unbiased, Association-Data-Driven Strategy to Evaluate Gene Prioritization Algorithms. Am J Hum Genet 104(6):1025–1039. https://doi.org/10.1016/j.ajhg.2019.03.027

13. Lamparter D, Marbach D, Rueedi R, Kutalik Z, Bergmann S (2016) Fast and Rigorous Computation of Gene and Pathway Scores from SNP-Based Summary Statistics. PLoS ComputBiol 12(1):e1004714. https://doi.org/10.1371/journal.pcbi.1004714

14. Barbeira AN, Dickinson SP, Bonazzola R, et al (2018) Exploring the phenotypic consequences of tissue specific gene expression variation inferred from GWAS summary statistics. Nat Commun 9(1):1825. https://doi.org/10.1038/s41467-018-03621-1

15. Qiu C, Huang S, Park J, et al (2018) Renal compartment-specific genetic variation analyses identify new pathways in chronic kidney disease. NatMed. https://doi.org/10.1038/s41591-018-0194-4

16. Võsa U, Claringbould A, Westra H-J, et al (2018) Unraveling the polygenic architecture of complex traits using blood eQTL metaanalysis. bioRxiv (Journal Article):447367. https://doi.org/10.1101/447367

17. Ko Y-A, Yi H, Qiu C, et al (2017) Genetic-Variation-Driven Gene-Expression Changes Highlight Genes with Important Functions for Kidney Disease. Am J Hum Genet 100(6):940–953. https://doi.org/10.1016/j.ajhg.2017.05.004

18. GTEx Consortium (2020) The GTEx Consortium atlas of genetic regulatory effects across human tissues. Science 369(6509):1318–1330. https://doi.org/10.1126/science.aaz1776

19. Gillies CE, Putler R, Menon R, et al (2018) An eQTL Landscape of Kidney Tissue in Human Nephrotic Syndrome. Am J Hum Genet 103(2):232–244. https://doi.org/10.1016/j.ajhg.2018.07.004

20. Zhou W, Triche TJ, Laird PW, Shen H (2018) SeSAMe: reducing artifactual detection of DNA methylation by Infinium BeadChips in genomic deletions. Nucleic Acids Res 46(20):e123. https://doi.org/10.1093/nar/gky691

21. Shabalin AA (2012) Matrix eQTL: ultra fast eQTL analysis via large matrix operations. Bioinformatics 28(10):1353–1358. https://doi.org/10.1093/bioinformatics/bts163

22. Sheng X, Qiu C, Liu H, et al (2020) Systematic integrated analysis of genetic and epigenetic variation in diabetic kidney disease. PNAS 117(46):29013–29024. https://doi.org/10.1073/pnas.2005905117

23. Smyth LJ, Kilner J, Nair V, et al (2021) Assessment of differentially methylated loci in individuals with end-stage kidney disease attributed to diabetic kidney disease: an exploratory study. ClinEpigenetics 13(1):99-021-01081–x. https://doi.org/10.1186/s13148-021-01081-x

24. Giambartolomei C, Zhenli Liu J, Zhang W, et al (2018) A Bayesian framework for multiple trait colocalization from summary association statistics. Bioinformatics 34(15):2538–2545. https://doi.org/10.1093/bioinformatics/bty147

25. Guan Y, Liang X, Ma Z, et al (2021) A single genetic locus controls both expression of DPEP1/CHMP1A and kidney disease development via ferroptosis. Nat Commun 12(1):5078. https://doi.org/10.1038/s41467-021-25377-x

26. Nair V, Komorowsky CV, Weil EJ, et al (2018) A molecular morphometric approach to diabetic kidney disease can link structure to function and outcome. Kidney Int 93(2):439–449. https://doi.org/10.1016/j.kint.2017.08.013

27. Looker HC, Mauer M, Saulnier P-J, et al (2019) Changes in Albuminuria But Not GFR are Associated with Early Changes in Kidney Structure in Type 2 Diabetes. J Am Soc Nephrol 30(6):1049–1059. https://doi.org/10.1681/ASN.2018111166

28. Mifsud B, Tavares-Cadete F, Young AN, et al (2015) Mapping long-range promoter contacts in human cells with high-resolution capture Hi-C. NatGenet 47(6):598–606. https://doi.org/10.1038/ng.3286

29. Choy MK, Javierre BM, Williams SG, et al (2018) Promoter interactome of human embryonic stem cell-derived cardiomyocytes connects GWAS regions to cardiac gene networks. NatCommun 9(1):2526-018-04931–0. https://doi.org/10.1038/s41467-018-04931-0

30. Javierre BM, Burren OS, Wilder SP, et al (2016) Lineage-Specific Genome Architecture Links Enhancers and Non-coding Disease Variants to Target Gene Promoters. Cell 167(5):1369-1384.e19

31. Miguel-Escalada I, Bonàs-Guarch S, Cebola I, et al (2018) Human pancreatic islet 3D chromatin architecture provides insights into the genetics of type 2 diabetes. bioRxiv (Journal Article):400291

32. Sheng X, Ma Z, Wu J, et al (2020) Mapping the genetic architecture of human traits to cell types in the kidney identifies mechanisms of disease and potential treatments. bioRxiv 2020.11.09.375592. https://doi.org/10.1101/2020.11.09.375592

33. Wilson PC, Wu H, Kirita Y, et al (2019) The single-cell transcriptomic landscape of early human diabetic nephropathy. Proc Natl Acad Sci U S A 116(39):19619–19625. https://doi.org/10.1073/pnas.1908706116

34. Boyle AP, Hong EL, Hariharan M, et al (2012) Annotation of functional variation in personal genomes using RegulomeDB. Genome Res 22(9):1790–1797. https://doi.org/10.1101/gr.137323.112;

35. Ju W, Greene CS, Eichinger F, et al (2013) Defining cell-type specificity at the transcriptional level in human disease. Genome Res 23(11):1862–1873. https://doi.org/10.1101/gr.155697.113

36. Woroniecka KI, Park AS, Mohtat D, Thomas DB, Pullman JM, Susztak K (2011) Transcriptome analysis of human diabetic kidney disease. Diabetes 60(9):2354–2369. https://doi.org/10.2337/db10-1181

37. Bulik-Sullivan BK, Loh PR, Finucane HK, et al (2015) LD Score regression distinguishes confounding from polygenicity in genome-wide association studies. NatGenet 47(3):291–295. https://doi.org/10.1038/ng.3211

38. Teumer A, Li Y, Ghasemi S, et al (2019) Genome-wide association meta-analyses and fine-mapping elucidate pathways influencing albuminuria. Nat Commun 10(1):4130. https://doi.org/10.1038/s41467-019-11576-0

39. Wuttke M, Li Y, Li M, et al (2019) A catalog of genetic loci associated with kidney function from analyses of a million individuals. Nat Genet 51(6):957–972. https://doi.org/10.1038/s41588-019-0407-x

40. Stanzick KJ, Li Y, Schlosser P, et al (2021) Discovery and prioritization of variants and genes for kidney function in >1.2 million individuals. Nat Commun 12(1):4350. https://doi.org/10.1038/s41467-021-24491-0

41. Hemani G, Zheng J, Elsworth B, et al (2018) The MR-Base platform supports systematic causal inference across the human phenome. Elife 7:e34408. https://doi.org/10.7554/eLife.34408

42. Bowden J, Davey Smith G, Haycock PC, Burgess S (2016) Consistent Estimation in Mendelian Randomization with Some Invalid Instruments Using a Weighted Median Estimator. Genet Epidemiol 40(4):304–314. https://doi.org/10.1002/gepi.21965

43. Levey AS, Bosch JP, Lewis JB, Greene T, Rogers N, Roth D (1999) A more accurate method to estimate glomerular filtration rate from serum creatinine: a new prediction equation. Modification of Diet in Renal Disease Study Group. AnnInternMed 130(6):461–470

44. Levey AS, Stevens LA (2010) Estimating GFR using the CKD Epidemiology Collaboration (CKD-EPI) creatinine equation: more accurate GFR estimates, lower CKD prevalence estimates, and better risk predictions. AmJKidney Dis 55(4):622–627. https://doi.org/10.1053/j.ajkd.2010.02.337;

45. Levey AS, Coresh J, Greene T, et al (2007) Expressing the Modification of Diet in Renal Disease Study equation for estimating glomerular filtration rate with standardized serum creatinine values. ClinChem 53(4):766–772

46. Pilling LC, Atkins JL, Bowman K, et al (2016) Human longevity is influenced by many genetic variants: evidence from 75,000 UK Biobank participants. Aging (Albany NY) 8(3):547–560. https://doi.org/10.18632/aging.100930

47. Lango Allen H, Estrada K, Lettre G, et al (2010) Hundreds of variants clustered in genomic loci and biological pathways affect human height. Nature 467(7317):832–838. https://doi.org/10.1038/nature09410

48. Speliotes EK, Willer CJ, Berndt SI, et al (2010) Association analyses of 249,796 individuals reveal 18 new loci associated with body mass index. NatGenet 42(11):937–948. https://doi.org/10.1038/ng.686;

49. Bradfield JP, Taal HR, Timpson NJ, et al (2012) A genome-wide association meta-analysis identifies new childhood obesity loci. Nat Genet 44(5):526–531. https://doi.org/10.1038/ng.2247

50. Taal HR, Pourcain BS, Thiering E, et al (2012) Common variants at 12q15 and 12q24 are associated with infant head circumference. Nat Genet 44(5):532–538. https://doi.org/10.1038/ng.2238

51. Horikoshi M, Yaghootkar H, Mook-Kanamori DO, et al (2013) New loci associated with birth weight identify genetic links between intrauterine growth and adult height and metabolism. Nat Genet 45(1):76–82. https://doi.org/10.1038/ng.2477

52. Cousminer DL, Berry DJ, Timpson NJ, et al (2013) Genome-wide association and longitudinal analyses reveal genetic loci linking pubertal height growth, pubertal timing and childhood adiposity. Hum Mol Genet 22(13):2735–2747. https://doi.org/10.1093/hmg/ddt104

53. Berndt SI, Gustafsson S, Mägi R, et al (2013) Genome-wide meta-analysis identifies 11 new loci for anthropometric traits and provides insights into genetic architecture. Nat Genet 45(5):501–512. https://doi.org/10.1038/ng.2606

54. van der Valk RJP, Kreiner-Møller E, Kooijman MN, et al (2015) A novel common variant in DCST2 is associated with length in early life and height in adulthood. Hum Mol Genet 24(4):1155–1168. https://doi.org/10.1093/hmg/ddu510

55. Shungin D, Winkler TW, Croteau-Chonka DC, et al (2015) New genetic loci link adipose and insulin biology to body fat distribution. Nature 518(7538):187–196. https://doi.org/10.1038/nature14132

56. Chan Y, Salem RM, Hsu Y-HH, et al (2015) Genome-wide Analysis of Body Proportion Classifies Height-Associated Variants by Mechanism of Action and Implicates Genes Important for Skeletal Development. Am J Hum Genet 96(5):695–708. https://doi.org/10.1016/j.ajhg.2015.02.018

57. Lu Y, Day FR, Gustafsson S, et al (2016) New loci for body fat percentage reveal link between adiposity and cardiometabolic disease risk. Nat Commun 7:10495. https://doi.org/10.1038/ncomms10495

58. Horikoshi M, Beaumont RN, Day FR, et al (2016) Genome-wide associations for birth weight and correlations with adult disease. Nature 538(7624):248–252. https://doi.org/10.1038/nature19806

59. Warrington NM, Beaumont RN, Horikoshi M, et al (2019) Maternal and fetal genetic effects on birth weight and their relevance to cardio-metabolic risk factors. Nat Genet 51(5):804–814. https://doi.org/10.1038/s41588-019-0403-1

60. Moffatt MF, Kabesch M, Liang L, et al (2007) Genetic variants regulating ORMDL3 expression contribute to the risk of childhood asthma. Nature 448(7152):470–473. https://doi.org/10.1038/nature06014

61. Dubois PCA, Trynka G, Franke L, et al (2010) Multiple common variants for celiac disease influencing immune gene expression. Nat Genet 42(4):295–302. https://doi.org/10.1038/ng.543

62. International Multiple Sclerosis Genetics Consortium, Wellcome Trust Case Control Consortium 2, Sawcer S, et al (2011) Genetic risk and a primary role for cell-mediated immune mechanisms in multiple sclerosis. Nature 476(7359):214–219. https://doi.org/10.1038/nature10251

63. Okada Y, Wu D, Trynka G, et al (2014) Genetics of rheumatoid arthritis contributes to biology and drug discovery. Nature 506(7488):376–381. https://doi.org/10.1038/nature12873

64. Liu JZ, van Sommeren S, Huang H, et al (2015) Association analyses identify 38 susceptibility loci for inflammatory bowel disease and highlight shared genetic risk across populations. Nat Genet 47(9):979–986. https://doi.org/10.1038/ng.3359

65. Cordell HJ, Han Y, Mells GF, et al (2015) International genome-wide meta-analysis identifies new primary biliary cirrhosis risk loci and targetable pathogenic pathways. Nat Commun 6:8019. https://doi.org/10.1038/ncomms9019

66. Paternoster L, Standl M, Waage J, et al (2015) Multi-ancestry genome-wide association study of 21,000 cases and 95,000 controls identifies new risk loci for atopic dermatitis. Nat Genet 47(12):1449–1456. https://doi.org/10.1038/ng.3424

67. Bentham J, Morris DL, Graham DSC, et al (2015) Genetic association analyses implicate aberrant regulation of innate and adaptive immunity genes in the pathogenesis of systemic lupus erythematosus. Nat Genet 47(12):1457–1464. https://doi.org/10.1038/ng.3434

68. Ji S-G, Juran BD, Mucha S, et al (2017) Genome-wide association study of primary sclerosing cholangitis identifies new risk loci and quantifies the genetic relationship with inflammatory bowel disease. Nat Genet 49(2):269–273. https://doi.org/10.1038/ng.3745

69. Estrada K, Styrkarsdottir U, Evangelou E, et al (2012) Genome-wide meta-analysis identifies 56 bone mineral density loci and reveals 14 loci associated with risk of fracture. Nat Genet 44(5):491–501. https://doi.org/10.1038/ng.2249

70. Zheng H-F, Forgetta V, Hsu Y-H, et al (2015) Whole-genome sequencing identifies EN1 as a determinant of bone density and fracture. Nature 526(7571):112–117. https://doi.org/10.1038/nature14878

71. Dastani Z, Hivert M-F, Timpson N, et al (2012) Novel loci for adiponectin levels and their influence on type 2 diabetes and metabolic traits: a multi-ethnic meta-analysis of 45,891 individuals. PLoS Genet 8(3):e1002607. https://doi.org/10.1371/journal.pgen.1002607

72. Nikpay M, Goel A, Won HH, et al (2015) A comprehensive 1,000 Genomes-based genome-wide association meta-analysis of coronary artery disease. NatGenet 47(10):1121–1130. https://doi.org/10.1038/ng.3396

73. Malik R, Traylor M, Pulit SL, et al (2016) Low-frequency and common genetic variation in ischemic stroke: The METASTROKE collaboration. Neurology 86(13):1217–1226. https://doi.org/10.1212/WNL.0000000000002528

74. Saxena R, Hivert MF, Langenberg C, et al (2010) Genetic variation in GIPR influences the glucose and insulin responses to an oral glucose challenge. NatGenet 42(2):142–148. https://doi.org/10.1038/ng.521

75. Dupuis J, Langenberg C, Prokopenko I, et al (2010) New genetic loci implicated in fasting glucose homeostasis and their impact on type 2 diabetes risk. NatGenet 42(2):105–116. https://doi.org/10.1038/ng.520;

76. Soranzo N, Sanna S, Wheeler E, et al (2010) Common variants at 10 genomic loci influence hemoglobin A_1(C) levels via glycemic and nonglycemic pathways. Diabetes 59(12):3229–3239. https://doi.org/10.2337/db10-0502

77. Manning AK, Hivert MF, Scott RA, et al (2012) A genome-wide approach accounting for body mass index identifies genetic variants influencing fasting glycemic traits and insulin resistance. NatGenet 44(6):659–669. https://doi.org/10.1038/ng.2274

78. Morris AP, Voight BF, Teslovich TM, et al (2012) Large-scale association analysis provides insights into the genetic architecture and pathophysiology of type 2 diabetes. NatGenet 44(9):981–990. https://doi.org/10.1038/ng.2383

79. Teumer A, Tin A, Sorice R, et al (2016) Genome-wide Association Studies Identify Genetic Loci Associated With Albuminuria in Diabetes. Diabetes 65(3):803–817. https://doi.org/10.2337/db15-1313

80. Pattaro C, Teumer A, Gorski M, et al (2016) Genetic associations at 53 loci highlight cell types and biological pathways relevant for kidney function. NatCommun 7(Journal Article):10023. https://doi.org/10.1038/ncomms10023

81. Teslovich TM, Musunuru K, Smith AV, et al (2010) Biological, clinical and population relevance of 95 loci for blood lipids. Nature 466(7307):707–713. https://doi.org/10.1038/nature09270

82. Köttgen A, Albrecht E, Teumer A, et al (2013) Genome-wide association analyses identify 18 new loci associated with serum urate concentrations. Nat Genet 45(2):145–154. https://doi.org/10.1038/ng.2500

83. Tobacco and Genetics Consortium (2010) Genome-wide meta-analyses identify multiple loci associated with smoking behavior. NatGenet 42(5):441–447. https://doi.org/10.1038/ng.571

84. Erzurumluoglu AM, Liu M, Jackson VE, et al (2020) Meta-analysis of up to 622,409 individuals identifies 40 novel smoking behaviour associated genetic loci. Mol Psychiatry 25(10):2392–2409. https://doi.org/10.1038/s41380-018-0313-0

85. Huffman JE, Albrecht E, Teumer A, et al (2015) Modulation of genetic associations with serum urate levels by body-mass-index in humans. PLoS One 10(3):e0119752. https://doi.org/10.1371/journal.pone.0119752

ESM Table 1: A total of ten case – control definitions for diabetic kidney disease (DKD; used as a general term to describe renal complications in diabetes)

Phenotype	Cases	Controls	Note
**All vs. Ctrl**	microalbuminuria or macroalbuminuria or ESRD	Normal AER	Phenotype abbreviated as "DN" (diabetic nephropathy) in SUMMIT
**Severe DKD**	macroalbuminuria or ESRD	Normal AER	Phenotype abbreviated as "MACRO" in SUMMIT; as “DN” in DNCRI
**Micro**	microalbuminuria	Normal AER	
**Macro**	macroalbuminuria	Normal AER	Not analyzed in SUMMIT
**ESRD**	ESRD	Normal AER	
**ESRD vs. All**	ESRD	no ESRD	
**ESRD vs. macro**	ESRD	macroalbuminuria	Not analyzed in SUMMIT
**CKD**	eGFR < 60 ml/min/1.73m2	eGFR ≥ 60 ml/min/1.73m2	
**CKD extremes**	ESRD or eGFR < 15 ml/min/1.73m2	eGFR ≥ 60 ml/min/1.73m2	Not analyzed in SUMMIT
**CKD-DKD**	ESRD, or eGFR < 60 ml/min/1.73m2 AND microalbuminuria or macroalbuminuria	normal AER and eGFR ≥ 60 ml/min/1.73m2	Some cohorts in SUMMIT required at least one measurement with “high microalbuminruia”, i.e. AER ≥150 mg/24, or equivalent

The subjects were classified as normal AER, microalbuminuria or macroalbuminuria based on two out of three consecutive urine samples surpassing the required thresholds:

Normal AER	AER <20 µg/min (overnight collection) OR<br>AER <30 mg/24 h (24h urine collection) OR<br>ACR <2.5 mg/mmol for men<br>ACR <3.5 for women (spot/ any urine)<br>Diabetes duration ≥10 years for type 2 diabetes, ≥ 15 years for type 1 diabetes
Microalbuminuria	AER ≥20 AND <100 µg/min (overnight collection) OR<br>AER ≥30 AND <150 mg/24 hr (24h urine collection) OR<br>ACR ≥2.5 AND <12.5 for men<br>ACR ≥3.5 AND <17.5 for women (spot/ any urine).
Macroalbuminuria	AER ≥200 µg/min (overnight collection) OR<br>AER ≥300 mg/24 h (24h urine collection) OR<br>ACR ≥25 mg/mmol for men<br>ACR ≥35 for women (spot/ any urine)<br>*Due to study designs, in some SUMMIT studies one measurement above these thresholds was sufficient.

ESRD: End-stage renal disease, dialysis or renal transplant (or eGFR< 15 ml/min/1.73m2 in SUMMIT)
eGFR was estimated based on serum creatinine and calculated either with the MDRD4(ref [43]) or the CKD-EPI[44] formula depending on the study. In SUMMIT, when IDMS-calibrated serum creatinine was used, the MDRD4 formula was multiplied by 175/186.[45]

ESM Table 2: Number of individuals in each cohort for each phenotypic comparison

Study	Severe DKD			Macro			ESRD			ESRD vs. All			ESRD vs. Macro			All vs. Ctrl			Micro			CKD			CKD extremes			CKD-DKD		
**DNCRI**	**Cases**	**Ctrls**	**Total**	**Cases**	**Ctrls**	**Total**	**Cases**	**Ctrls**	**Total**	**Cases**	**Ctrls**	**Total**	**Cases**	**Ctrls**	**Total**	**Cases**	**Ctrls**	**Total**	**Cases**	**Ctrls**	**Total**	**Cases**	**Ctrls**	**Total**	**Cases**	**Ctrls**	**Total**	**Cases**	**Ctrls**	**Total**
Austria	6	71	**77**	4	71	**75**	2	71	**73**	2	88	**90**	2	4	**6**	19	71	**90**	13	71	**84**	11	80	**91**	2	80	**82**	6	66	**72**
CACTI	35	422	**457**	29	422	**451**	6	422	**428**	6	503	**509**	6	29	**35**	87	422	**509**	52	422	**474**	45	477	**522**	6	477	**483**	24	407	**431**
EDIC	134	193	**327**	75	193	**268**	59	193	**252**	59	359	**418**	59	75	**134**	225	193	**418**	91	193	**284**	130	288	**418**	59	288	**347**	89	180	**269**
EDC	84	1016	**1100**	61	1016	**1077**	23	1016	**1039**	23	1282	**1305**	23	61	**84**	289	1016	**1305**	205	1016	**1221**	80	1218	**1298**	23	1218	**1241**	47	999	**1046**
FinnDiane	1371	2240	**3611**	535	2268	**2803**	854	2265	**3119**	854	3418	**4272**	854	517	**1371**	2069	2202	**4271**	719	2257	**2976**	1226	3038	**4264**	838	3069	**3907**	993	2066	**3059**
FRANCE	332	627	**959**	181	625	**806**	151	627	**778**	151	920	**1071**	151	181	**332**	448	627	**1075**	124	625	**749**	281	740	**1021**	159	740	**899**	225	578	**803**
GWU GOKIND	290	311	**601**	29	311	**340**	261	311	**572**	261	340	**601**	261	29	**290**	290	311	**601**	0	311	**311**	273	325	**598**	261	325	**586**	269	309	**578**
ITALY	180	161	**341**	38	161	**199**	142	161	**303**	142	201	**343**	142	38	**180**	180	163	**343**	0	161	**161**	157	172	**329**	148	175	**323**	155	154	**309**
JOSLIN	719	1082	**1801**	475	1082	**1557**	244	1082	**1326**	244	2027	**2271**	244	475	**719**	1189	1082	**2271**	470	1082	**1552**	533	1574	**2107**	262	1695	**1957**	402	1013	**1415**
LatDiane	25	80	**105**	18	80	**98**	7	80	**87**	7	131	**138**	7	18	**25**	58	80	**138**	33	80	**113**	16	109	**125**	7	109	**116**	9	78	**87**
LitDiane	19	39	**58**	9	39	**48**	10	39	**49**	10	69	**79**	10	9	**19**	40	39	**79**	21	39	**60**	21	50	**71**	10	50	**60**	16	36	**52**
RomDiane	98	89	**187**	70	89	**159**	28	89	**117**	28	207	**235**	28	70	**98**	146	89	**235**	48	89	**137**	53	167	**220**	28	167	**195**	39	87	**126**
Scotland	195	3962	**4157**	144	3984	**4128**	57	3962	**4019**	57	4632	**4689**	57	138	**195**	727	3962	**4689**	540	3984	**4524**	404	4712	**5116**	80	4712	**4792**	90	4450	**4540**
STENO	488	414	**902**	469	414	**883**	19	414	**433**	19	897	**916**	19	470	**489**	489	427	**916**	0	414	**414**	200	690	**890**	28	690	**718**	106	398	**504**
SWEDEN	51	346	**397**	35	346	**381**	20	346	**366**	20	497	**517**	20	32	**52**	51	346	**397**	85	346	**431**	42	287	**329**	20	287	**307**	21	252	**273**
UK_ROI	704	730	**1434**	466	730	**1196**	200	730	**930**	200	1196	**1396**	200	466	**666**	704	730	**1434**	0	730	**730**	587	513	**1100**	200	513	**713**	266	433	**699**
WESDR	217	293	**510**	113	293	**406**	104	293	**397**	104	452	**556**	104	113	**217**	263	293	**556**	46	293	**339**	207	398	**605**	104	398	**502**	140	260	**400**
**Total DNCRI**	**4,948**	**12,076**	**17,024**	**2,751**	**12,124**	**14,875**	**2,187**	**12,101**	**14,288**	**2,187**	**17,219**	**19,406**	**2,187**	**2,725**	**4,912**	**7,274**	**12,053**	**19,327**	**2,447**	**12,113**	**14,560**	**4,266**	**14,838**	**19,104**	**2,235**	**14,993**	**17,228**	**2,897**	**11,766**	**14,663**
**SUMMIT-1**	**Cases**	**Ctrls**	**Total**	**Cases**	**Ctrls**	**Total**	**Cases**	**Ctrls**	**Total**	**Cases**	**Ctrls**	**Total**	**Cases**	**Ctrls**	**Total**	**Cases**	**Ctrls**	**Total**	**Cases**	**Ctrls**	**Total**	**Cases**	**Ctrls**	**Total**	**Cases**	**Ctrls**	**Total**	**Cases**	**Ctrls**	**Total**
Eurodiab	203	491	**694**				84	491	**575**	84	705	**789**				298	491	**789**	95	491	**586**	113	467	**580**				210	357	**567**
NFS-ORPS	47	199	**246**													197	199	**396**	150	199	**349**									
SDR	168	292	**460**	85	277	**362**	75	294	**369**	75	529	**604**	57	85	**142**	266	290	**556**	98	290	**388**	163	365	**528**	57	349	**406**	118	239	**357**
**Total SUMMIT-1**	**418**	**982**	**1,400**	**85**	**277**	**362**	**159**	**785**	**944**	**159**	**1,234**	**1,393**	**57**	**85**	**142**	**761**	**980**	**1,741**	**343**	**980**	**1,323**	**276**	**832**	**1,108**	**57**	**349**	**406**	**328**	**596**	**924**
**SUMMIT-2**	**Cases**	**Ctrls**	**Total**	**Cases**	**Ctrls**	**Total**	**Cases**	**Ctrls**	**Total**	**Cases**	**Ctrls**	**Total**	**Cases**	**Ctrls**	**Total**	**Cases**	**Ctrls**	**Total**	**Cases**	**Ctrls**	**Total**	**Cases**	**Ctrls**	**Total**	**Cases**	**Ctrls**	**Total**	**Cases**	**Ctrls**	**Total**
GoDARTS Affy	218	816	**1,034**	138	816	**954**	80	816	**896**	48	1,491	**1,539**	80	138	**218**	885	816	**1,701**	667	816	**1,483**	1,025	1,553	**2,578**	80	1,101	**1181**	168	716	**884**
GoDARTS Illumina	179	680	**859**	130	675	**805**	48	680	**728**	80	1,621	**1,701**	48	130	**178**	859	680	**1,539**	680	680	**1,360**	972	513	**1,485**	48	1092	**1140**	120	587	**707**
MNI	66	165	**231**													188	165	**353**	122	162	**284**									
SDR	713	580	**1,292**	424	556	**980**	243	580	**823**	243	1,359	**1,602**	268	424	**692**	1,250	580	**1,830**	520	580	**1,100**	997	666	**1,663**	240	628	**868**	609	307	**916**
Steno	163	131	**294**													163	131	**294**				100	174	**274**						
**Total SUMMIT-2**	**1,339**	**2,372**	**3,710**	**692**	**2,047**	**2,739**	**371**	**2,076**	**2,447**	**371**	**4,471**	**4,842**	**396**	**692**	**1,088**	**3,345**	**2,372**	**5,717**	**1,989**	**2,238**	**4,227**	**3,094**	**2,906**	**6,000**	**368**	**2,821**	**3,189**	**897**	**1,610**	**2,507**
**Total ALL**	**6,705**	**15,430**	**22,134**	**3,528**	**14,448**	**17,976**	**2,717**	**14,962**	**17,679**	**2,717**	**22,924**	**25,641**	**2,640**	**3,502**	**6,142**	**11,380**	**15,405**	**26,785**	**4,779**	**15,331**	**20,110**	**7,636**	**18,576**	**26,212**	**2,660**	**18,163**	**20,823**	**4,122**	**13,972**	**18,094**

Number of samples in the SUMMIT-1 (type 1 diabetes) and SUMMIT-2 (type 2 diabetes) cohorts represents those individuals included in the original analysis, containing related individuals; The current meta-analysis was based on effect size estimates from SUMMIT which were derived after excluding related individuals (see **ESM** Table 3 analysis method).

ESM Table 3: Key characteristics of the genotyping and statistical analyses in DNCRI and SUMMIT cohorts.

	DNCRI	SUMMIT-1 and SUMMIT-2
N studies	17	3 (SUMMIT-1), 5 (SUMMIT-2)
N samples (max)	19,406	1,741 (SUMMIT-1), 6,000 (SUMMIT-2)
Genotyping chips	HumanCoreExome Bead arrays 12-1.0, 12-1.1, and 24-1.0	Illumina Omni express array, Affymetrix SNP 6.0 array, Illumina 610Quad assay
Genotype calling	zCall	GenomeStudio, CHIAMO
Main pre-imputation Quality control filters	SNP call rates <95%, excessive deviation from Hardy–Weinberg equilibrium; sample call rates <98%, sex mismatch, extreme heterozygosity, principal component analysis to exclude outliers with evidence of non-European ancestry	SNP call rate <95%, MAF<1%, HWE $p<10^{-6}$ or $p<10^{-7}$, evidence of plate differences (p<1e-7). Sample call rate <95%, extremely high/low heterozygosity (>3 sd. or >4 sd. from mean), Admixture (PC1 or PC2 > 6 sd. away from mean + visual evaluation)
Imputation reference panel	1000Genomes Phase 3	1000Genomes Phase 1
Imputation software	Minimac3/Minimac3-omp (version 1.0.14)	Prephasing with SHAPE-IT v2; Imputation with IMPUTEv2
Covariates	Age, sex, diabetes duration, genetic principal components, study specific covariates (e.g. site or genotyping batch). Excluded closely related individuals.	Age, gender, duration of diabetes, genetic principal components.<br>Excluded closely related individuals.
Main post-analysis SNP QC filters	•Imputation quality score: INFO ≥ 0.3<br>•Minor allele count ≥ 10 in cases and in controls<br>•Marker must be present in at least 2 studies	•Imputation quality score: INFO ≥ 0.4<br>•Minor allele count ≥ 10 in cases and in controls<br>•MAF ≥ 0.01<br>•SUMMIT-2: Marker must be present in at least 2 studies (not applied for the three additional phenotypes available only in SDR, GoDARTS 1 and 2)
Analysis method and software	SNPtest, additive score test	SNPtest, additive score test. Note: in original studies, *P* values of association were estimated using EMMAX mixed model including related individuals, while only effect size estimates were obtained from SNPtest (excluding related individuals).
Meta-analysis	Inverse-variance fixed effects meta-analysis (METAL software)	Inverse-variance fixed effects meta-analysis (GWAMA or METAL)

ESM Table 4: List of related traits studied with LD score regression.

Trait	PMID	Category	ethnicity
Mothers age at death	27015805	aging	European
Parents age at death	27015805	aging	European
Fathers age at death	27015805	aging	European
Height_2010	20881960	anthropometric	European
Body mass index	20935630	anthropometric	European
Childhood obesity	22484627	anthropometric	European
Infant head circumference	22504419	anthropometric	European
Child birth weight	23202124	anthropometric	European
Difference in height between childhood and adulthood; age 8	23449627	anthropometric	European
Difference in height between adolescence and adulthood; age 14	23449627	anthropometric	European
Height; Females at age 10 and males at age 12	23449627	anthropometric	European
Obesity class 1	23563607	anthropometric	European
Overweight	23563607	anthropometric	European
Obesity class 2	23563607	anthropometric	European
Obesity class 3	23563607	anthropometric	European
Extreme bmi	23563607	anthropometric	European
Extreme height	23563607	anthropometric	European
Extreme waist-to-hip ratio	23563607	anthropometric	European
Child birth length	25281659	anthropometric	European
Waist-to-hip ratio	25673412	anthropometric	European
Waist circumference	25673412	anthropometric	European
Hip circumference	25673412	anthropometric	European
Sitting height ratio	25865494	anthropometric	European
Body fat	26833246	anthropometric	Mixed
Birth weight	27680694	anthropometric	European
Offspring birth weight	31043758	anthropometric	European
Offspring birth weight (maternal effect)	31043758	anthropometric	European
Offspring birth weight	31043758	anthropometric	Mixed
Own birth weight	31043758	anthropometric	European
Own birth weight	31043758	anthropometric	Mixed
Own birth weight (fetal effect)	31043758	anthropometric	European
Asthma	17611496	autoimmune	European
Celiac disease	20190752	autoimmune	European
Multiple sclerosis	21833088	autoimmune	European
Rheumatoid Arthritis	24390342	autoimmune	European
Inflammatory Bowel Disease (Euro)	26192919	autoimmune	European
Crohns disease	26192919	autoimmune	European
Ulcerative colitis	26192919	autoimmune	European
Primary biliary cirrhosis	26394269	autoimmune	European
Eczema	26482879	autoimmune	Mixed
Systemic lupus erythematosus	26502338	autoimmune	European
Primary sclerosing cholangitis	27992413	autoimmune	Mixed
Femoral neck bone mineral density	22504420	bone	Mixed
Lumbar spine bone mineral density	22504420	bone	Mixed
Lumbar Spine bone mineral density	26367794	bone	Mixed
Femoral Neck bone mineral density	26367794	bone	Mixed
Forearm Bone mineral density	26367794	bone	Mixed
Adiponectin	22479202	cardiometabolic	Mixed
Coronary artery disease	26343387	cardiometabolic	Mixed
Ischemic stroke	26935894	cardiometabolic	Mixed
2hr glucose adjusted for BMI	20081857	glycemic	European

HOMA-IR	20081858	glycemic	European
HOMA-B	20081858	glycemic	European
Fasting proinsulin	20081858	glycemic	European
HbA1C	20858683	glycemic	European
Fasting glucose main effect	22581228	glycemic	European
Fasting insulin main effect	22581228	glycemic	European
Type 2 Diabetes	22885922	glycemic	European
Urinary albumin-to-creatinine ratio	26631737	kidney	European
Urinary albumin-to-creatinine ratio (non-diabetes)	26631737	kidney	European
Chronic Kidney Disease	26831199	kidney	Mixed
Serum cystatin c	26831199	kidney	Mixed
Serum creatinine	26831199	kidney	Mixed
Serum creatinine (non-diabetes)	26831199	kidney	Mixed
Triglycerides	20686565	lipids	European
HDL cholesterol	20686565	lipids	European
LDL cholesterol	20686565	lipids	European
Total Cholesterol	20686565	lipids	European
Urate	23263486	other	European
Former vs Current smoker	20418890	smoking_behaviour	European
Ever vs never smoked	20418890	smoking_behaviour	European
Cigarettes smoked per day	20418890	smoking_behaviour	European
Age of smoking initiation	20418890	smoking_behaviour	European
Smoking Initiation	30617275	smoking_behaviour	European
Smoking Cessation	30617275	smoking_behaviour	European
Cigarettes Per Day	30617275	smoking_behaviour	European
Pack Years	30617275	smoking_behaviour	European
Serumurate overweight	25811787	uric_acid	European

PMID: PubMed ID. See list of references on ESM References, page 11.

27015805 [46]
20881960 [47]
20935630 [48]
22484627 [49]
22504419 [50]
23202124 [51]
23449627 [52]
23563607 [53]
25281659 [54]
25673412 [55]
25865494 [56]
26833246 [57]
27680694 [58]
31043758 [59]
17611496 [60]
20190752 [61]
21833088 [62]
24390342 [63]
26192919 [64]
26394269 [65]
26482879 [66]
26502338 [67]
27992413 [68]
22504420 [69]
26367794 [70]
22479202 [71]
26343387 [72]
26935894 [73]
20081857 [74]
20081858 [75]
20858683 [76]
22581228 [77]
22885922 [78]
26631737 [79]
26831199 [80]
20686565 [81]
23263486 [82]
20418890 [83]
30617275 [84]
25811787 [85]

ESM Table 5: Association details for the novel genome-wide significant locus rs72831309 in *TENM2*. In the meta-analysis, rs72831309 (chr5:166978230) minor A allele was considered the effect allele, major G as the non-effect allele.

COHORT	EAF	BETA	SE	OR	OR_L95	OR_U95	P	INFO	N
GWU_GOKIND	0.02	-0.11	0.76	0.88	0.20	3.93	0.88	0.38	578
UK_ROI	0.03	0.57	0.49	1.40	0.53	3.68	0.25	0.53	699
JOSLIN	0.03	0.33	0.36	1.15	0.57	2.33	0.36	0.51	1415
FinnDiane	0.05	0.82	0.17	1.60	1.15	2.22	1.0×10^{-6}	0.66	3059
EURODIAB	0.03	1.36	0.51	3.89	1.43	10.54	0.008	0.52	567
SUMMIT-2 meta	0.03	0.69	0.31	1.99	1.08	3.67	0.028	NA	1552
**Meta all**	**0.04**	**0.73**	**0.13**	**2.08**	1.62	2.67	**9.82×10^{-9}**		**8322**

EAF: effect allele (minor A allele) frequency. BETA: effect size estimate. SE: standard error for BETA. OR: odds ratio. OR_L95 and OR_U95: Lower and upper confidence intervals. P: p-value. INFO: imputation quality info metrics. N: Number of samples in the study.

ESM Table 6: Highly correlated reconstituted gene-sets that make-up the "basement membrane" meta-gene set derived in Marouli et al. The "Genes prioritized" column contains all genes prioritized in MAGMA by one of these 26 gene-sets, including the "FBLN2 PPI subnetwork" gene set that prioritized *COL4A3* for Severe DKD.

GENE-SET ID	GENE-SET DESCRIPTION	GENES PRIORITIZED
MP:0003044	impaired basement membrane formation	
**ENSG00000163520**	**FBLN2 PPI subnetwork**	*ADAMTS15, CD248,* ***COL4A3****, NID2*
MP:0004272	abnormal basement membrane morphology	
GO:0043256	laminin complex	
ENSG00000130702	LAMA5 PPI subnetwork	
ENSG00000132561	MATN2 PPI subnetwork	
ENSG00000091136	LAMB1 PPI subnetwork	
ENSG00000116962	NID1 PPI subnetwork	
ENSG00000135862	LAMC1 PPI subnetwork	
ENSG00000168487	BMP1 PPI subnetwork	
GO:0034446	substrate adhesion-dependent cell spreading	
ENSG00000125810	CD93 PPI subnetwork	
ENSG00000134871	COL4A2 PPI subnetwork	*ABCC9, CCDC102B, COL18A1, CSPG4, CTHRC1, FN1, IGFBP3, LAMC1, LOXL1, OLFML2B, TGFBI*
ENSG00000188153	COL4A5 PPI subnetwork	*ABCA9, ADAMTS5, AEBP1, ART3, BICC1, C3, C7, COL15A1, COL1A1, COL1A2, COL3A1,* ***COL4A3****, COL5A2, COL6A2, ENSG00000259134, ENSG00000259284, FBLN5, FBN1, FIBIN, FNDC1, FSTL1, GALNTL4, GRB14, IGFBP6, LAMA2, LAMB1, LINC00312, LOX, MMP2, NOV, OLFML1, POSTN, SCN7A, SERPING1, SLIT3, SPARC, THBS2, VGLL3, WDR72*
ENSG00000112773	FAM46A PPI subnetwork	
ENSG00000100985	MMP9 PPI subnetwork	
ENSG00000110492	MDK PPI subnetwork	
ENSG00000101680	LAMA1 PPI subnetwork	
GO:0043236	laminin binding	
ENSG00000187498	COL4A1 PPI subnetwork	*ACTA2, COL14A1, COL4A2, CTHRC1, ENPEP, LHFP, LOXL2, TGFBI*
GO:0005605	basal lamina	
ENSG00000213949	ITGA1 PPI subnetwork	
ENSG00000114270	COL7A1 PPI subnetwork	
GO:0050840	extracellular matrix binding	
ENSG00000081052	COL4A4 PPI subnetwork	*ABCC9, ACTA2, ADAMTS1, ADAMTS5, ASPN, C1R, C1S, C7, CCDC80, COL4A1, COL5A1, COL6A1, DCN, EFEMP1, FBLN1, FKBP7, IGFBP3, LGALS1, LOX, LUM, MGP, NID2, PID1, PXDN, SCN7A, SERPINF1, SPARCL1, VCAN*
GO:0005604	basement membrane	*APLNR, COL12A1, CTGF, HTRA1, ITGB4, ITGB6, MCAM, PRSS23*

ESM Table 7: Association results for the lead SNPs at the MAGMA/PASCAL genes.

Phenotype	Gene(s)	CHR:POS	SNP	EA	NEA	EAF	OR (95% CI)	*P*-value	P_{HET}
CKD extremes	*COL20A1*	20:61964452	rs6011746	C	G	0.923	0.69 (0.59 - 0.8)	1.90×10^{-6}	0.65
ESRD vs. All	*COL20A1*	20:61950071	rs74397198	A	G	0.078	1.44 (1.24 - 1.69)	3.58×10^{-6}	1
ESRD vs. macro	*DCLK1*	13:36542599	rs12428319	T	C	0.553	1.2 (1.1 - 1.31)	3.18×10^{-5}	0.78
ESRD vs. macro	*EIF4E*	4:99796439	rs7664964	T	C	0.606	0.81 (0.74 - 0.88)	8.92×10^{-7}	0.88
Severe DKD	*GPR158*	10:25590161	rs532538	T	C	0.731	0.88 (0.84 - 0.93)	2.96×10^{-6}	0.0053*
All vs. Ctrl	*INIP/SNX30*	9:115429626	rs786959	A	G	0.105	1.18 (1.11 - 1.27)	9.91×10^{-7}	0.63
Severe DKD	*LSM14A*	19:34701331	rs1260634	T	C	0.371	1.12 (1.07 - 1.17)	5.22×10^{-6}	0.94
Severe DKD	*MFF*	2:228121101	rs55703767	T	G	0.208	0.82 (0.77 - 0.87)	3.60×10^{-11}	0.11
CKD	*PTPRN/RESP18*	2:220178435	rs2090163	T	C	0.728	1.14 (1.08 - 1.21)	1.01×10^{-6}	0.46

EA: Effect allele. NEA: Non-effect allele. EAF: Effect allele frequency. P_{HET}: P-value for heterogeneity between the type 1 diabetes and type 2 diabetes studies.

*Type 1 diabetes: N=18,589, p=0.002, OR [95% CI] = 1.10 [1.03 - 1.16]; Type 2 diabetes: N=3,461, p=7.41×10^{-6}, OR [95% CI] = 1.34 [1.18 - 1.52]

ESM Table 8: TWAS results with $p<1\times10^{-4}$. P-values < 4.1×10^{-6} (in bold) are significant after correction for multiple testing.

				TWAS association			Prediction performance				
Tissue	Pheno	gene	gene_name	Effect	p-value	var_g	r2	p-value	q-value	n_snps_used	n_snps_in_model
tub	Severe DKD	ENSG00000135334.8	*AKIRIN2*	0.308	***1.11E-06***	0.092	0.05	0.013	0.012	39	42
tub	Macro	ENSG00000135334.8	*AKIRIN2*	0.383	***1.70E-06***	0.092	0.05	0.013	0.012	39	42
glom	Micro	ENSG00000268208.1	*AC008372.1*	-7.489	1.59E-05	0.000	0.03	0.083	0.044	1	1
glom	Macro	ENSG00000228696.4	*ARL17B*	-0.195	2.06E-05	0.331	0.51	1.28E-19	1.77E-18	54	65
glom	Micro	ENSG00000138028.10	*CGREF1*	-0.228	2.15E-05	0.131	0.14	2.08E-05	3.07E-05	25	26
glom	CKD	ENSG00000078804.8	*TP53INP2*	0.499	2.73E-05	0.068	0.03	0.080	0.042	34	38
glom	Macro	ENSG00000227057.3	*WDR46*	-0.583	4.05E-05	0.024	0.03	0.085	0.044	10	10
tub	Severe DKD	ENSG00000205269.4	*TMEM170B*	0.192	4.62E-05	0.133	0.06	0.007	0.007	90	95
tub	CKD	ENSG00000188283.7	*ZNF383*	0.327	4.72E-05	0.046	0.05	0.012	0.011	15	16
tub	CKD	ENSG00000075413.13	*MARK3*	-0.258	5.05E-05	0.085	0.05	0.013	0.012	102	104
tub	Macro	ENSG00000162836.7	*ACP6*	-0.146	6.44E-05	0.377	0.47	4.19E-18	7.16E-17	30	31

Tissue: tub(ular) or glom(erular). TWAS association: Effect: association effect size for the gene. P-value: p-value for the TWAS association. var_g: variance of the gene expression. Prediction performance r2, p-value and q-value: statistics for tissue model's correlation to gene's measured transcriptome; n_snps_used: number of SNPs from GWAS that were used in the analysis; n_snps_in_model: number of SNPs in the model (i.e. in the transcriptomics data). Macro, macroalbuminuria vs normal AER; Micro, microalbuminuria vs normal AER.

ESM Table 8b: TWAS results look-up for lead SNPs in kidney tubular and glomerular eQTL data (Qiu et al. 2018, [15]) and GWAS on eGFR in the general population (Wuttke et al. 2019, [39]). Results with p<0.05 are shown.

				TWAS association			Prediction performance				
Tissue	Pheno	gene	gene_name	Effect	p-value	var_g	r2	p-value	q-value	n_snps_used	n_snps_in_model
tub	eGFR	ENSG00000148158.12	*SNX30*	0.0011	0.046	0.18	0.10	0.0003	0.00058	73	82

ESM Table 9: eQTL and association data for SNPs that were selected by elastic net regression model in TWAS contributing to AKIRIN2 TWAS association.

			eQTL				Association with Severe DKD								
SNP	Chr	Pos	REF	ALT	Weight	-	EA	NEA	EA Freq	Effect	SE	P	N	-	eQTL Weight for risk allele
rs58305152	6	88340568	G	A	-0.001		A	G	0.274	-0.101	0.027	0.0002	22045		0.001
rs12529993	6	88283420	A	C	-0.071		A	C	0.725	0.099	0.027	0.0003	22048		0.071
rs9450740	6	88273971	G	A	-0.082		A	G	0.274	-0.099	0.027	0.0003	22046		0.082
rs2245604	6	88498604	G	T	-0.194		T	G	0.853	-0.141	0.041	0.0005	21592		0.194
rs10944332	6	88492526	A	G	0.055		A	G	0.865	-0.132	0.042	0.0015	21593		0.055
rs4706308	6	88825342	G	T	-0.061		T	G	0.653	-0.095	0.033	0.0038	22107		0.061
rs4707394	6	88453883	G	A	-0.047		A	G	0.536	-0.059	0.024	0.014	22103		0.047
rs9342120	6	88455481	A	G	-0.046		A	G	0.465	0.058	0.024	0.016	22107		0.046
rs9444543	6	88450687	G	A	-0.046		A	G	0.535	-0.058	0.024	0.016	22103		0.046
rs9344715	6	88448510	A	G	-0.044		A	G	0.465	0.057	0.024	0.017	22105		0.044
rs72914432	6	87758995	C	T	0.135		T	C	0.044	0.174	0.077	0.024	19226		0.135
rs118049447	6	87717596	G	A	0.066		A	G	0.043	0.177	0.079	0.026	18934		0.066
rs7738899	6	88481411	T	G	-0.002		T	G	0.394	0.054	0.026	0.037	22101		0.002
rs13205684	6	88478101	C	T	-0.017		T	C	0.607	-0.052	0.025	0.043	22101		0.017
rs56200744	6	87624007	C	A	0.003		A	C	0.145	0.071	0.042	0.095	21939		0.003
rs2787889	6	88505746	T	C	-0.028		T	C	0.199	0.055	0.034	0.099	21944		0.028
rs4707329	6	87572226	G	T	-0.004		T	G	0.107	-0.067	0.041	0.099	21483		0.004
rs9450545	6	87583661	T	C	-0.014		T	C	0.887	0.066	0.040	0.103	21484		0.014
rs79344675	6	87586002	A	G	-0.005		A	G	0.891	0.063	0.041	0.121	21485		0.005
rs202220103	6	87610476	T	C	-0.137		T	C	0.904	0.073	0.049	0.139	16327		0.137
rs138083000	6	87587532	T	C	-0.003		T	C	0.891	0.059	0.041	0.145	21483		0.003
rs17731731	6	87928971	G	A	0.038		A	G	0.084	0.076	0.053	0.153	21090		0.038
rs6927401	6	89043905	A	G	0.024		A	G	0.573	-0.026	0.024	0.297	22107		0.024
rs806377	6	88858723	T	C	-0.031		T	C	0.486	-0.025	0.025	0.300	22102		-0.031
rs9353542	6	89041259	G	T	0.024		T	G	0.428	0.025	0.025	0.304	22103		0.024
rs12191012	6	89039844	G	A	0.039		A	G	0.104	0.033	0.043	0.434	20900		0.039
rs1049353	6	88853635	C	T	-0.013		T	C	0.285	-0.022	0.029	0.456	22048		0.013
rs9344742	6	88626153	T	C	0.054		T	C	0.392	0.019	0.028	0.499	18646		-0.054
rs34719676	6	87415764	G	A	0.012		A	G	0.128	0.026	0.038	0.504	22046		0.012
rs7769951	6	89206945	T	C	-0.025		T	C	0.087	0.031	0.047	0.508	20384		0.025
rs62430887	6	89219617	G	A	0.016		A	G	0.086	0.030	0.047	0.526	20385		0.016
rs36060042	6	87437974	T	C	0.050		T	C	0.874	-0.024	0.038	0.534	22046		0.050
rs4076053	6	88626861	G	A	0.032		A	G	0.603	-0.016	0.028	0.571	18646		-0.032
rs28825134	6	89048365	C	A	0.038		A	C	0.097	0.029	0.052	0.572	15743		0.038
rs199700576	6	89231738	A	G	0.020		A	G	0.318	-0.017	0.031	0.574	16889		0.020
rs73484048	6	88535200	A	G	-0.008		A	G	0.665	0.013	0.029	0.654	22044		0.008
rs78952394	6	88868594	T	C	-0.015		T	C	0.881	0.017	0.040	0.660	21545		0.015
rs7763877	6	88206513	G	A	-0.019		A	G	0.909	-0.018	0.065	0.778	15930		0.019
rs3798787	6	89337530	G	A	-0.130		A	G	0.116	-0.010	0.042	0.812	21090		0.130
rs7382639	6	89274654	A	G	-0.033		A	G	0.802	0.008	0.033	0.820	21938		0.033
rs9344742	6	88626153	T	C	0.054		T	C	0.394	0.011	0.057	0.843	3457		-0.054
rs12215366	6	88989076	T	G	0.096		T	G	0.913	-0.005	0.048	0.911	20900		0.096
rs4076053	6	88626861	G	A	0.032		A	G	0.607	-0.002	0.056	0.975	3456		-0.032

eQTL Weight: weights for AKIRIN2 gene (ENSG00000135334.8) from tubule eQTL dataset, for the Alt (alternative) allele. Association: EA: effect allele; NEA: non-effect allele. EA Freq: effect allele frequency. Effect: effect size beta for EA, such that positive effect indicates higher EA frequency in Severe DKD cases. SE: effect size standard error. P: SNP – Severe DKD association p-value. N: number of samples contributing to association. eQTL weight for risk allele: eQTL weight for the Severe DKD risk increasing allele (not necessarily the effect allele).

ESM Table 10: Kidney eQTL associations with p<1×10^{-4} in tubular or glomerular eQTL data, or in the kidney eQTL meta-analysis for the lead SNPs. Three top SNPs were queried for each lead locus from GWAS meta-analysis, gene-level (MAGMA or PASCAL) analysis, or TWAS locus. In addition, eQTL associations with p<0.01 in tubular or glomerular eQTL data, or in the kidney eQTL meta-analysis are given for the SNPs reaching genome-wide significance ($p<5\times10^{-8}$) in the GWAS meta-analysis.

Tissue	SNP	CHR:POS	P eQTL	eQTL GENE	Index gene	P GWAS	PP eQTL
Glomerular	rs28577966	4:99796005	2.13E-07	*ADH4*	*EIF4E*	1.05E-06	0.698
Glomerular	rs7664964	4:99796439	2.13E-07	*ADH4*	*EIF4E*	8.92E-07	0.698
Glomerular	rs11725932	4:99799310	2.13E-07	*ADH4*	*EIF4E*	9.97E-07	na
Tubular	rs59113552	6:88236233	5.19E-05	*SMIM8*	*AKIRIN2*	2.85E-05	na
Kidney meta	rs786959	9:115429626	4.59E-07	*SNX30*	*INIP*	9.913E-07	0.697
Kidney meta	rs6011746	20:61964452	5.75E-05	*CHRNA4*	*COL20A1*	1.90E-06	0.00
**eQTL associations with p<0.01 for GWAS lead SNPs with p<5×10^{-8}**							
Glomerular	rs55703767	2:228121101	0.0091	*AC010735*	*COL4A3*	3.60E-11	
Kidney meta	rs72831309	5: 166978230	0.0069	*TENM2-AS1*	*TENM2*	9.82E-09	

Tissue: kidney eQTL meta-analysis, or glomerular or tubule compartment-specific expression.
PP eQTL: posterior probability of colocalization for the GWAS and eQTL signal in a kidney meta-analysis of 686 samples.

ESM Table 11: Significant kidney mQTL associations ($p<1.46\times10^{-11}$ Bonferroni-adjusted genome-wide significance; 1×10^{-7} suggestive significance) for lead loci. Three most significant SNPs were queried at each associated loci from single variant, gene-level, and transcriptome-wide association study (TWAS).

Chr	RSID	SNP_Pos	CpG	CpG_Start	P mQTL	PP mQTL	Gene	P DKD/eGFR
19	rs668933	34704936	cg14143166	34716204	1.94E-28	0.804	*LSM14A*	0.03 (DKD)
19	rs1260634	34701331	cg14143166	34716204	2.09E-28	0.804	*LSM14A*	0.03 (DKD)
19	rs535440	34694581	cg14143166	34716204	2.12E-28	0.804	*LSM14A*	0.03 (DKD)
13	rs12428319	36542599	cg21746263	36562319	6.81E-22	0.821	*DCLK1*	
13	rs61948262	36533891	cg21746263	36562319	4.19E-21	0.821	*DCLK1*	
20	rs117255010	61953366	cg20706388	61958549	1.79E-12	*na*	*COL20A1*	
20	rs6011746	61964452	cg20706388	61958549	2.14E-12	0.916	*COL20A1*	
20	rs4809528	61943661	cg20706388	61958549	2.55E-12	0.916	*COL20A1*	
20	rs74397198	61950071	cg20706388	61958549	2.88E-12	0.916	*COL20A1*	
20	rs143391037	61971717	cg20706388	61958549	4.76E-12	*na*	*COL20A1*	
2	rs6436131	220151858	cg06895971	220147671	9.20E-12	*Na*	*PTPRN*	
4	rs7664964	99796439	cg25974308	99852386	1.10E-11	0.003	*EIF4E*	0.041 (eGFR slope)
4	rs28577966	99796005	cg25974308	99852386	1.10E-11	0.003	*EIF4E*	0.041 (eGFR slope)
4	rs11725932	99799310	cg25974308	99852386	1.10E-11	0.003	*EIF4E*	0.041 (eGFR slope)
6	rs34472900	88405040	cg00551398	88298473	1.12E-11	*na*	*AKIRIN2*	
6	rs151077971	88405605	cg00551398	88298473	1.13E-11	*na*	*AKIRIN2*	
6	rs59113552	88236233	cg00551398	88298473	1.58E-11	*na*	*AKIRIN2*	
9	rs786975	115451231	cg13293976	115516494	2.20E-11		*INIP*	0.012 (eGFR slope)
2	rs4674377	220201272	cg14891200	220197663	2.72E-10		*RESP18*	
2	rs2090163	220178435	cg14891200	220197663	1.06E-09		*PTPRN*	
6	rs59113552	88236233	cg10313604	88493367	3.18E-09		*AKIRIN2*	
6	rs151077971	88405605	cg10313604	88493367	7.06E-09		*AKIRIN2*	
6	rs34472900	88405040	cg10313604	88493367	7.11E-09		*AKIRIN2*	
6	rs151077971	88405605	cg05834092	87792915	7.82E-09		*AKIRIN2*	
6	rs34472900	88405040	cg05834092	87792915	7.83E-09		*AKIRIN2*	
19	rs535440	34694581	cg21245903	34711622	9.97E-09		*LSM14A*	
19	rs1260634	34701331	cg21245903	34711622	1.00E-08		*LSM14A*	
19	rs668933	34704936	cg21245903	34711622	1.04E-08		*LSM14A*	
6	rs59113552	88236233	cg05834092	87792915	1.63E-08		*AKIRIN2*	
6	rs151077971	88405605	cg15059496	88185750	3.76E-08		*AKIRIN2*	
6	rs34472900	88405040	cg15059496	88185750	3.76E-08		*AKIRIN2*	
6	rs59113552	88236233	cg20648632	88182160	4.22E-08		*AKIRIN2*	
2	rs2090163	220178435	cg19020434	220199207	6.40E-08		*PTPRN*	
2	rs4674377	220201272	cg06895971	220147671	6.77E-08		*RESP18*	
6	rs59113552	88236233	cg15059496	88185750	7.55E-08		*AKIRIN2*	
2	rs4674377	220201272	cg19020434	220199207	7.84E-08		*RESP18*	
19	rs535440	34694581	cg01663383	34676533	8.98E-08		*LSM14A*	
19	rs1260634	34701331	cg01663383	34676533	9.01E-08		*LSM14A*	
19	rs668933	34704936	cg01663383	34676533	9.24E-08		*LSM14A*	

Gene: CpG site annotated gene. P DKD/eGFR: P-value for association between blood methylation at the CpG site and DKD (UK-ROI+FinnDiane EWAS) or with eGFR slope (CRIC EWAS).

ESM Table 12: Correlation between glomerular and tubular gene expression and glomerulosclerosis, fibrosis, and eGFR in nephrectomy samples. All associations with $p<0.05$ are shown, significant associations ($p<2.2\times10^{-4}$, corrected for 29 genes × 2 tissue compartments × 4 phenotypes) are in bold.

		eGFR		Glomerulosclerosis		Fibrosis		Group comparison	
**Gene**	**Tissue**	**r**	**p**	**r**	**p**	**r**	**p**	**Direction**	**p**
ALLC	glom								
ALLC	tub	0.26	***6.9E-08***			-0.50	***2.0E-16***	lowest in DKD	***8.3E-05***
COLEC11	glom	0.24	***1.0E-05***	-0.27	***1.8E-06***			lowest in DKD	***1.8E-05***
COLEC11	tub	0.21	***1.0E-05***			-0.47	***2.0E-16***	lowest in DKD	1.37E-03
PLEKHA7	glom	0.14	9.5E-03					lowest in DKD	***4.3E-06***
PLEKHA7	tub	0.30	***1.3E-10***			-0.49	***2.0E-16***	lowest in DKD	***1.3E-05***
SNX30	glom	0.24	***1.2E-05***	-0.22	***8.0E-05***			lowest for DKD	***5.5E-05***
SNX30	tub	0.35	***5.8E-14***			-0.56	***2.0E-16***		***1.2E-06***
DCLK1	glom								
DCLK1	tub	-0.15	1.48E-03			0.39	***7.4E-16***	Highest in DKD	***2.17E-04***
TENM2	glom	0.13	0.02	-0.18	1.7E-03				
TENM2	tub	0.27	***1.6E-08***			-0.29	***2.0E-09***	lowest in DKD	6.6E-04
COL4A3	glom	0.11	0.05	-0.16	4.8E-03				
COL4A3	tub					0.29	***3.2E-09***		
ZNF3	glom			0.12	0.04				
ZNF3	tub	-0.13	7.26E-03			0.26	***1.4E-07***		
TAMM41	glom	-0.11	0.04	0.16	4.1E-03				
TAMM41	tub	-0.20	***2.0E-05***			0.26	***1.5E-07***	highest in DKD	5.6E-03
AKIRIN2	glom								
AKIRIN2	tub					0.25	***2.8E-07***		
EIF4E	glom	-0.12	0.03					highest in DKD	***2.4E-06***
EIF4E	tub					-0.18	***1.9E-04***		
LSM14A	glom								
LSM14A	tub	0.22	***2.9E-06***			-0.13	0.01		
INIP	glom			-0.15	9.3E-03				
INIP	tub	0.22	***5.5E-06***			-0.21	***2.2E-05***		
MFF	glom								
MFF	tub					-0.21	***2.1E-05***		
MBLAC1	glom			-0.11	0.05			lowest in DKD	2.8E-03
MBLAC1	tub					-0.17	5.2E-04		
STAC	glom			-0.17	3.0E-03				
STAC	tub								

r: Pearson correlation coefficient between the phenotype and log2(Fragments per kilobases of transcript per 1 million mapped reads [FPKM]) of gene expression in glomeruli/tubules. p: p-value. Group comparison: ANOVA test for group comparison (Controls, chronic kidney disease [CKD], diabetic kidney disease [DKD], diabetes mellitus [DM] (without DKD), hypertension [HTN]) vs. log2(FPKM) gene expression.

ESM Table 13: Gene centric summary of the lead genes

				kidney eQTL	kidney mQTL	lead SNP PCHiC		Glom/ Tub eQTL	Nephrectomy Gene expression vs. phenotype correlations								NephroSeq DN vs. healthy					
									eGFR		Glomerulosc lerosis		fibrosis		Group comparison		Woroniecka DN vs healthy		Ju DN vs. healthy			
GENE	Pheno	Indication	PoPS	P min	P min	Max score		P min	r	p	r	p	r	p	direction	p	P	FC	P	FC	Pima BX1 correlations	Pima BX2 correlations
TENM2	CKD+DKD	Underlying lead SNP	***Yes***			***8.61***	***glom***		0.1	0.022	-0.2	0.002										
							tub		0.3	***1.6E-8***			-0.3	***2.0E-9***	lowest in DKD	6.6E-4						
DCLK1	ESRD vs. macro	Gene-based test			***6.81E-22***		***glom***												**1.2E-4**	**1.98**	ACR: r=0.29 p=0.016; MesVol: r=0.25 p=0.041;	GlomWidth: r=0.32 p=0.024; MesVol: r=0.29 p=0.038; FootProcW: r=0.71 p=0.0067;
							tub	0.035	-0.2	0.001			0.4	***7.4E-16***	Highest in DKD	***2.17E-4***	0.003	2.09	0.001	1.32	ACR: r=0.39 p=0.0069; **Fibrosis: r=0.52 p=0.0003;**	ACR: r=0.28 p=0.04; Fibrosis: r=0.36 p=0.015;
COL4A3	DKD	Missense variant	***Yes***			***8.89***	***glom***		0.1	0.047	-0.2	0.005					**8.3E-5**	-1.48	0.032	-0.43	SV: r=-0.28 p=0.024;	
							tub						0.3	***3.2E-09***					**2.1E-4**	-1.33		
COLEC11	CKD	Nearby				***9.67***	***glom***		0.2	***1.0E-5***	-0.3	***1.8E-6***			lowest in DKD	***1.8E-5***			0.019	**-1.59**		
							tub		0.2	***1.0E-5***			-0.5	***2.0E-16***	lowest in DKD	0.001			0.036	-1.47		ACR: r=-0.32 p=0.016; HbA1c: r=-0.29 p=0.034;
ALLC	CKD	Underlying lead SNP				***10.42***	***glom***												0.04	-1.19	GlomWidth: r=0.24 p=0.054; ΔSV: r=-0.46 p=0.01; HbA1c: r=0.3 p=0.041;	MesVol: r=-0.29 p=0.04;
							tub		0.3	***6.9E-8***			-0.5	***2.0E-16***	lowest in DKD	***8.3E-5***	**3.1E-4**	-1.43				
PLEKHA7	Micro	Underlying lead SNP	***Yes***			***9.49***	***glom***		0.1	9.5E-3					lowest in DKD	***4.3E-6***						
							tub		0.3	***1.3E-10***			-0.5	***2.0E-16***	lowest in DKD	***1.3E-5***						
SNX30	DKD	Gene-based test; kidney eQTL		**4.6E-7**			***glom***	0.012	0.2	***1.2E-05***	-0.2	***8.0E-5***			lowest in DKD	***5.5E-5***						
							tub	0.002	0.4	***5.8E-14***			-0.6	***2.0E-16***	lowest in DKD	***1.2E-6***						eGFR: r=0.33 p=0.013;
AKIRIN2	Severe DKD	TWAS		0.017	***1.12E-11***		***glom***															
							tub	0.001					0.3	***2.8E-7***								
EIF4E	ESRD vs. macro	Gene-based test			***1.10E-11***		***glom***		-0.1	0.027					highest in DKD	***2.4E-6***	0.010	-1.88	0.027	-1.27	ACR: r=0.25 p=0.041;	
							tub						-0.2	***1.9E-4***								eGFR: r=-0.4 p=0.0028;
MFF	Severe DKD	Gene-based test		0.032		***10.19***	***glom***	0.007									5.4E-4	-1.40			ΔMesVol: r=-0.46 p=0.011; ΔeGFR: r=0.43 p=0.017; Progr to ESRD: p=0.007;	
							tub	0.017					-0.2	***2.1E-5***								
ZNF3	Micro	Underlying tag SNP					***glom***				0.1	0.038									GlomVol: r=0.25 p=0.05; HbA1c: r=-0.25 p=0.043;	
							tub		-0.1	0.007			0.3	***1.4E-7***			**1.1E-4**	-1.33				
TAMM41	Micro	Nearby				***10.65***	***glom***		-0.1	0.037	0.2	0.004										
							tub		-0.2	***2.0E-5***			0.3	***1.5E-7***	highest in DKD	0.006						ACR: r=0.34 p=0.011;
LSM14A	Severe DKD	Gene-based test			***1.9E-28***		***glom***	0.004									7.4E-4	-1.27	0.001	-1.27	**ΔMesVol: r=-0.59 p=0.00065;** ΔSV: r=0.5 p=0.0052;	
							tub	0.017	0.2	***2.9E-6***			-0.1	0.011								
STAC	ESRD vs. All	Underlying lead SNP				***10.87***	***glom***				-0.2	0.003					0.030	-1.53	0.013	-1.38		
							tub										0.018	-1.18				

PTPRN	CKD	Gene-based test		0.007	***9.2E-12***		***glom***						0.016 1.14 0.002 -1.16	FootProcW: r=0.29 p=0.018;	
							tub						0.011 -1.21 9.5E-4 -1.11	HbA1c: r=0.28 p=0.059;	
INIP	DKD	Gene-based test			***2.2E-11***		***glom***	0.004		-0.1 0.009					
							tub		0.2 ***5.5E-6***		-0.2 ***2.2E-5***				
CNTN6	ESRD	PoPS	***Yes***	0.046			***glom***								
							tub								
MBLAC1	Micro	Nearby				***TSS***	***glom***			-0.1 0.054		lowest in DKD 0.003			
							tub				-0.2 5.2E-4				
COL20A1	CKD extremes	Gene-based test			***1.8E-12***		***glom***								FootProcW: r=-0.68 p=0.01;
							tub	0.018							
DCLK3	ESRD vs. All	Nearby				***8.8***	***glom***								GlomWidth: r=0.33 p=0.021; MesVol: r=0.34 p=0.015; GlomVol: r=0.42 p=0.0072; SV: r=-0.28 p=0.05;
							tub								
MUC7	Severe DKD	Nearby				***10.53***	***glom***								
							tub								
RESP18	CKD	Gene-based test			***2.7E-10***		***glom***								
							tub								
AMTN	Severe DKD	Nearby					***glom***								
							tub								
GPR158	Severe DKD	Gene-based test					***glom***								
							tub								
LINC01266	ESRD	Underlying lead SNP					***glom***								
							tub								
PRNCR1	ESRD vs. macro	Underlying lead SNP					***glom***								FootProcW: r=0.62 p=0.023;
							tub								
TENM2-AS1	CKD+DKD	lead SNP kidney eQTL		0.007	***NA***		***glom***		***NA***	***NA***	***NA***	***NA***			
							tub		***NA***	***NA***	***NA***	***NA***			
ADH4	ESRD vs. macro	glom eQTL for EIF4E		0.008	***NA***		***glom***	**2.1E-7**	***NA***	***NA***	***NA***	***NA***			
							tub	0.002	***NA***	***NA***	***NA***	***NA***			ACR: r=0.39 p=0.003;
SMIM8	Severe DKD	tub eQTL for AKIRIN2		1.1E-4	***NA***		***glom***		***NA***	***NA***	***NA***	***NA***			
							tub	**5.2E-5**	***NA***	***NA***	***NA***	***NA***			
CHRNA4	CKD extremes	kidney eQTL for COL20A1		**5.8E-5**	***NA***		***glom***	0.009	***NA***	***NA***	***NA***	***NA***	0.030 1.17	FootProcW: r=0.27 p=0.029;	GlomWidth: r=-0.29 p=0.041;
							tub	0.007	***NA***	***NA***	***NA***	***NA***			eGFR: r=0.31 p=0.024;

Indication: why gene was listed as a lead gene. PoPS: was prioritized by PoPS? Kidney eQTL: minimum P-value for eQTL association between the lead SNPs and the gene in the kidney eQTL meta-analysis. Kidney mQTL: minimum P-value for kidney methylation, between the lead SNPs and the gene, as assigned in the mQTL annotation. Lead SNP PCHiC: highest score for chromatin 3D conformation capture data at the chicp.org for the GWAS meta-analysis lead loci. Glom/Tub eQTL: minimum P-value for eQTL association between the lead SNPs and the gene in glomerular/ tubular eQTL data. Nephrectomy Gene expression vs. phenotype correlations: Pearson correlation (r) and p-value for correlation between glomerular/tubular gene expression and the phenotype. NephroSeq DN vs. healthy: Fold change (FC) and p-value for differential glomerular/tubular gene expression in DN vs. healthy in the Woroniecka and Ju CKD data sets. Pima BX1/BX2 correlations: Pearson correlation coefficient r and p-value for glomerular/tubular gene expression vs. morphological parameters at the first (BX1) or second (BX2) biopsy.

ESM Table 14: Mendelian Randomization (MR) results for DKD (All vs Ctrl)

Exposure	method	nsnp	OR (95%CI)	se	p-val	Q-pval	I^2 (%)
**Body fat**	Inverse variance weighted	9	1.48 (0.73-2.98)	0.36	0.27	0.02	60.2
**Body fat**	Weighted median	9	1.26 (0.64-2.49)	0.35	0.50	NA	NA
**Body fat**	MR Egger	9	1.04 (0.01-98.21)	2.32	0.99	0.01	65.1
**Body mass index**	Inverse variance weighted	78	1.86 (1.55-2.23)	0.09	2.56e-11	0.50	0.00
**Body mass index**	Weighted median	78	1.76 (1.31-2.36)	0.15	1.85e-04	NA	NA
**Body mass index**	MR Egger	78	1.98 (1.28-3.08)	0.22	3.18e-03	0.48	0.36
**Obesity class 1**	Inverse variance weighted	17	1.28 (1.14-1.44)	0.06	1.90e-05	0.10	31.6
**Obesity class 1**	Weighted median	17	1.24 (1.08-1.42)	0.07	2.26e-03	NA	NA
**Obesity class 1**	MR Egger	17	1.21 (0.87-1.69)	0.17	0.28	0.08	35.2
**Obesity class 2**	Inverse variance weighted	11	1.16 (1.05-1.28)	0.05	2.83e-03	0.10	37.2
**Obesity class 2**	Weighted median	11	1.13 (1.02-1.26)	0.05	0.02	NA	NA
**Obesity class 2**	MR Egger	11	1.21 (0.89-1.65)	0.16	0.25	0.07	42.9
**Overweight**	Inverse variance weighted	14	1.47 (1.22-1.77)	0.09	4.51e-05	0.07	38.6
**Overweight**	Weighted median	14	1.31 (1.05-1.64)	0.11	0.02	NA	NA
**Overweight**	MR Egger	14	1.11 (0.59-2.08)	0.32	0.75	0.07	39.3
**Hip circumference**	Inverse variance weighted	49	1.74 (1.34-2.25)	0.13	2.68e-05	0.07	24.5
**Hip circumference**	Weighted median	49	1.89 (1.33-2.67)	0.18	3.29e-04	NA	NA
**Hip circumference**	MR Egger	49	3.45 (1.35-8.77)	0.48	0.01	0.09	22.5
**Waist circumference**	Inverse variance weighted	45	1.90 (1.49-2.42)	0.12	1.71e-07	0.28	10.0
**Waist circumference**	Weighted median	45	1.86 (1.29-2.67)	0.19	8.35e-04	NA	NA
**Waist circumference**	MR Egger	45	2.39 (1.27-4.52)	0.32	0.01	0.27	10.8
**Waist-to-hip ratio**	Inverse variance weighted	30	1.34 (0.97-1.84)	0.16	0.08	0.43	2.5
**Waist-to-hip ratio**	Weighted median	30	1.15 (0.72-1.84)	0.24	0.55	NA	NA
**Waist-to-hip ratio**	MR Egger	30	1.61 (0.37-6.95)	0.75	0.53	0.38	5.7
**Coronary artery disease**	Inverse variance weighted	61	0.99 (0.91-1.08)	0.04	0.79	0.65	0.00
**Coronary artery disease**	Weighted median	61	0.95 (0.83-1.09)	0.07	0.47	NA	NA
**Coronary artery disease**	MR Egger	61	0.92 (0.77-1.09)	0.09	0.34	0.65	0.00
**Type 2 diabetes**	Inverse variance weighted	25	1.14 (1.02-1.27)	0.05	0.02	0.06	32.5
**Type 2 diabetes**	Weighted median	25	1.11 (0.97-1.27)	0.07	0.14	NA	NA
**Type 2 diabetes**	MR Egger	25	1.20 (0.79-1.83)	0.22	0.40	0.046	35.1
**HDL cholesterol**	Inverse variance weighted	84	0.91 (0.82-1.02)	0.06	0.11	0.73	0.00
**HDL cholesterol**	Weighted median	84	1.03 (0.87-1.21)	0.09	0.77	NA	NA
**HDL cholesterol**	MR Egger	84	1.16 (0.94-1.43)	0.11	0.17	0.87	0.00
**Urate**	Inverse variance weighted	25	1.07 (0.94-1.23)	0.07	0.31	0.11	26.8
**Urate**	Weighted median	25	1.08 (0.90-1.31)	0.10	0.40	NA	NA
**Urate**	MR Egger	25	0.90 (0.71-1.15)	0.12	0.41	0.17	21.3
**Smoking status: Never**	Inverse variance weighted	77	0.54 (0.27-1.06)	0.35	0.07	0.10	17.7
**Smoking status: Never**	Weighted median	77	0.84 (0.31-2.24)	0.50	0.72	NA	NA
**Smoking status: Never**	MR Egger	77	1.69 (0.08- 35.31)	1.55	0.74	0.09	18.2
**Smoking status: Current**	Inverse variance weighted	15	0.94 (0.10-8.84)	1.15	0.96	0.95	0
**Smoking status: Current**	Weighted median	15	0.24 (0.01-4.54)	1.50	0.34	NA	NA
**Smoking status: Current**	MR Egger	15	4.11 (0.00-1.4e5)	5.34	0.80	0.93	0

ESM Table 15. Egger intercepts for Mendelian Randomization analyses on DKD (all vs. Ctrl phenotype).

Exposure	Egger intercept	Intercept SE	Intercept p-value
**Body fat**	0.00094	0.051	0.99
**BMI**	-0.0020	0.0063	0.76
**Obesity class I**	0.0072	0.019	0.71
**Obesity class II**	-0.0083	0.027	0.77
**Overweight**	0.024	0.026	0.37
**Waist circumference**	-0.0095	0.011	0.39
**Hip circumference**	-0.0071	0.0093	0.45
**WHR**	0.0057	0.017	0.74
**CAD**	0.0086	0.0064	0.18
**Type 2 diabetes**	0.012	0.012	0.31
**HDL cholesterol**	-0.013	0.0047	0.0085
**Urate**	0.014	0.0090	0.12
**Ever smoking**	-0.0084	0.013	0.51
**Current smoking**	-0.0078	0.027	0.78

ESM Figure 1: Odds ratios required to detect association with ≥ 80% statistical power, in function of the variant minor allele frequency [MAF]. Power was calculated at MAFs of 0.1%, 1%, 5%, 10%, 20%, 30%, 40%, and 50%.

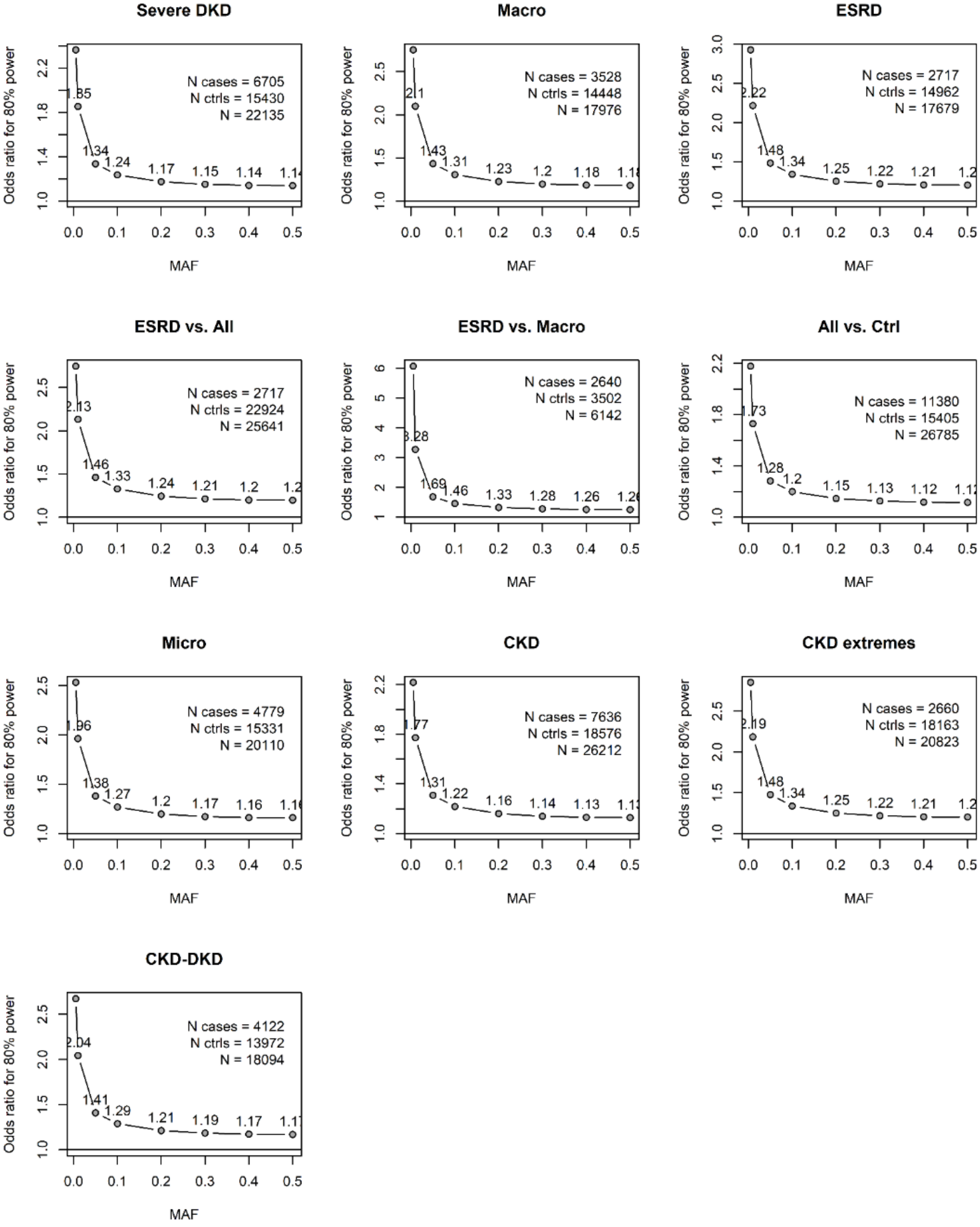

ESM Figure 2: Manhattan and QQ-plots of the ten DKD GWAS meta-analysis results.

A: Severe DKD (Macroalbuminuria or ESRD vs. normal AER). λ_{GC} = 1.029, LD score regression (LDSR) intercept = 1.019.

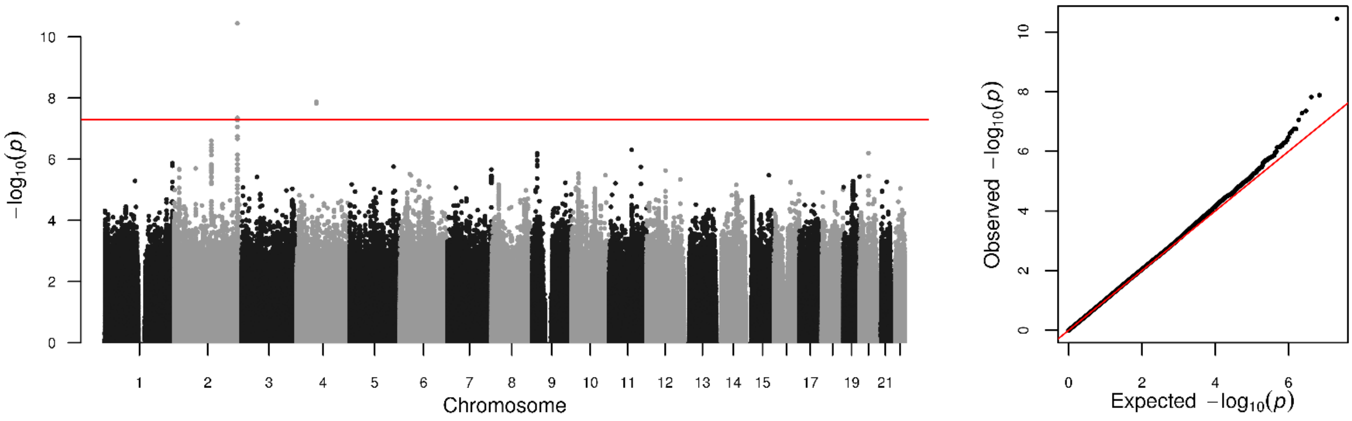

B: Macro (Macroalbuminuria vs. normal AER). λ_{GC} = 1.002, LDSR intercept = 1.028.

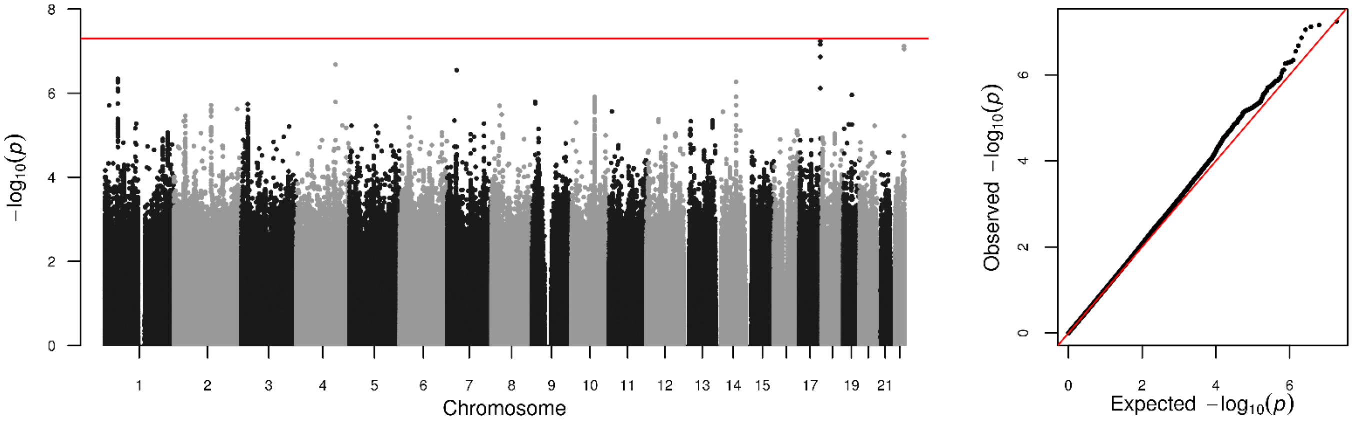

C: ESRD (ESRD vs. normal AER). λ_{GC} = 1.011, LDSR intercept = 1.018.

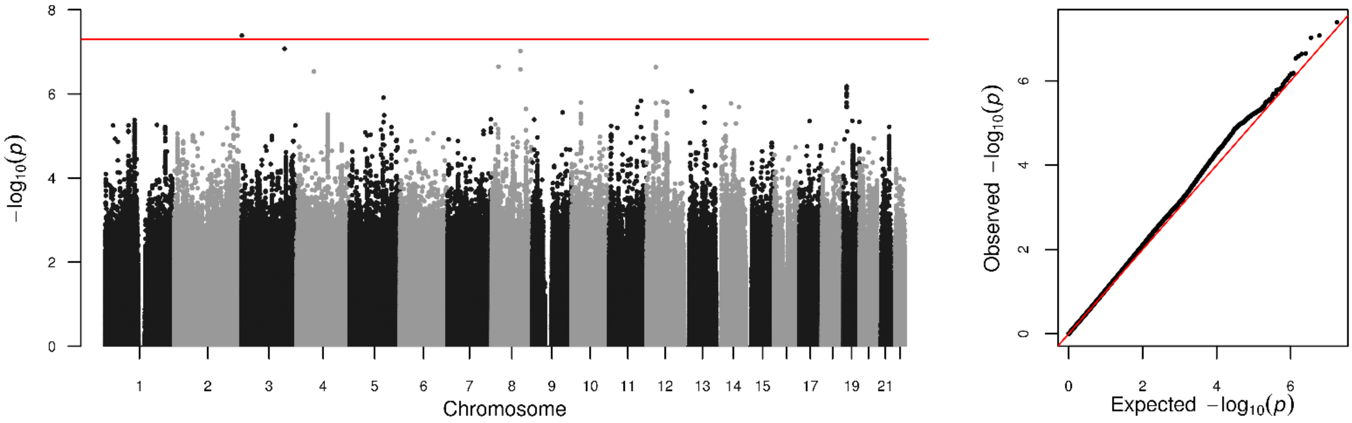

D: ESRD vs. All (ESRD vs. macro- or microalbuminuria or normal AER). λ_{GC} = 1.029, LDSR intercept = 1.054.

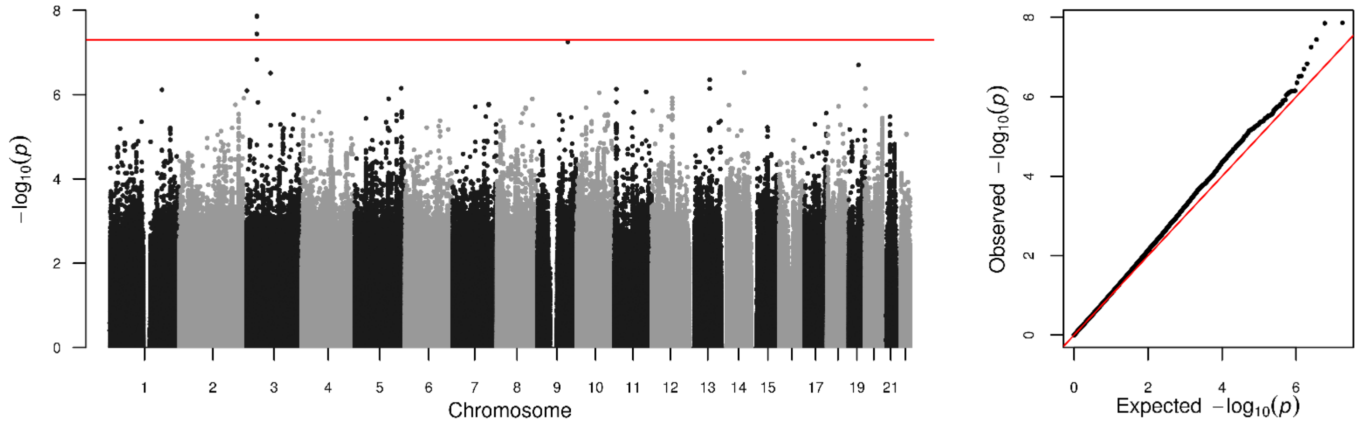

E: ESRD vs. Macro (ESRD vs. macroalbuminuria). λ_{GC} = 1.011, LDSR intercept = 1.009.

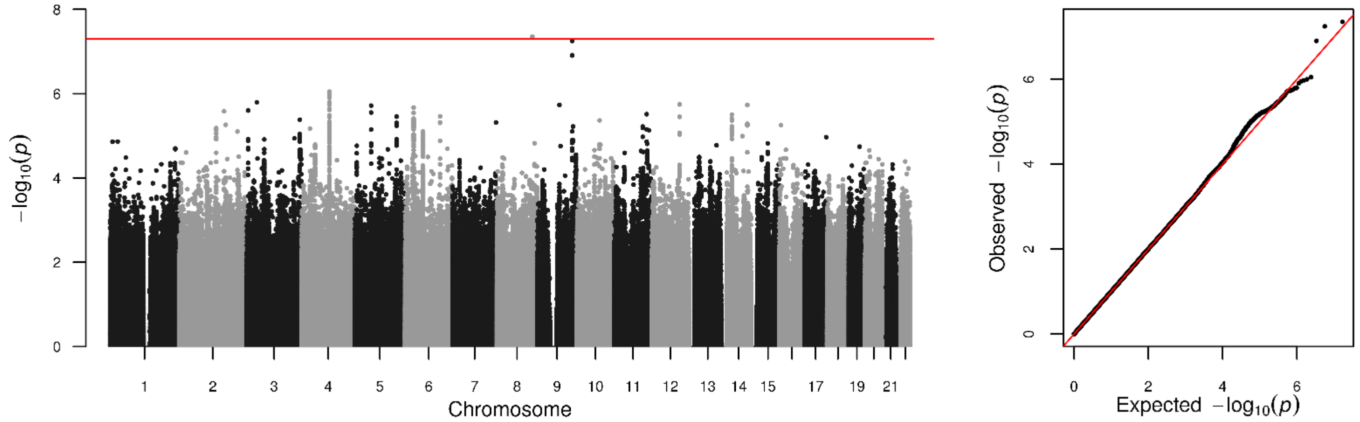

F: All vs. Ctrl (Micro- or Macroalbuminuria or ESRD vs. normal AER). λ_{GC} = 1.035, LDSR intercept = 1.005.

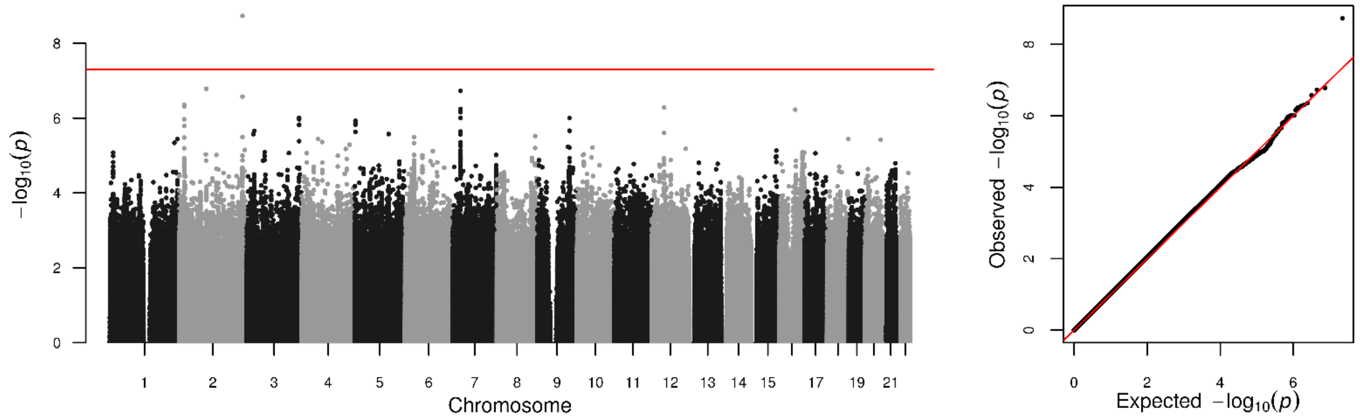

G: Micro (Microalbuminuria vs. normal AER). λ_{GC} = 1.008, LDSR intercept = 1.004.

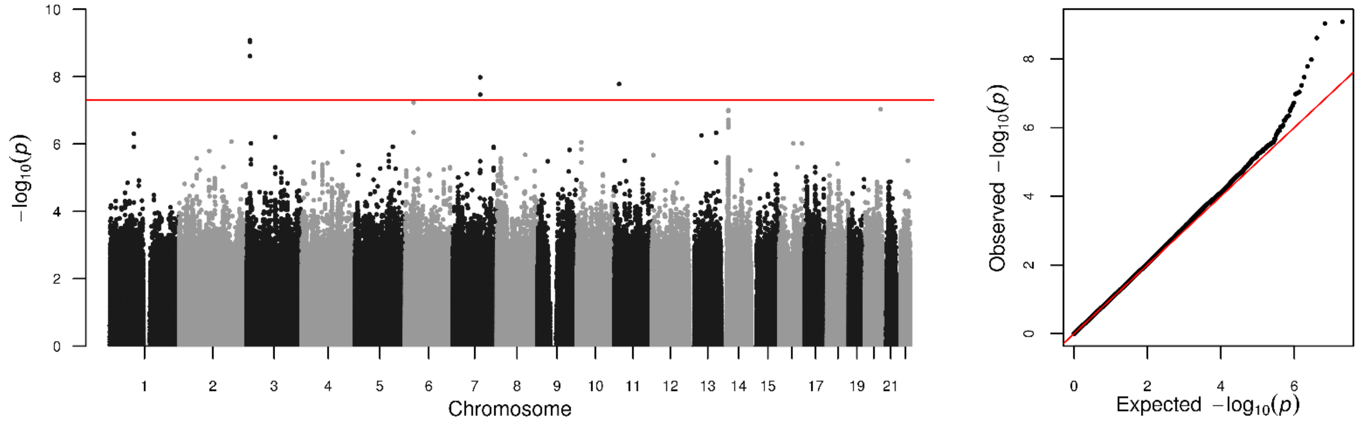

H: CKD (eGFR < 60 ml/min/1.73m^2 vs. eGFR ≥60 ml/min/1.73m^2). λ_{GC} = 1.041, LDSR intercept = 1.028.

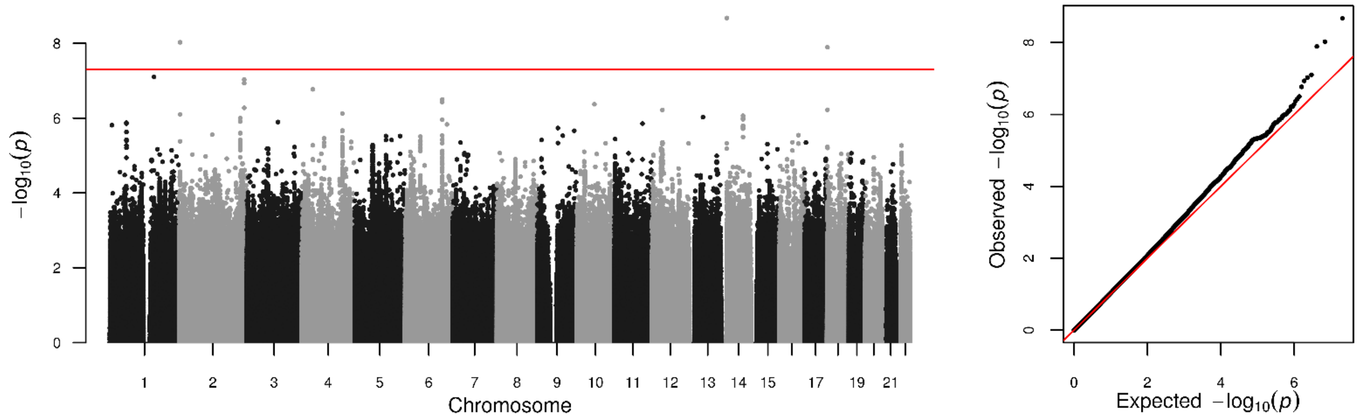

I: CKD Extremes (ESRD or eGFR < 15 ml/min/1.73m^2 vs. eGFR ≥ 60 ml/min/1.73m^2). λ_{GC} = 1.023, LDSR intercept = 1.019.

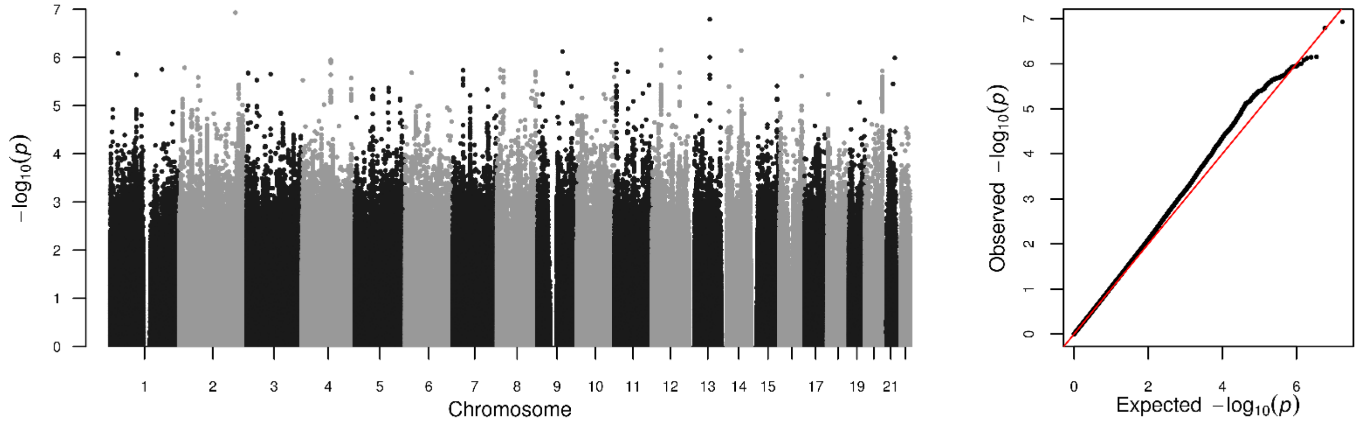

J: CKD-DKD (ESRD or eGFR < 45 ml/min/1.73m^2 and micro- or macroalbuminuria vs. eGFR ≥ 60ml/min/1.73m^2 and normal AER). λ_{GC} = 1.023, LDSR intercept = 1.031.

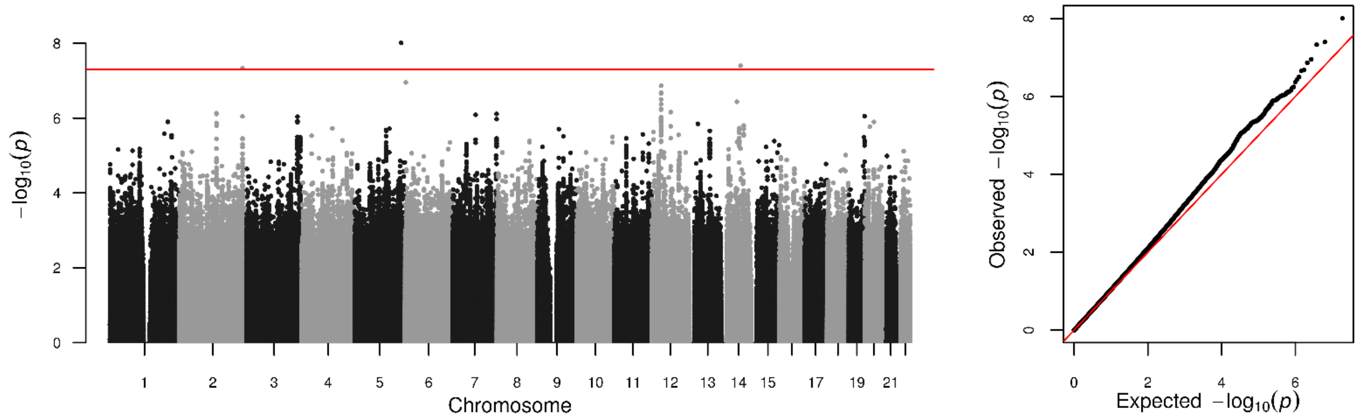

ESM Figure 3: Regional association plot for the *COL4A3* gene region associated with Severe DKD, indicating a secondary association peak at chr2:228259302 (rs6436688, effect allele (A) frequency 56%, OR = 1.13 (95% confidence interval 1.08 – 1.19), p-value 1.79×10^{-7}). SNP rs6436688 is in partial LD (D'=0.51, r^2=0.08, 1000Genomes European ancestry populations) with the original *COL4A3* lead variant rs55703767. Variants are colored according to their LD correlation with the primary signal (chr2:228121101 rs55703767) in red, or with the secondary peak in blue; stronger color indicates stronger correlation.

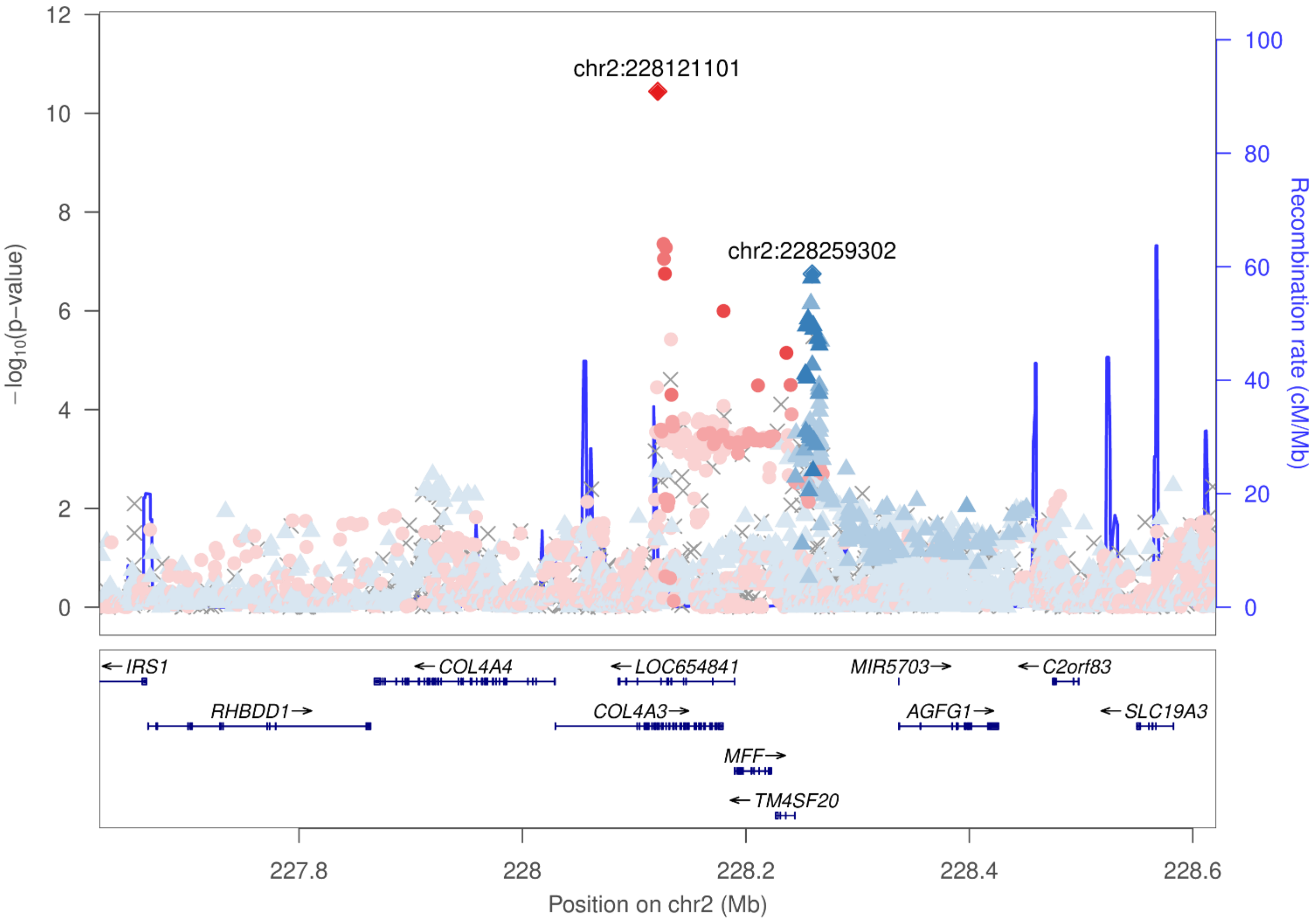

ESM Figure 4: Regional association plots for the GWAS meta-analysis lead loci (A-K).
A: CKD+DKD chr5:166978230 (rs72831309)

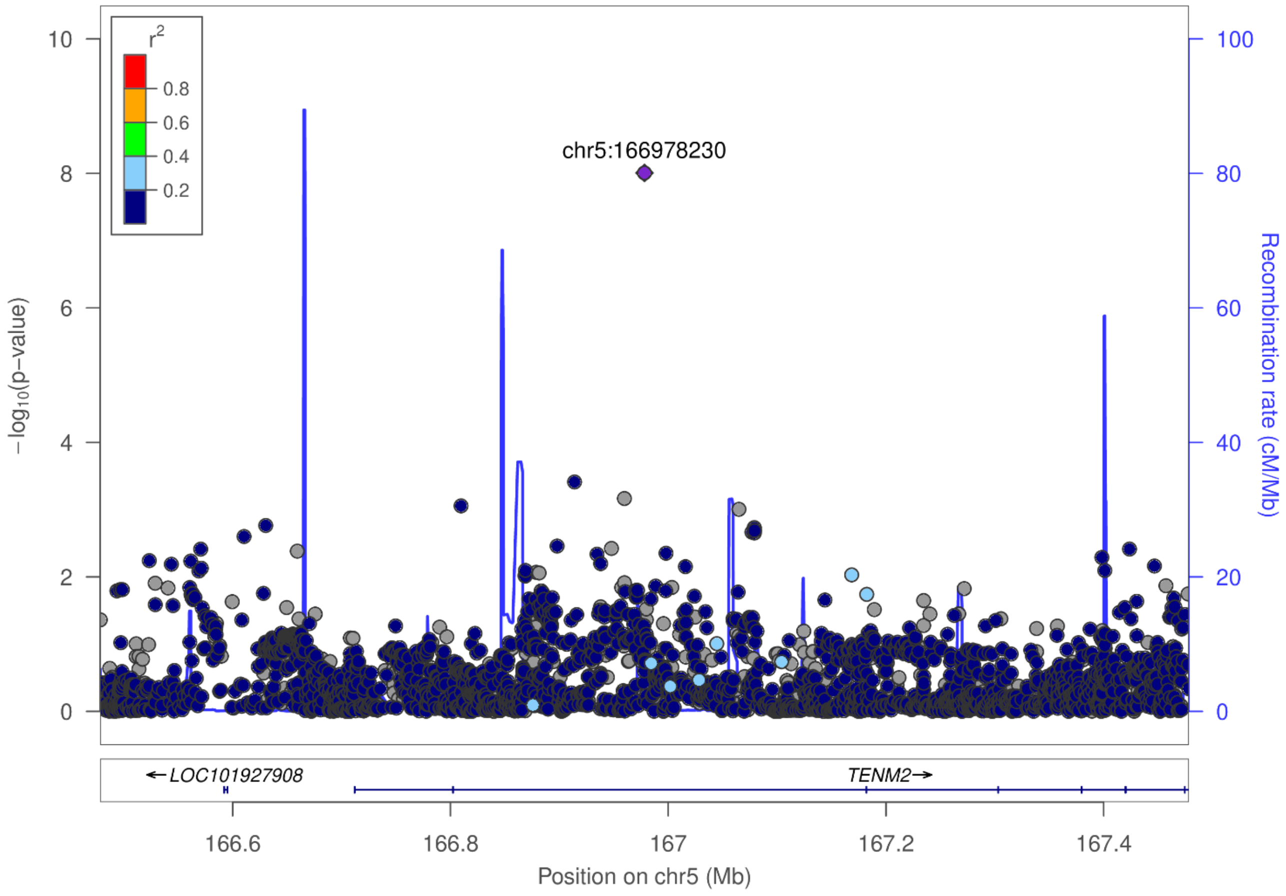

B: CKD chr2:3745215 (rs12615970)

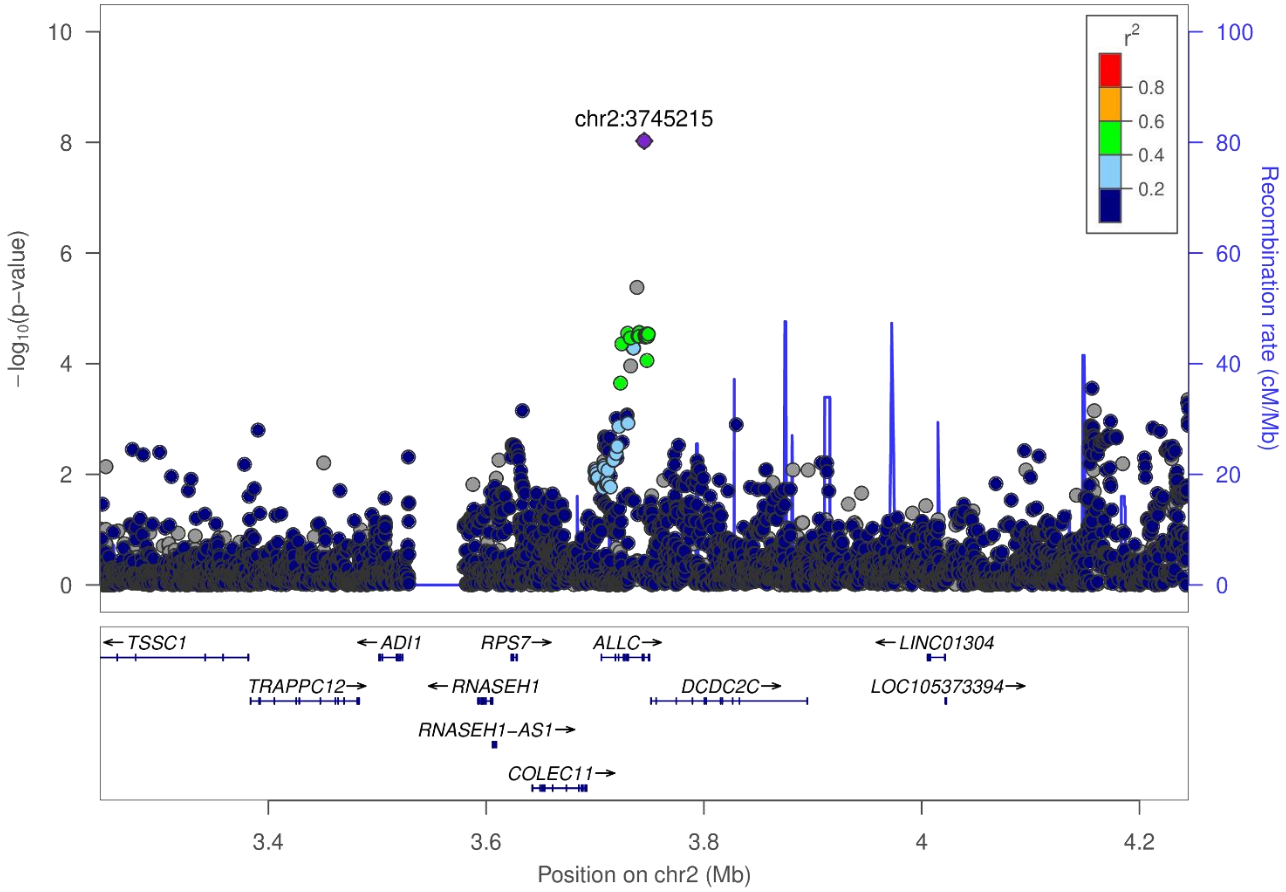

C: Severe DKD chr2:228121101 (rs55703767)

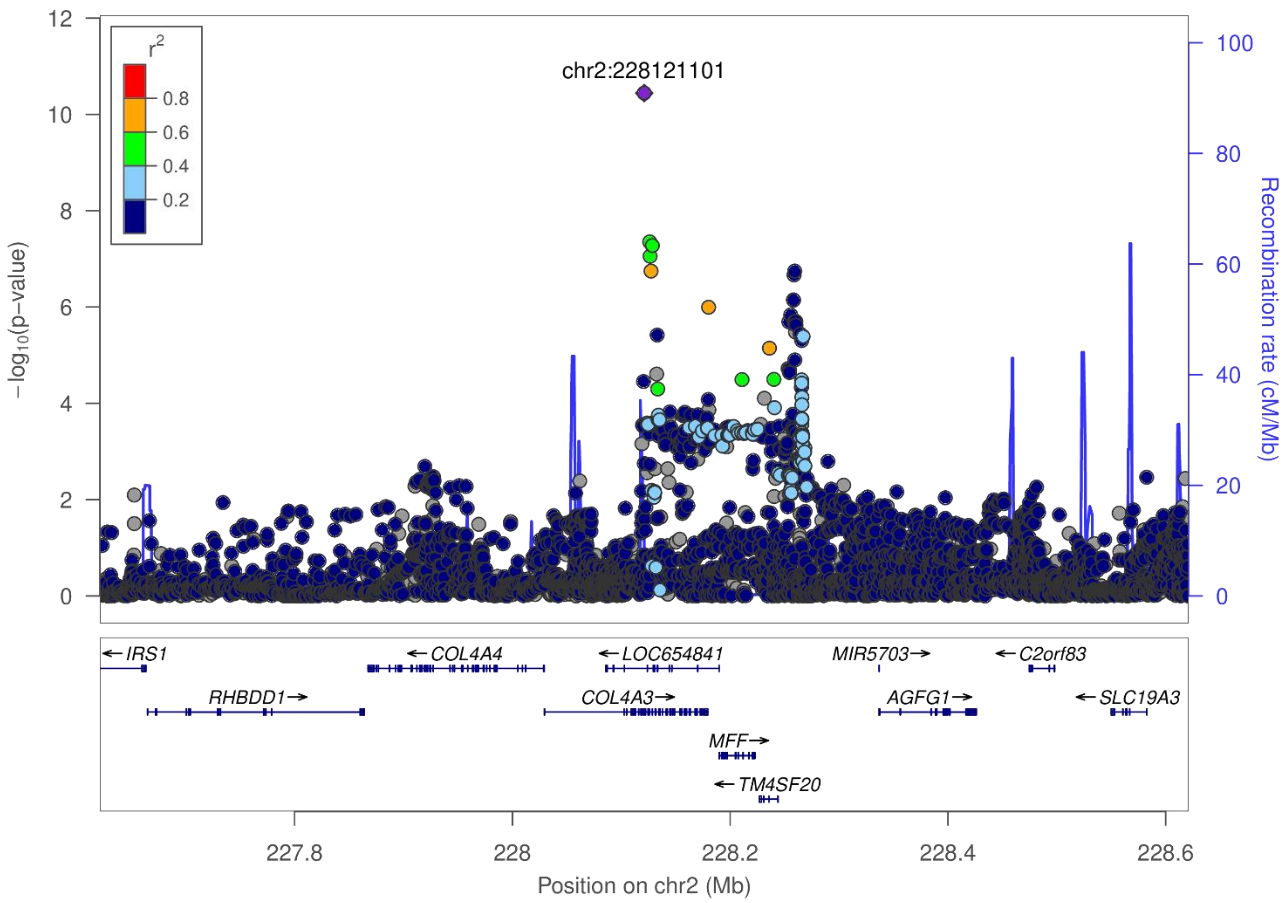

D: ESRD chr3:926345 (rs115061173)

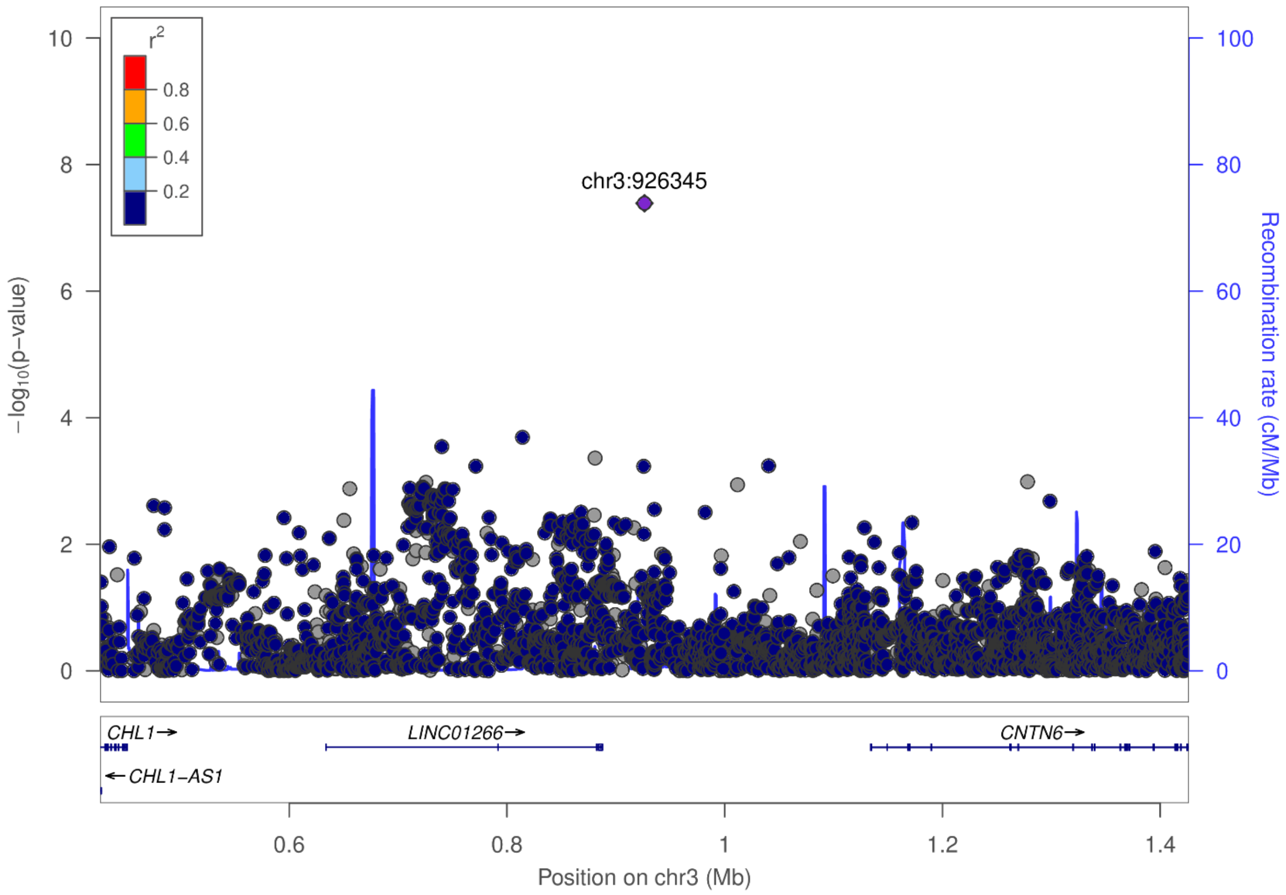

E: Micro chr3:11910635 (rs142823282)

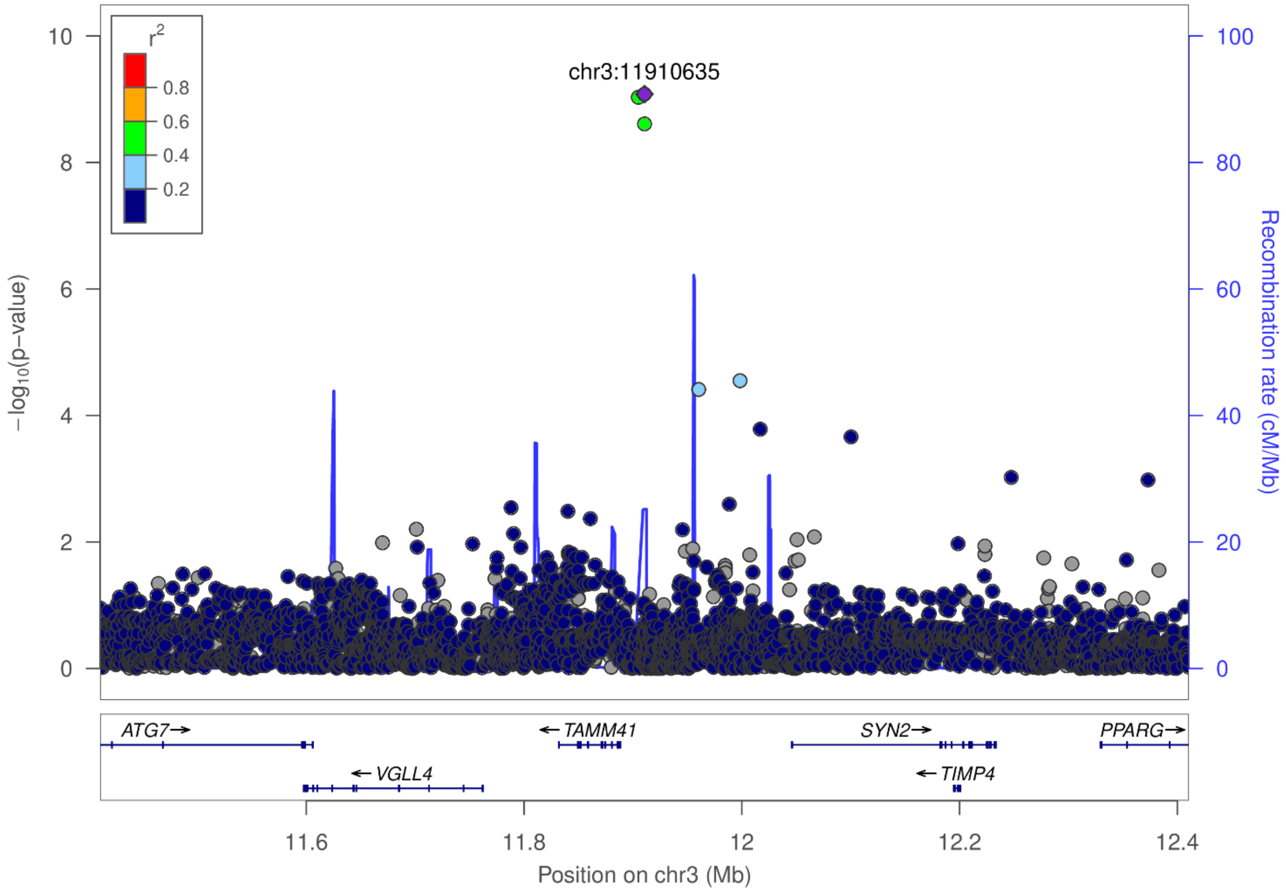

F: ESRD vs. All chr3:36566312 (rs116216059). The SNP rs116216059 is located on a single nucleus ATACseq (snATACseq) peak border in podocytes (PODO), peak value 1.1 (peak maximum value 7.0).

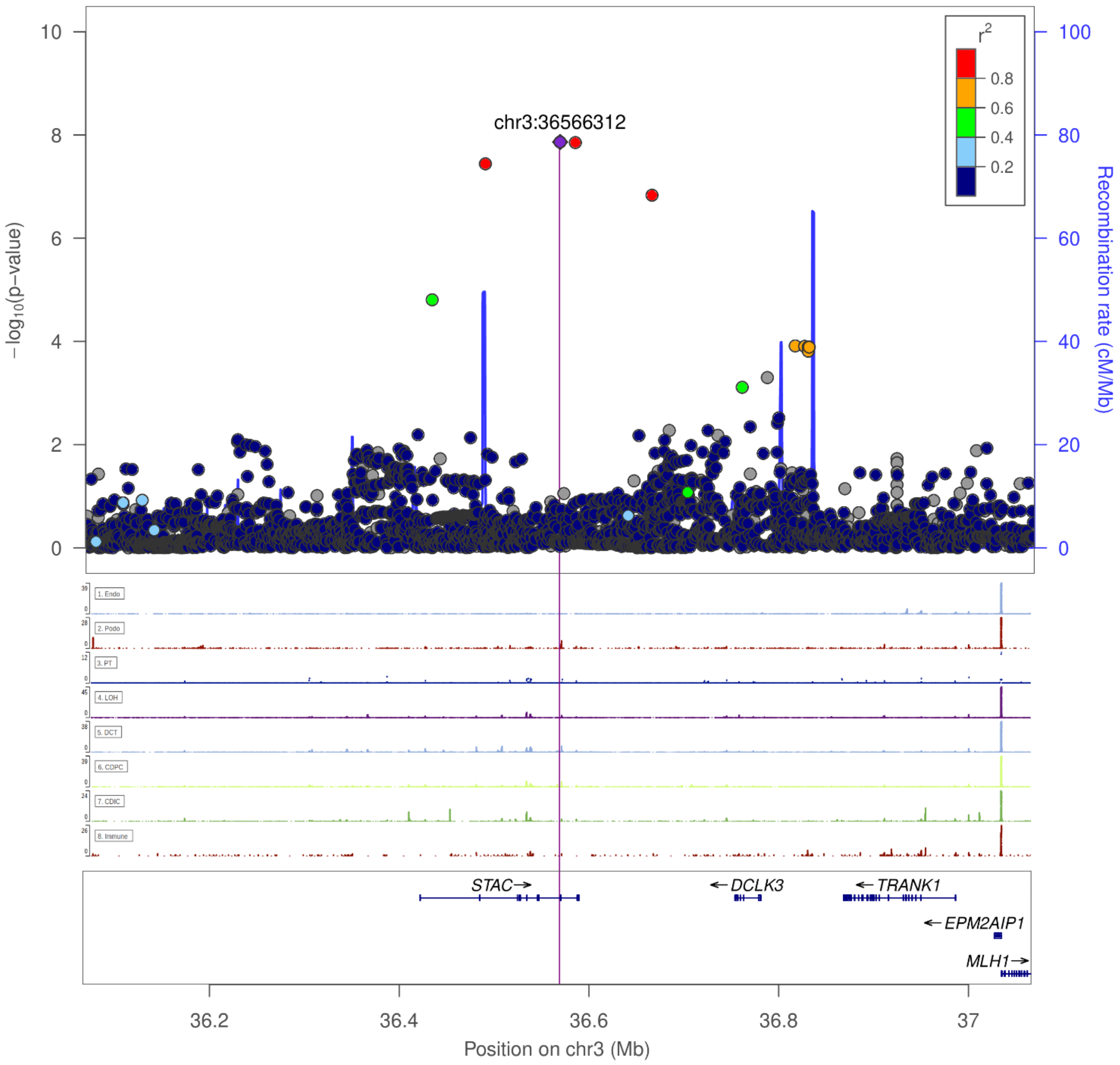

G: Severe DKD chr4:71358776 (rs191449639)

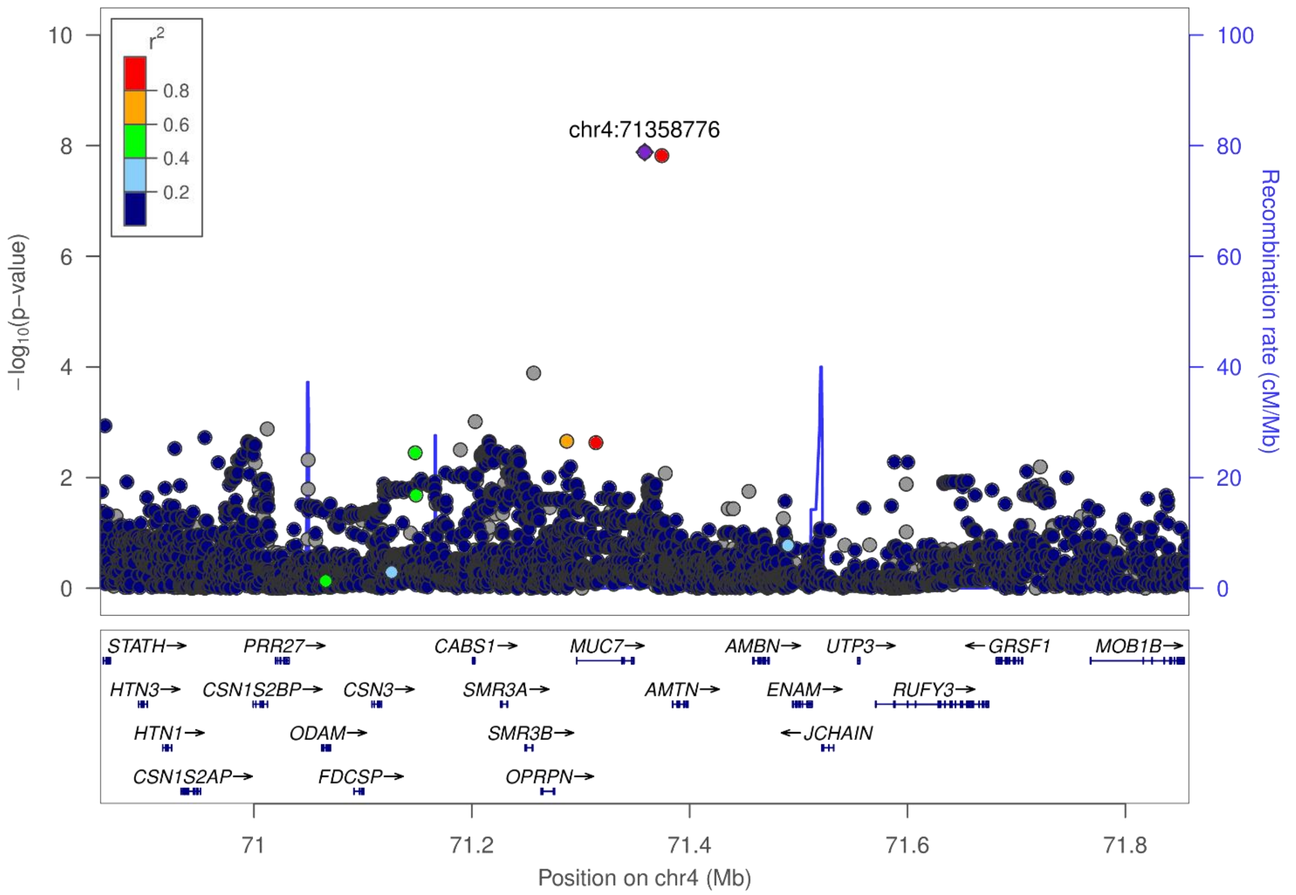

H: Micro chr7:99728546 (rs77273076)

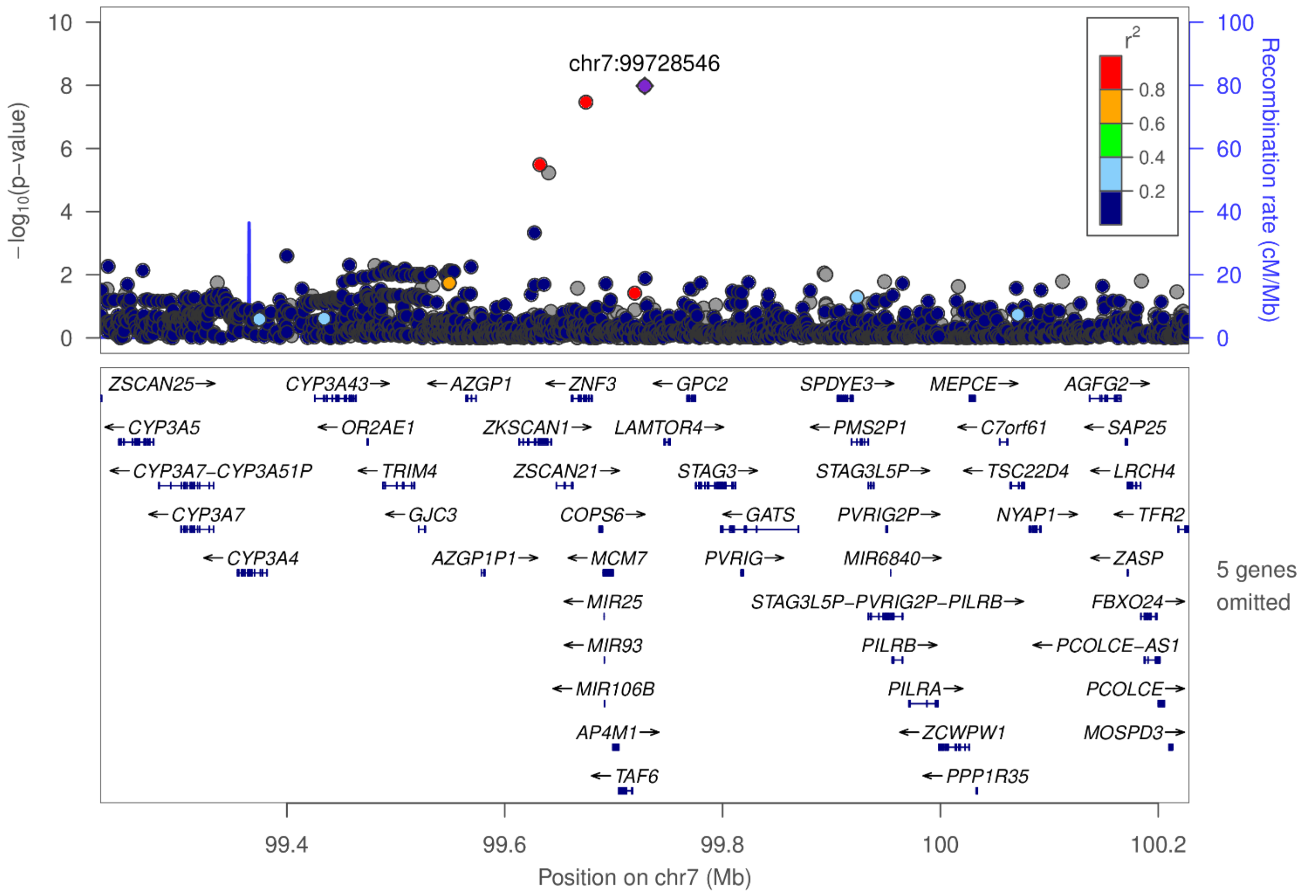

I: ESRD vs. macro chr8:128100029 (rs551191707)

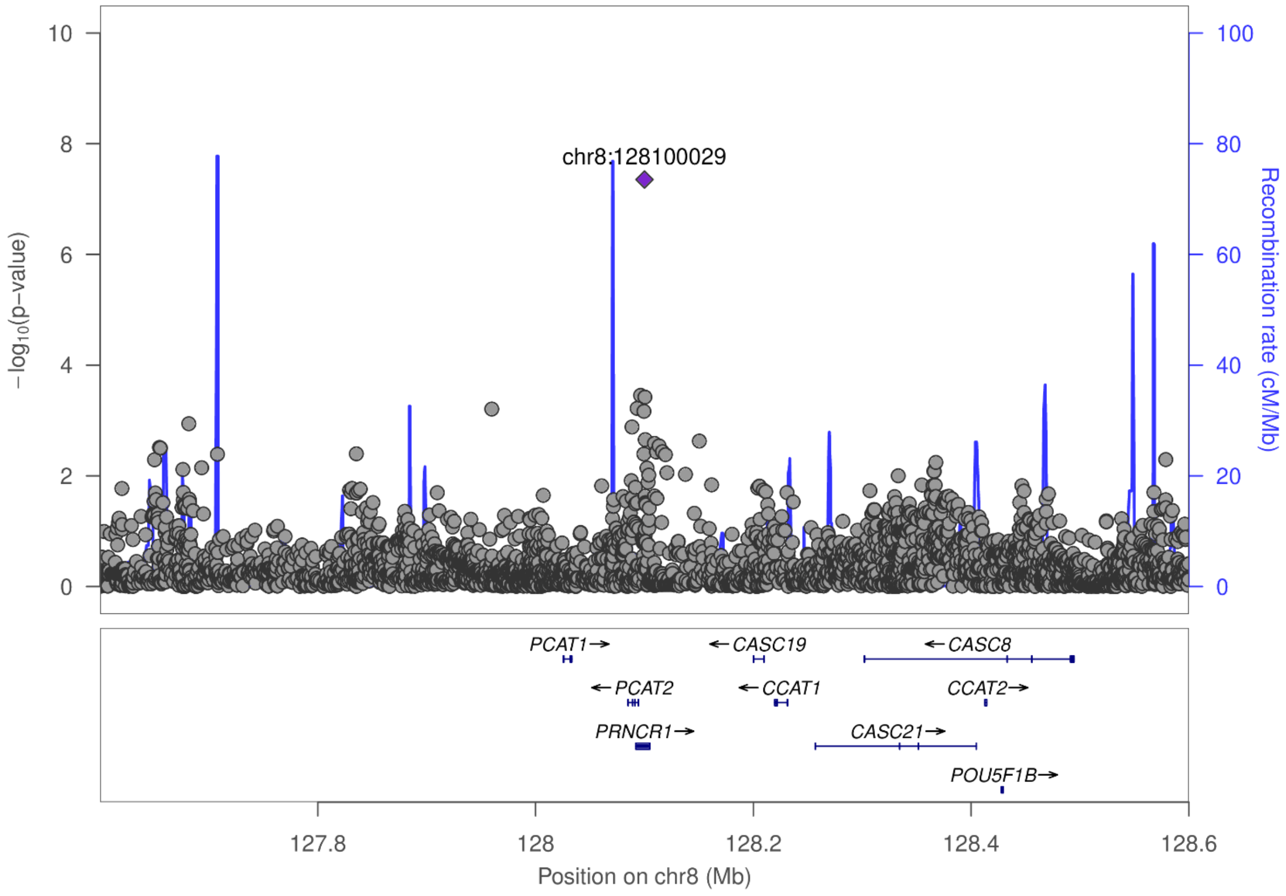

J: Micro chr11:16937846 (rs183937294)

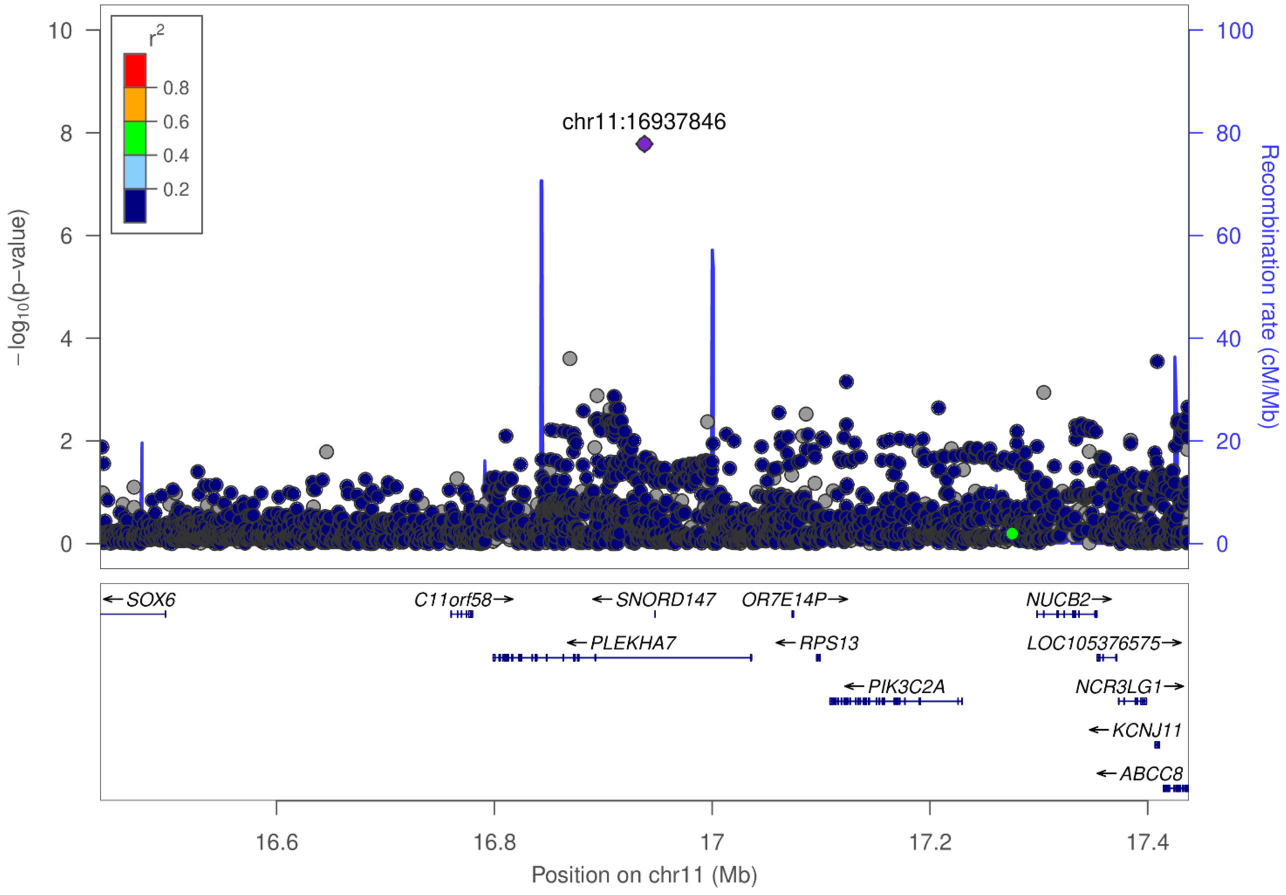

K: CKD chr18:1811108 (rs185299109)

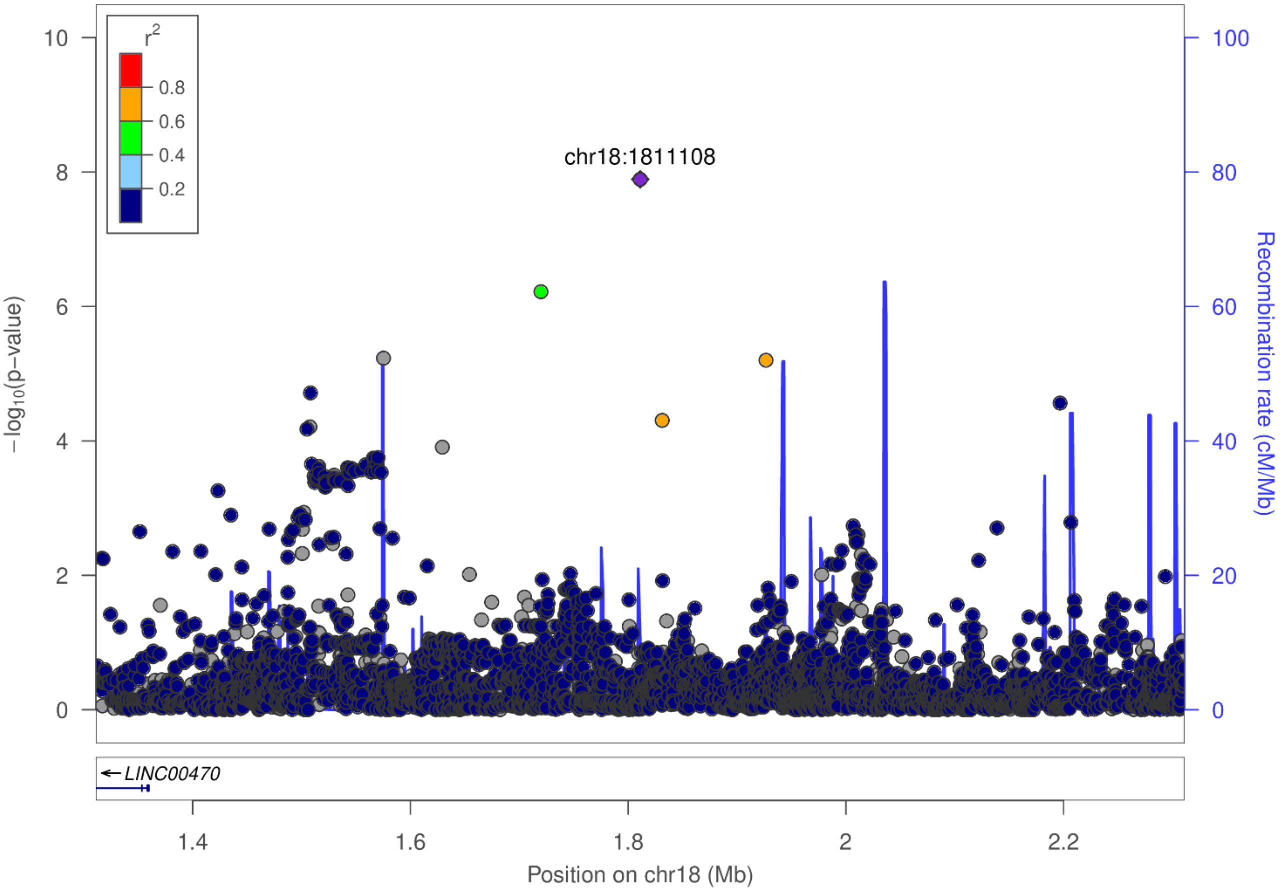

ESM Figure 5: Gene prioritization for the COL4A3 gene at lead SNP rs5570367 associated with Severe DKD using multiple intersecting gene prioritization approaches (PoPS, nearest gene, and MAGMA). Plotted is the PoP Score (y-axis) versus the genes within a 500kb flanking window surrounding the lead SNP (x-axis), colored by distance to the lead SNP, and bolded if the gene was also within the top 10% of prioritized genes genome-wide using MAGMA.

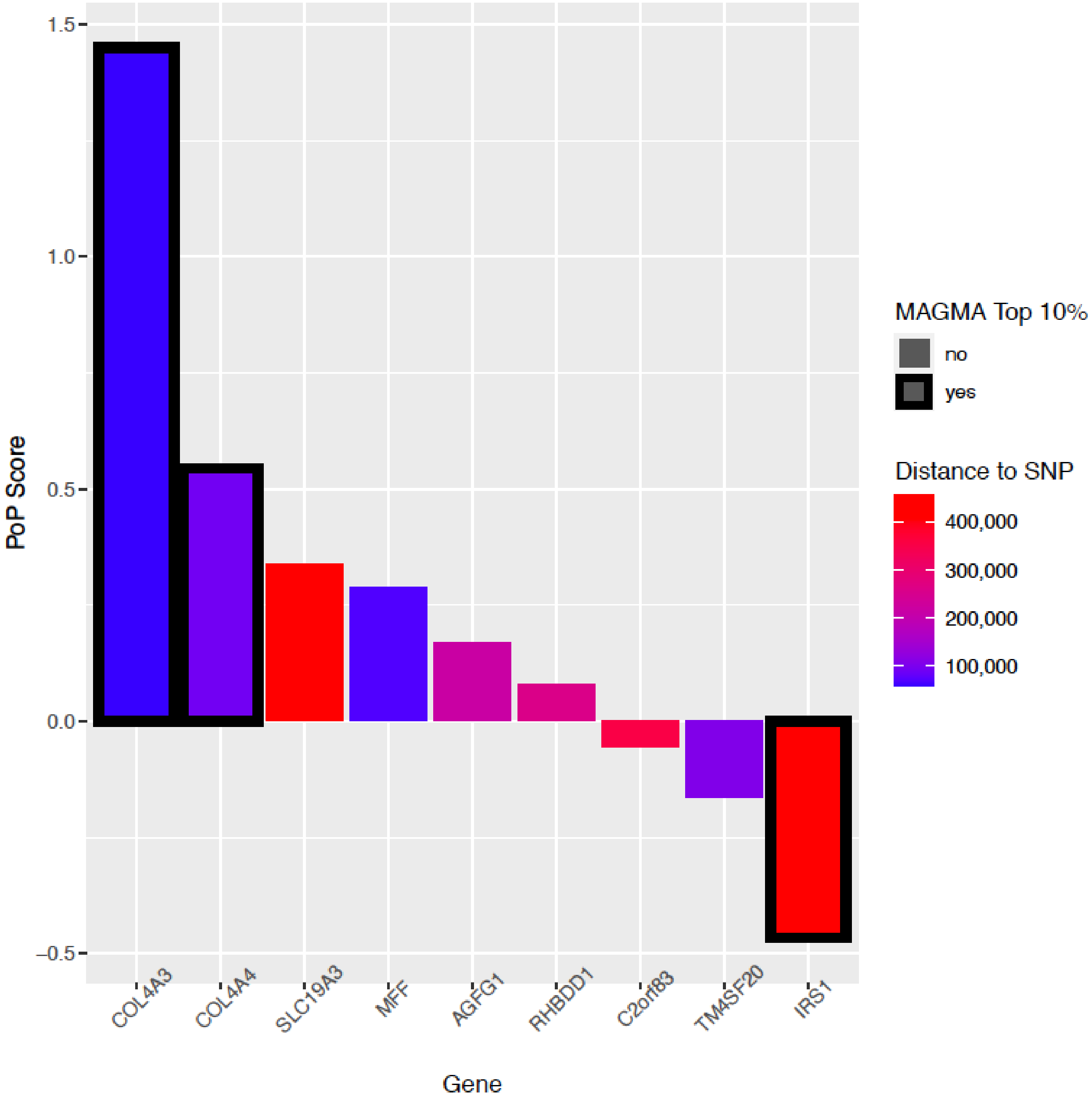

ESM Figure 6: Regional association plots for the gene-level analysis results from MAGMA and PASCAL analysis. The implicated gene region is highlighted in blue. If the same gene was significant in both analyses, only MAGMA region is highlighted (gene flanking ± 50 kbp, vs. ± 5 kbp for PASCAL).

A: CKD, *PTPRN*

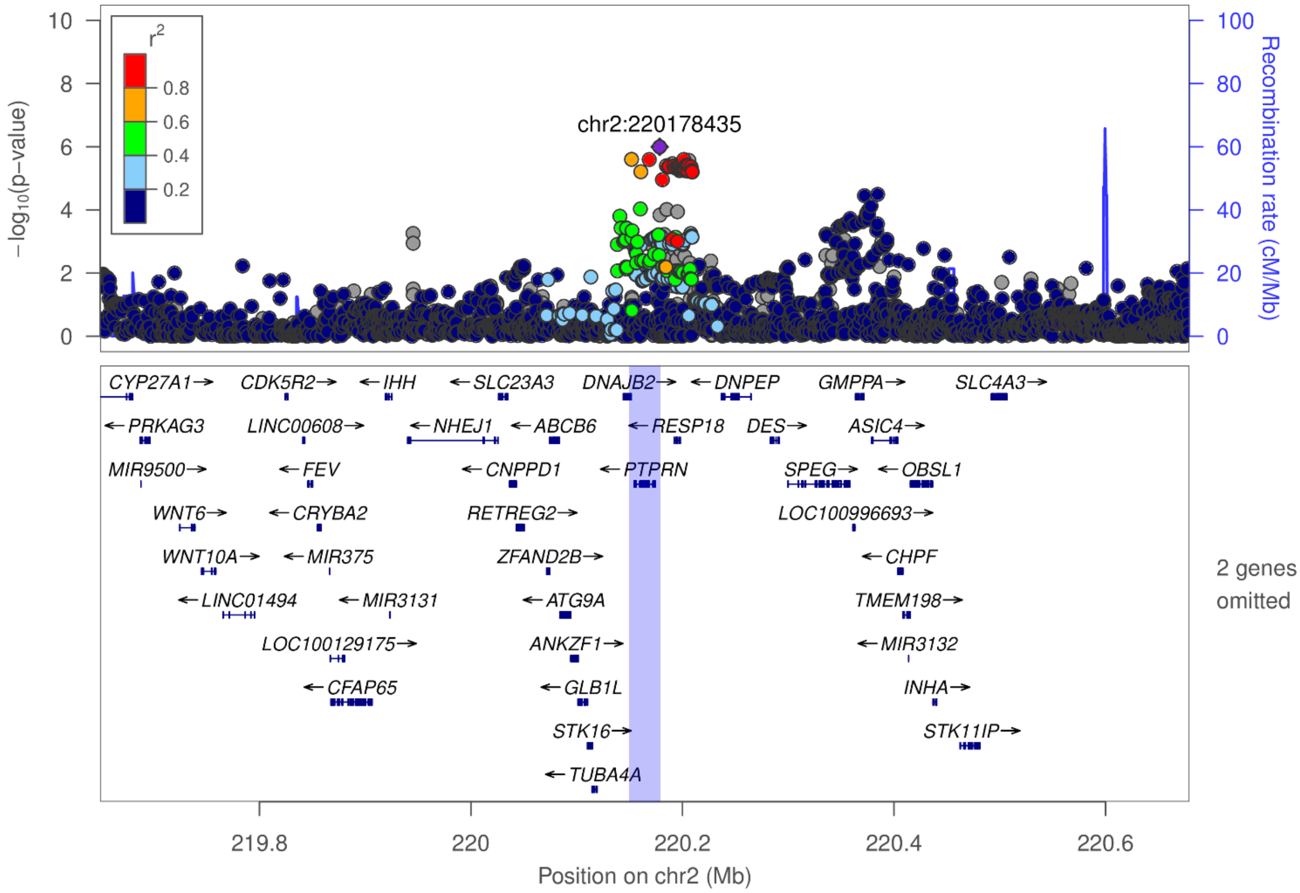

B: CKD, *RESP18*

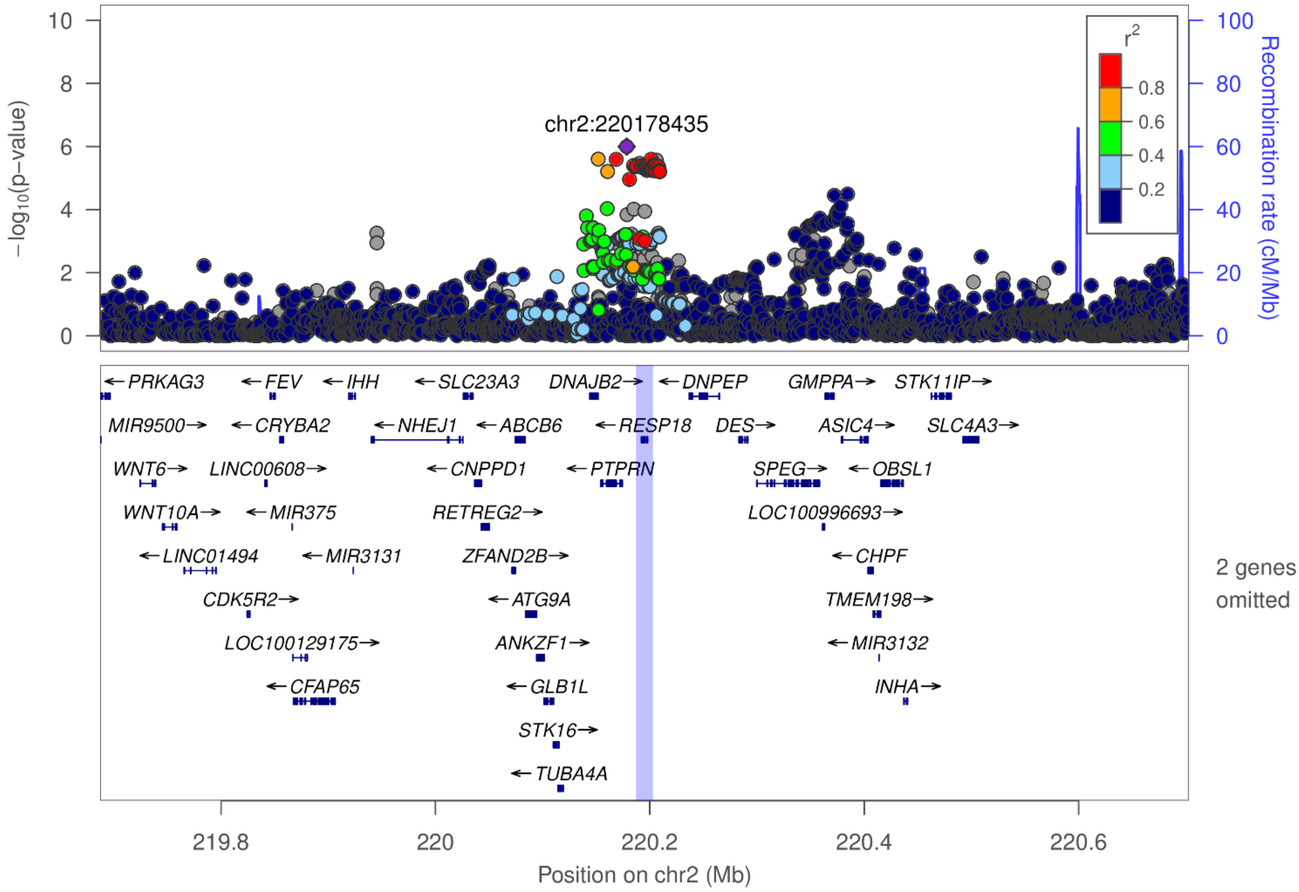

C: Severe DKD, *MFF*

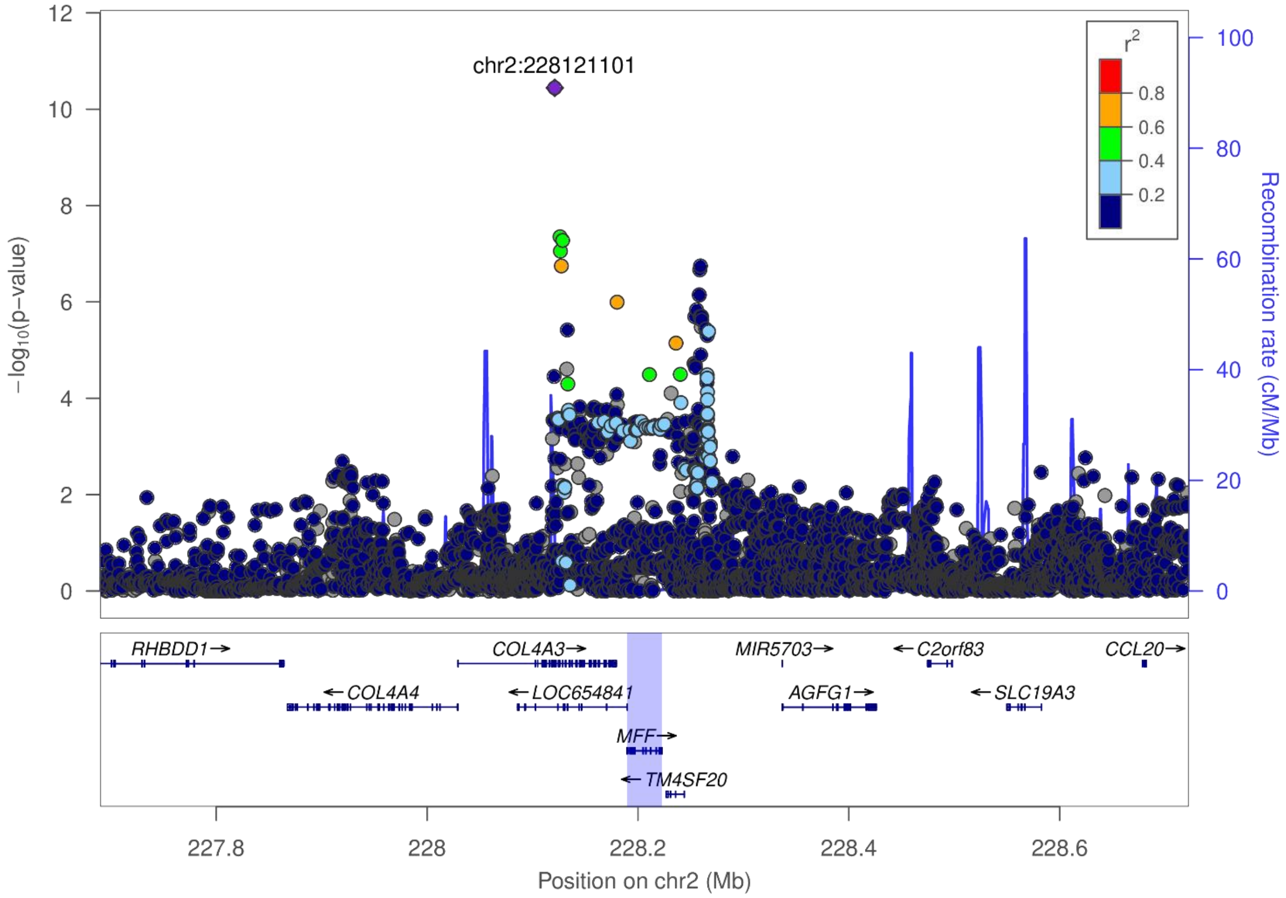

D: ESRD vs. macro, *EIF4E*

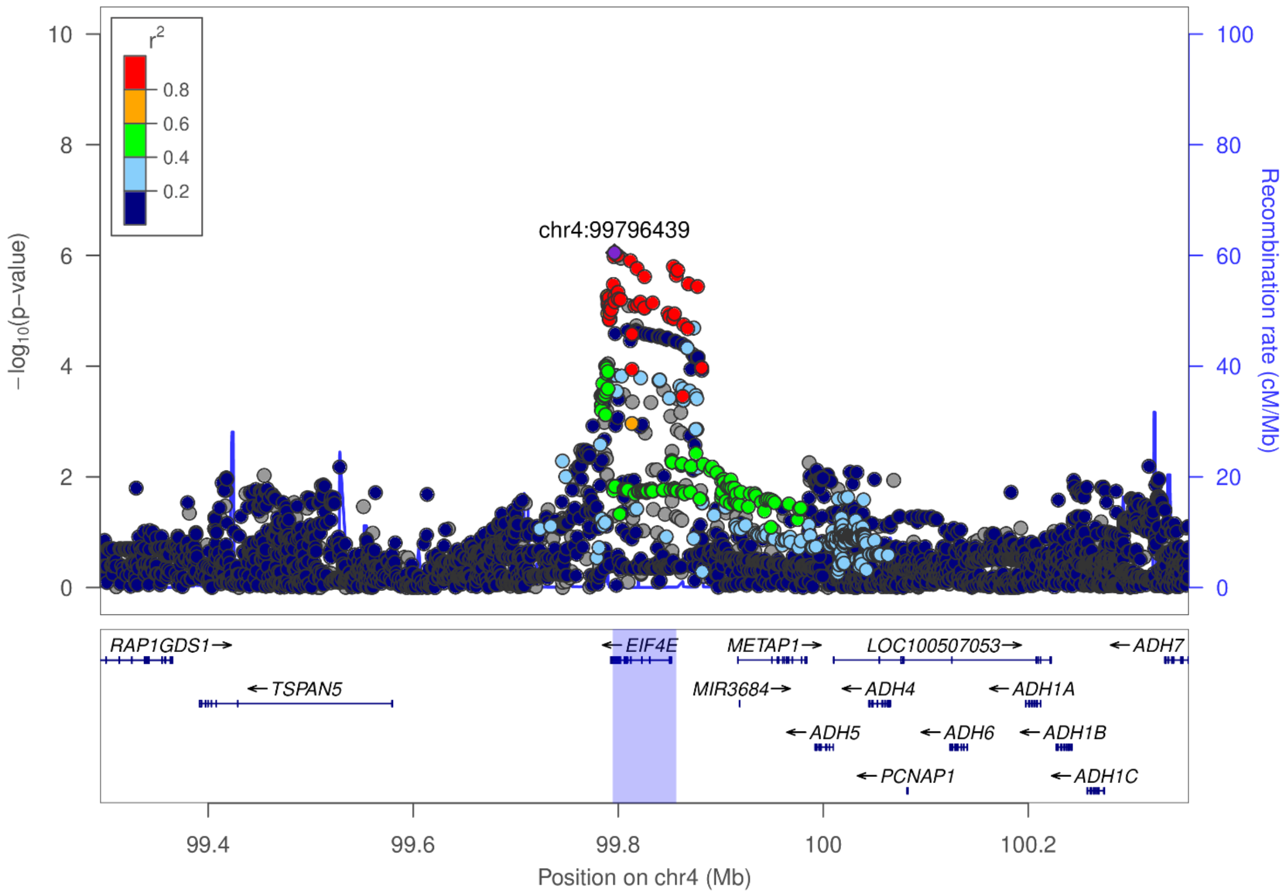

E: DKD, *INIP*

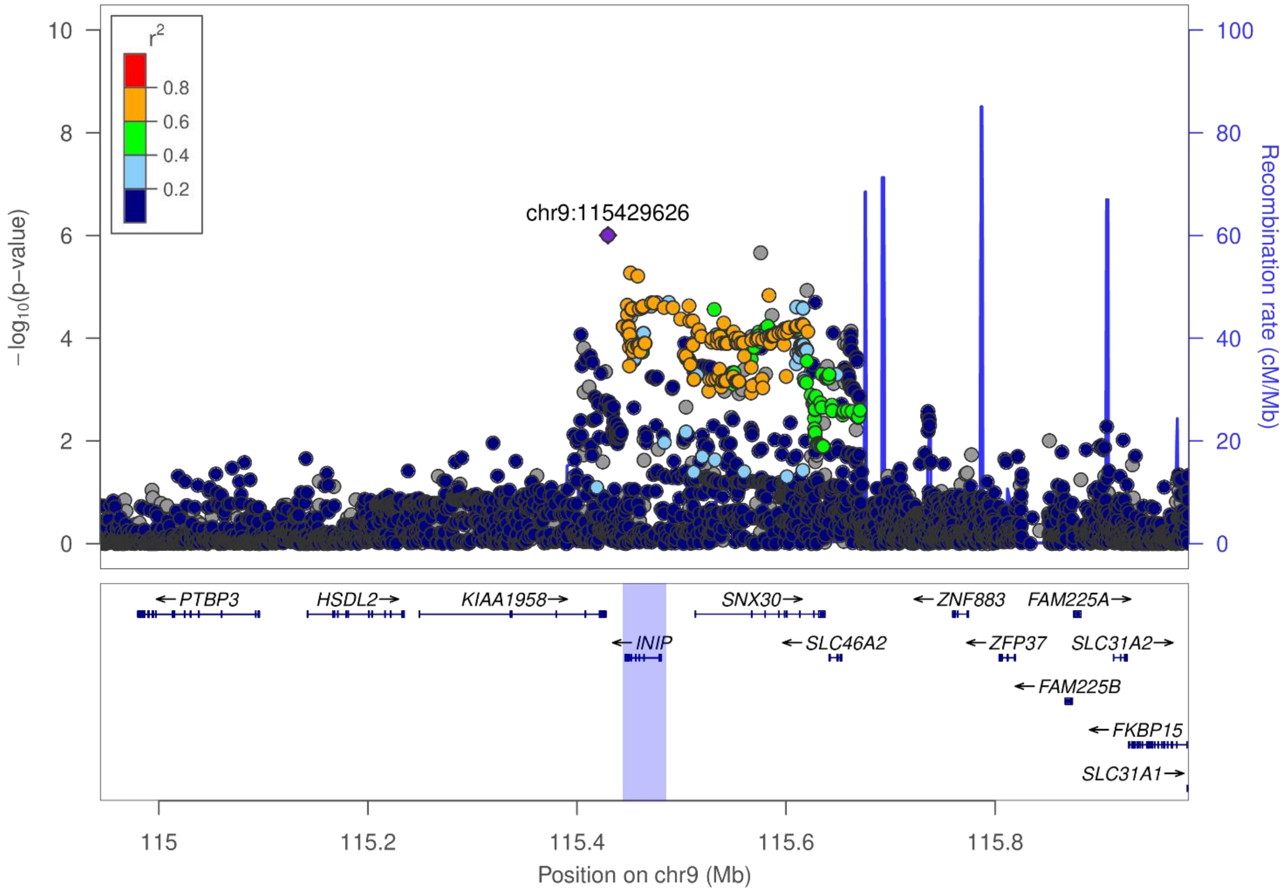

F: DKD, *SNX30*

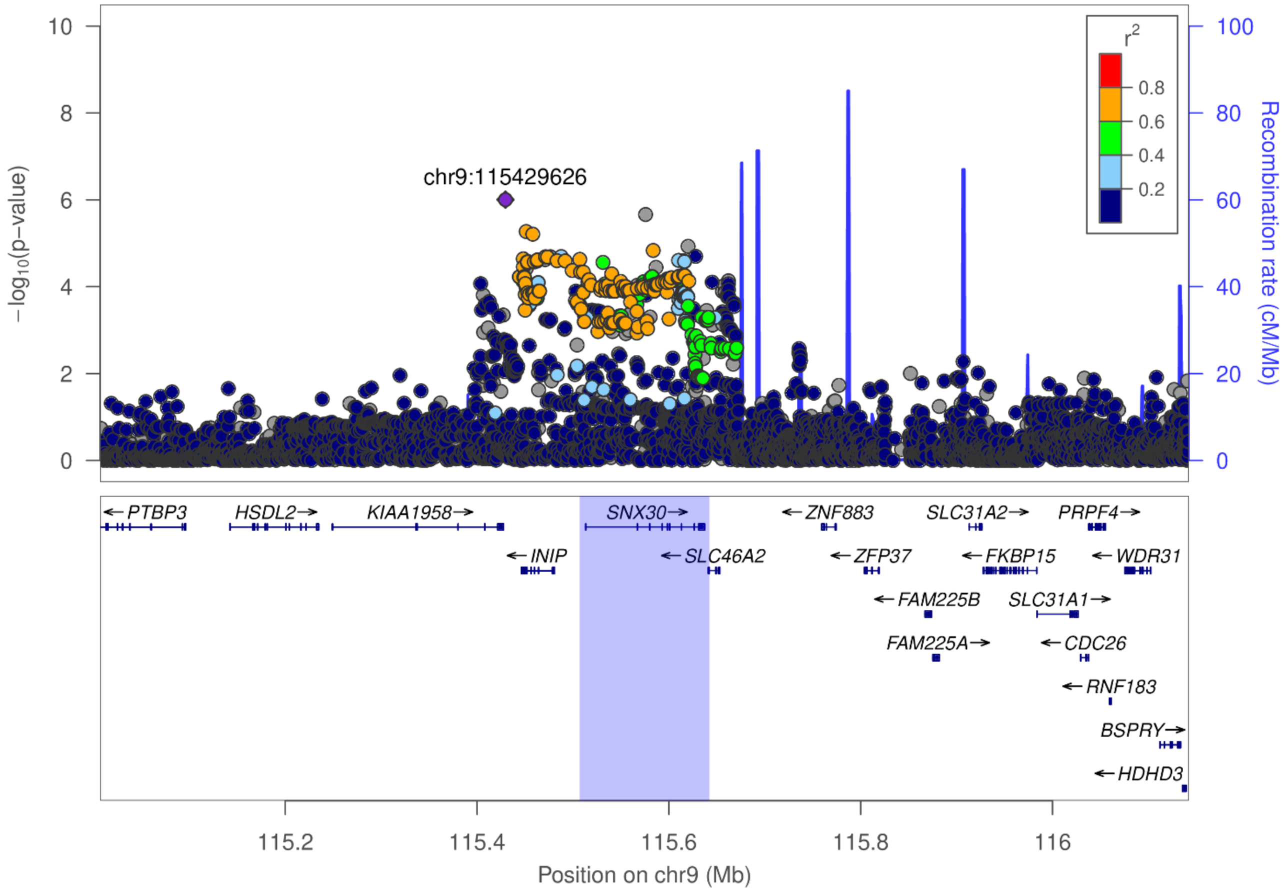

G: Severe DKD, *GPR158*

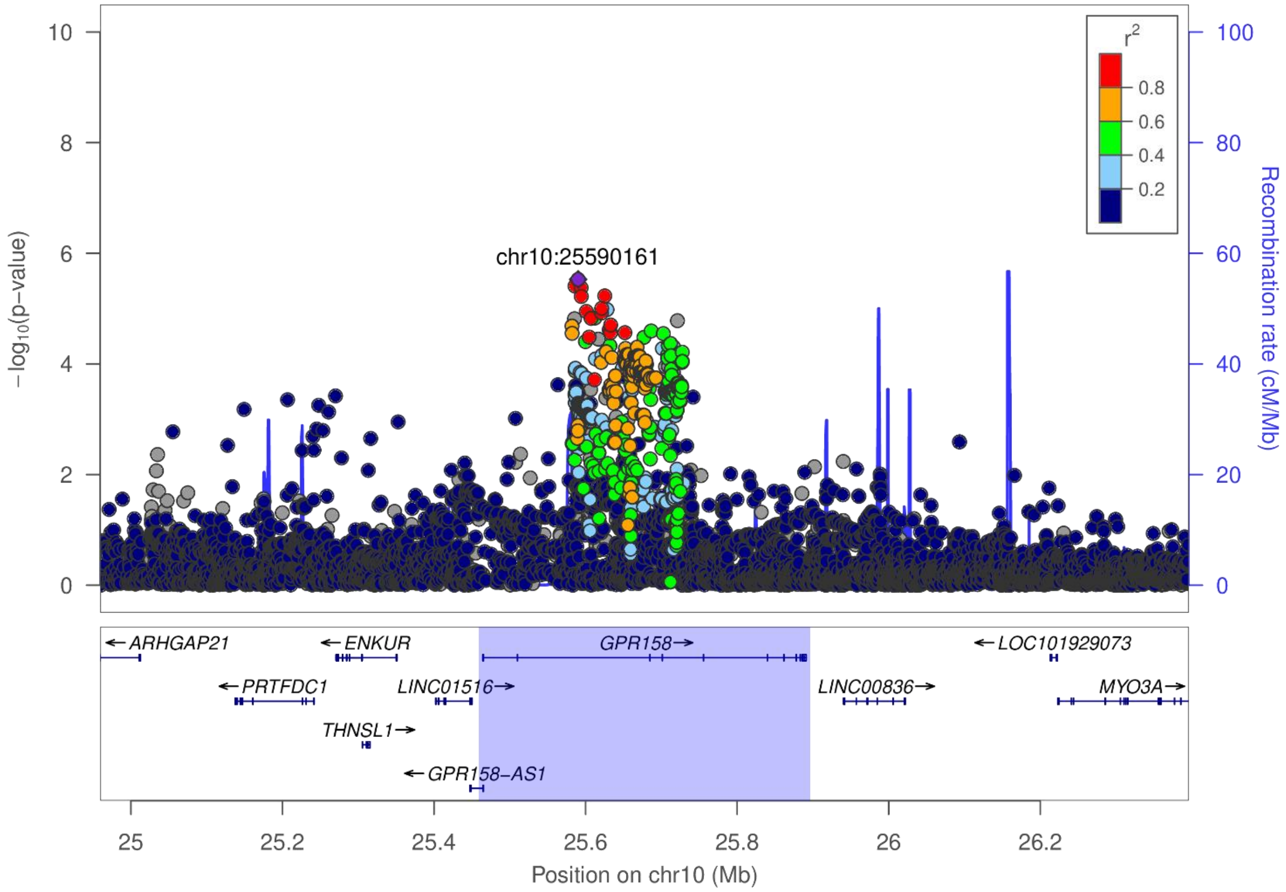

H: ESRD vs. macro, DCLK1

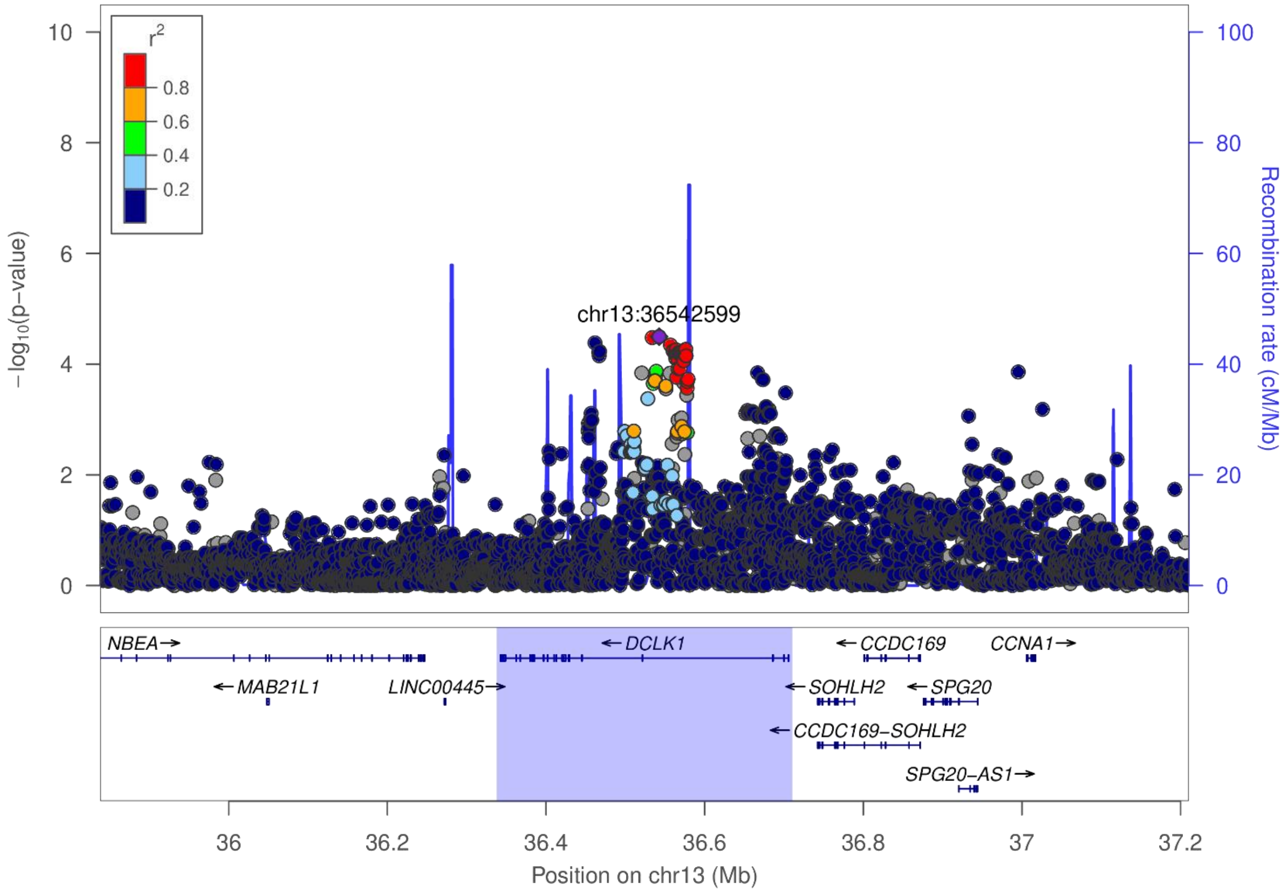

I: Severe DKD, *LSM14A*

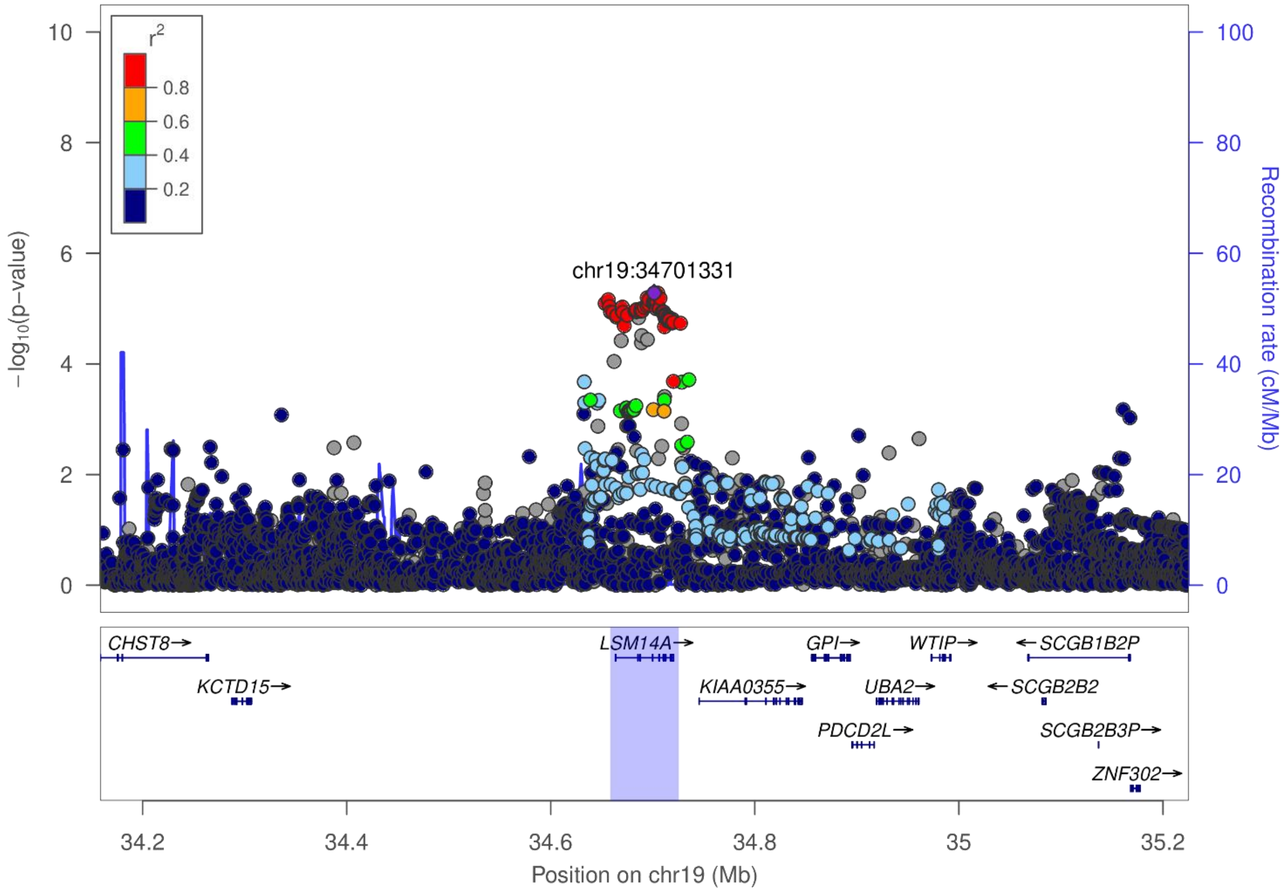

J: CKD extremes, COL20A1

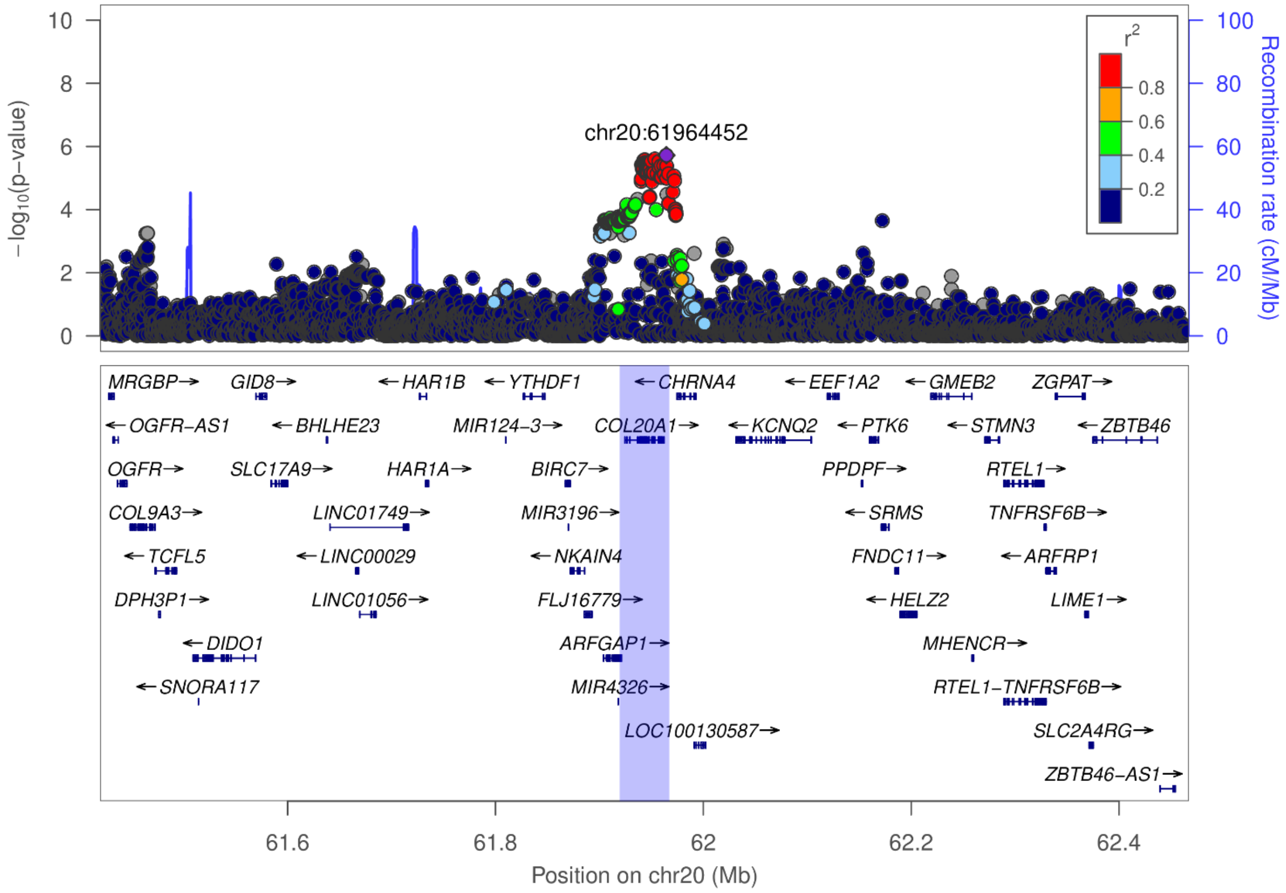

ESM Figure 7: TWAS QQ-plots and genomic control λGC inflation factors for A) tubular and B) glomerular expression.

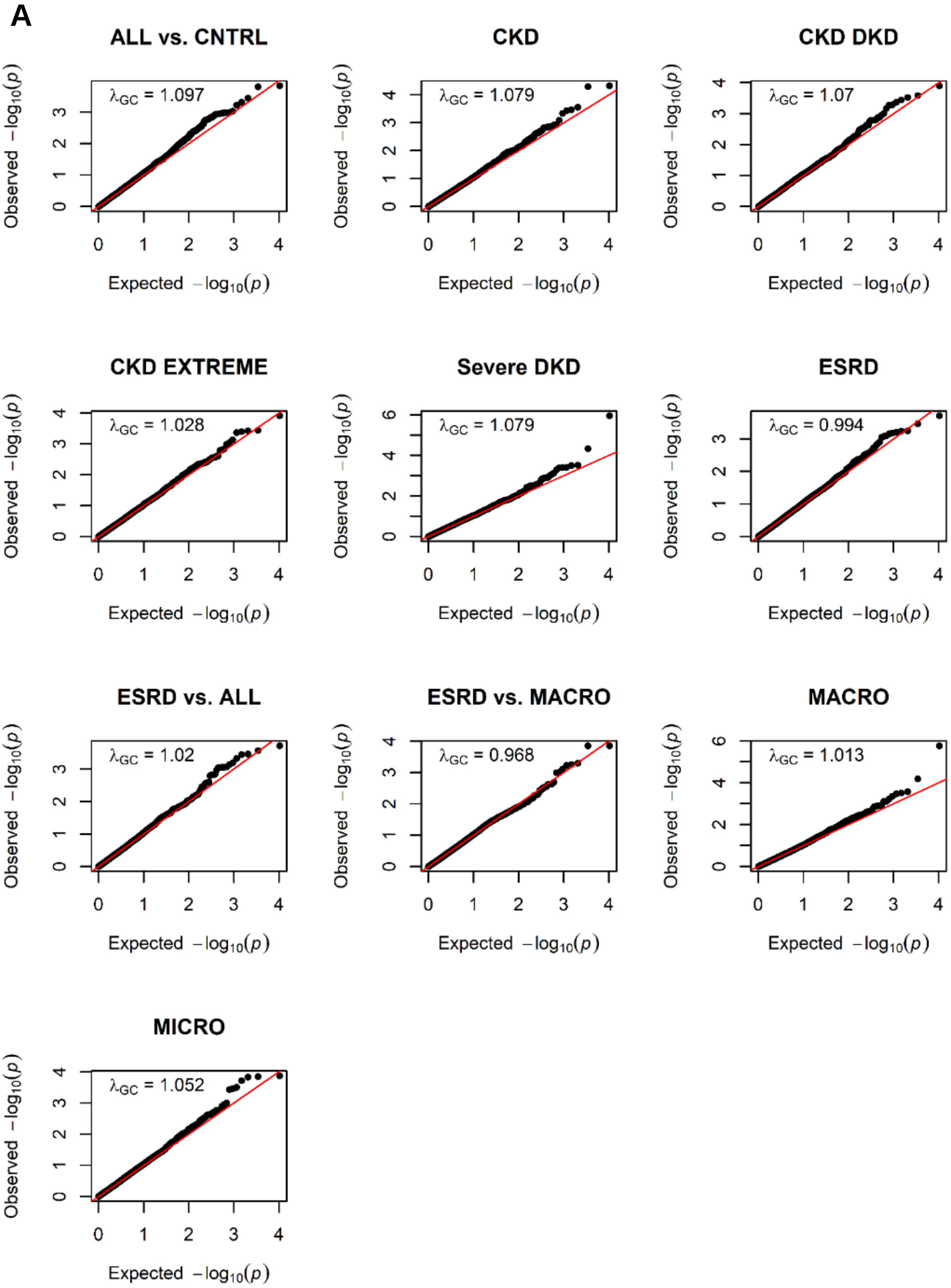

B

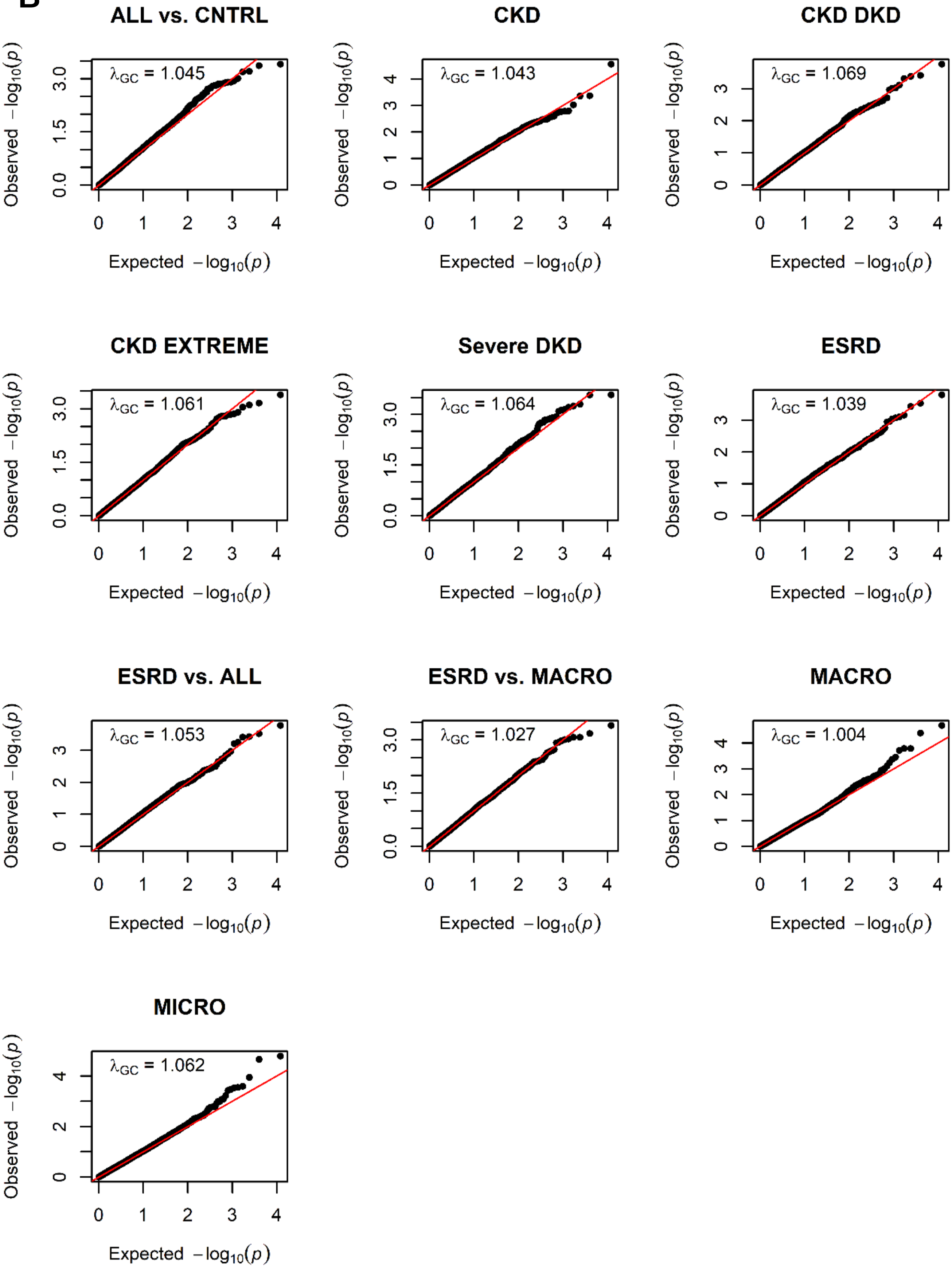
ALL vs. CNTRL
λGC = 1.045
CKD
λGC = 1.043
CKD DKD
λGC = 1.069
CKD EXTREME
λGC = 1.061
Severe DKD
λGC = 1.064
ESRD
λGC = 1.039
ESRD vs. ALL
λGC = 1.053
ESRD vs. MACRO
λGC = 1.027
MACRO
λGC = 1.004
MICRO
λGC = 1.062
Observed −log10(p)
Expected −log10(p)

ESM Figure 8: Tubular and glomerular gene expression of the lead genes correlate with multiple morphological and pathological renal parameters. Golden rectangles indicate glomerular gene expression, green ellipses tubular gene expression, and gray circles the morphological phenotypes. All nominally significant correlations are shown. Blue edges indicate negative correlation, red edges positive correlation. Correlation with fibrosis, Glomerulosclerosis (GlomScl), and eGFR are measured in the nephrectomy samples. B1_ and B2_ indicate phenotypes from the first and second renal biopsies (B1, B2, respectively) from the Pima Indians, correlated with gene expression in transcriptomic data from the corresponding time point. B1/2_GlomVol: glomerular volume; B1/2_GlomW: glomerular width; B1/2 FPW: podocyte foot process width. B1/2_ACR: albumin creatinine ratio; B1/2_Fibr: fibrosis; B1/2_HbA1c: HbA1c B1/2_MesVol: mesangial volume; B1/2_SV: Surface volume of peripheral glomerular basement membrane per glomerulus; B1_Slope: measured GFR (mGFR) slope between the B1 and B2. B1_DMesVol: change in mesangial volume between B1 and B2. B1_DSV: change in SV between B1 and B2.

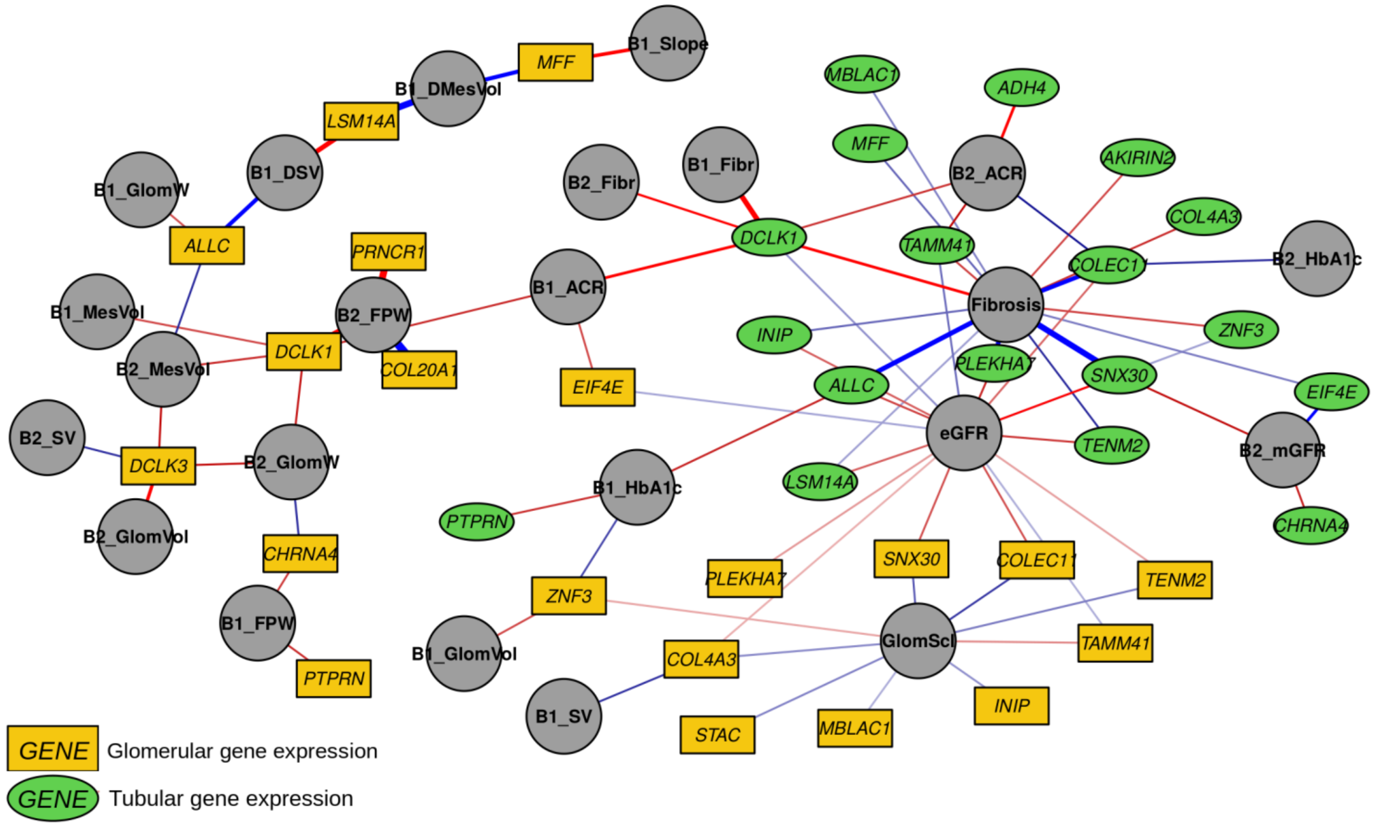

ESM Figure 9: Genetic correlation between DKD and related traits based on LD score regression. Only trait combinations with p<0.05 are shown, and traits that remained significant after correcting for 78 studied traits (p-value < 0.05/78 = 6.4×10-4) are indicated with dark dot borders. Dot colors indicate aging related (light purple), anthropometric (green; including BMI and obesity related (light green), height (dark green), and waist and/or hip related (pale green)), inflammatory bowel disease (orange), bone mineral density (light gray), coronary artery disease (red), glycemic (light blue), type 2 diabetes (dark blue), serum creatinine and cystatin C (brown), lipids (yellow), uric acid (purple), and smoking related (dark gray) traits.

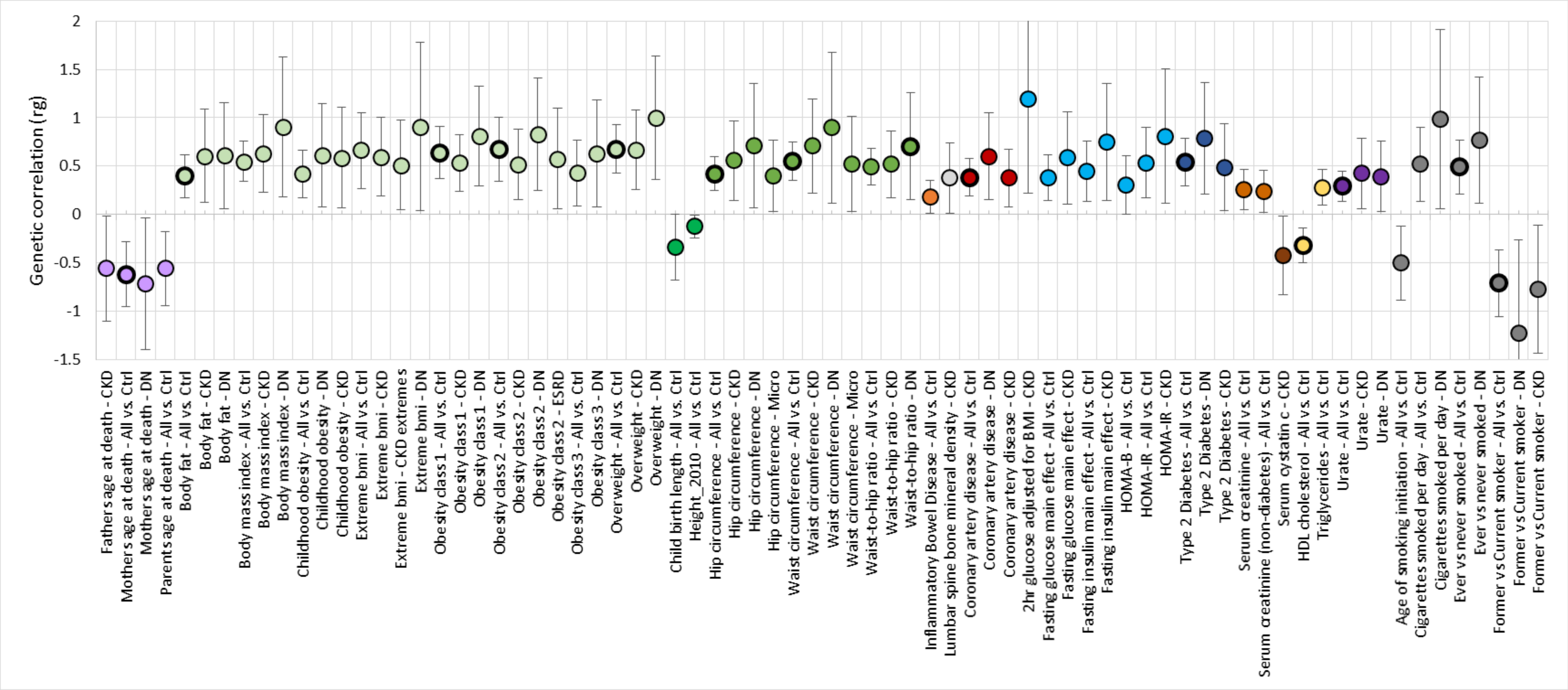

ESM Figure 10: Mendelian Randomization scatter plots for SNP effects for the metabolic traits vs. DKD (All vs. Ctrl). Lines indicate IVW, Weighted median, and MR egger coefficients.

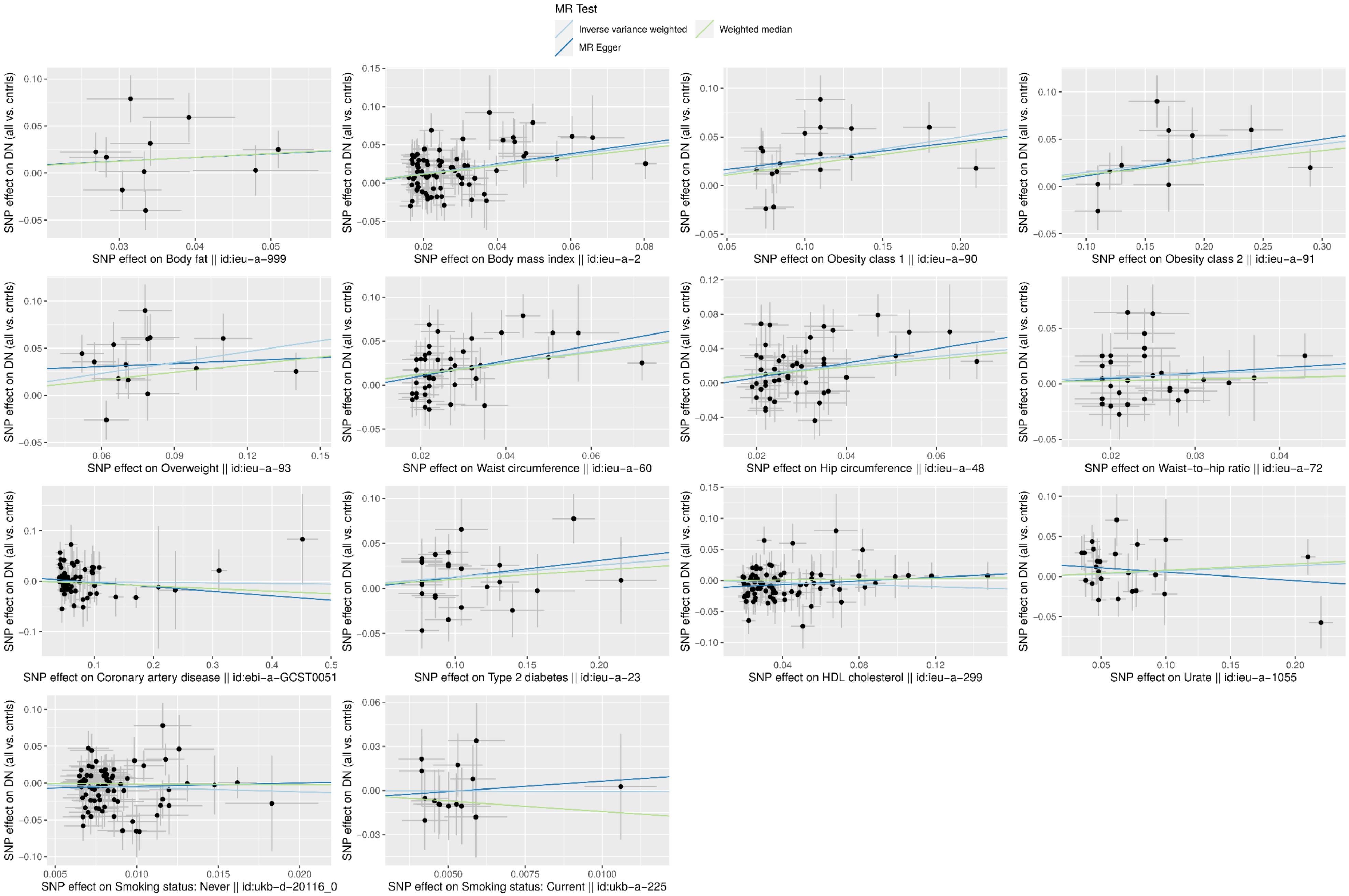

ESM Figure 11: rs1260634 intronic in the LSM14A gene affects the predicted binding motifs for KLF12, KLF4, and SP8 (top to bottom). Images obtained from the RegulomeDB (www.regulomedb.org)

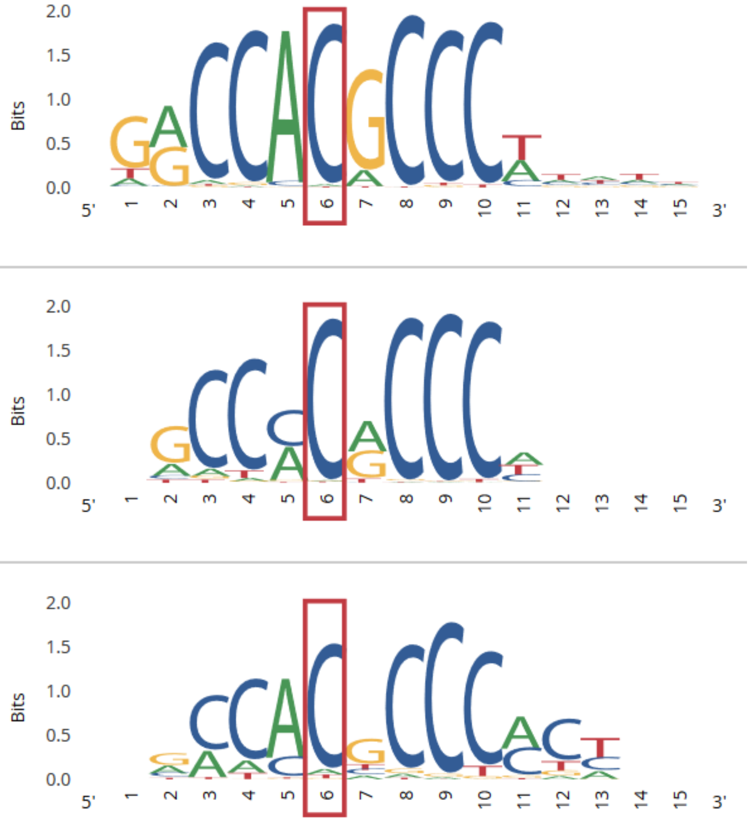

Supplement: Supplementary file 1 — (PDF 8564 kb) [file 125_2022_5735_MOESM1_ESM.pdf]
